# Inhibition of hepatic oxalate overproduction ameliorates metabolic dysfunction-associated steatohepatitis

**DOI:** 10.1038/s42255-024-01134-4

**Published:** 2024-09-27

**Authors:** Sandeep Das, Alexandra C. Finney, Sumit Kumar Anand, Sumati Rohilla, Yuhao Liu, Nilesh Pandey, Alia Ghrayeb, Dhananjay Kumar, Kelley Nunez, Zhipeng Liu, Fabio Arias, Ying Zhao, Brenna H. Pearson-Gallion, M. Peyton McKinney, Koral S. E. Richard, Jose A. Gomez-Vidal, Chowdhury S. Abdullah, Elizabeth D. Cockerham, Joseph Eniafe, Andrew D. Yurochko, Tarek Magdy, Christopher B. Pattillo, Christopher G. Kevil, Babak Razani, Md. Shenuarin Bhuiyan, Erin H. Seeley, Gretchen E. Galliano, Bo Wei, Lin Tan, Iqbal Mahmud, Ida Surakka, Minerva T. Garcia-Barrio, Philip L. Lorenzi, Eyal Gottlieb, Eduardo Salido, Jifeng Zhang, A. Wayne Orr, Wanqing Liu, Monica Diaz-Gavilan, Y. Eugene Chen, Nirav Dhanesha, Paul T. Thevenot, Ari J. Cohen, Arif Yurdagul, Oren Rom

**Affiliations:** 1 Department of Pathology and Translational Pathobiology, Louisiana State University Health Sciences Center-Shreveport, Shreveport, LA USA; 2 Department of Internal Medicine, Frankel Cardiovascular Center, University of Michigan, Ann Arbor, MI USA; 3 Department of Cancer Biology, University of Texas MD Anderson Cancer Center, Houston, TX USA; 4 Department of Molecular and Cellular Physiology, Louisiana State University Health Sciences Center-Shreveport, Shreveport, LA USA; 5 Institute of Translational Research, Ochsner Clinic Foundation, New Orleans, LA USA; 6 Department of Medicinal Chemistry and Molecular Pharmacology, College of Pharmacy, Purdue University, West Lafayette, IN USA; 7 Departamento de Química Farmacéutica y Orgánica, Facultad de Farmacia, Unidad de Excelencia de Química Aplicada a Biomedicina y Medioambiente, Granada, Spain; 8 Department of Microbiology and Immunology, Center of Applied Immunology and Pathological Processes, Feist-Weiller Cancer Center, Louisiana State University Health Sciences Center-Shreveport, Shreveport, LA USA; 9 Division of Cardiology and Vascular Medicine Institute, Department of Medicine, University of Pittsburgh School of Medicine and University of Pittsburgh Medical Center, Pittsburgh, PA USA; 10 Department of Chemistry, University of Texas at Austin, Austin, TX USA; 11 Department of Pathology, Ochsner Clinic Foundation, New Orleans, LA USA; 12 Metabolomics Core Facility, Department of Bioinformatics and Computational Biology, The University of Texas MD Anderson Cancer Center, Houston, TX USA; 13 Department of Pathology, Hospital Universitario de Canarias, Universidad de La Laguna, Centro de Investigación Biomédica en Red de Enfermedades Raras (CIBERER), Tenerife, Spain; 14 Department of Pharmaceutical Sciences and Department of Pharmacology, Wayne State University, Detroit, MI USA; 15 Multi-Organ Transplant Institute, Ochsner Clinic Foundation, New Orleans, LA USA

**Keywords:** Non-alcoholic steatohepatitis, Liver, Liver diseases, Metabolism

## Abstract

The incidence of metabolic dysfunction-associated steatohepatitis (MASH) is on the rise, and with limited pharmacological therapy available, identification of new metabolic targets is urgently needed. Oxalate is a terminal metabolite produced from glyoxylate by hepatic lactate dehydrogenase (LDHA). The liver-specific alanine-glyoxylate aminotransferase (AGXT) detoxifies glyoxylate, preventing oxalate accumulation. Here we show that AGXT is suppressed and LDHA is activated in livers from patients and mice with MASH, leading to oxalate overproduction. In turn, oxalate promotes steatosis in hepatocytes by inhibiting peroxisome proliferator-activated receptor-α (PPARα) transcription and fatty acid β-oxidation and induces monocyte chemotaxis via C–C motif chemokine ligand 2. In male mice with diet-induced MASH, targeting oxalate overproduction through hepatocyte-specific AGXT overexpression or pharmacological inhibition of LDHA potently lowers steatohepatitis and fibrosis by inducing PPARα-driven fatty acid β-oxidation and suppressing monocyte chemotaxis, nuclear factor-κB and transforming growth factor-β targets. These findings highlight hepatic oxalate overproduction as a target for the treatment of MASH.

## Abstract (web summary)

Genetic and pharmacological inhibition of the overproduction of oxalate in the liver alleviates metabolic dysfunction-associated steatohepatitis in male mice.

## Main

Metabolic dysfunction-associated steatotic liver disease (MASLD) has become the most common chronic liver disease worldwide, affecting an alarming one-third of the global population^1^. MASLD encompasses a spectrum of liver pathologies, ranging from simple hepatic steatosis, metabolic dysfunction-associated steatohepatitis (MASH) that may be accompanied by hepatic fibrosis, and ultimately cirrhosis, which is associated with late-stage liver disease and may lead to hepatocellular carcinoma and liver failure^2,3^. Central to the pathogenesis of MASH, hepatic lipid overload occurs with excessive uptake of fatty acids released from the adipose tissue, enhanced de novo lipogenesis from excessive carbohydrate intake or inhibition of fatty acid β-oxidation (FAO)^3–5^. These lead to the accumulation of reactive oxygen species, mitochondrial dysfunction and endoplasmic reticulum stress^4^, which culminate in lipotoxicity. Consequently, inflammasome activation leads to the release and accumulation of proinflammatory mediators, which facilitate leucocyte infiltration and hepatic stellate cell activation, promoting MASH and hepatic fibrosis^2–4^. Despite major advances in our understanding of the metabolic and molecular mechanisms driving MASH, and considerable efforts in the development of drugs targeting lipid and carbohydrate metabolism, there is limited pharmacological therapy available for the treatment of MASH^6,7^. Therefore, identifying alternative dysregulated metabolic pathways that can be targeted for MASH treatment is urgently needed.

While abnormal metabolism of lipids and carbohydrates are known features of MASLD^8^, accumulating evidence highlights impaired amino acid metabolism as a causative factor in the pathogenesis of MASH. Notably, lower circulating glycine is consistently reported in association with worsened MASH features in humans and mouse models and its deficiency accelerates the disease in mice^9–12^. The liver-specific enzyme, alanine-glyoxylate aminotransferase (AGXT), plays a central role in catalysing glycine formation from glyoxylate^12–14^ and recent transcriptomics studies from our group and others consistently reported suppression of *AGXT* in livers from patients and mice with MASH^12,15–17^. AGXT loss-of-function results in the accumulation of glyoxylate^18^, which can also be produced from glycolate via glycolate oxidase (GO) and is rapidly converted by hepatic lactate dehydrogenase (LDHA) to the terminal end product, oxalate^19^. While our recent studies demonstrated that the loss of AGXT exacerbates diet-induced MASH in mice^12^, the hepatic regulation of oxalate metabolism and its levels in humans and mice with MASH, the effects of oxalate accumulation in hepatocytes, the primary cells responsible for its formation and the therapeutic potential of targeting impaired oxalate metabolism in MASH, have not been systematically studied.

Considering the consistent reports of suppressed *AGXT* in MASH^12,15–17^, recent evidence linking AGXT dysfunction and primary hyperoxaluria with liver disease^12,15,20,21^, the known deleterious effects of oxalate in renal and cardiovascular diseases^22–26^, together with the rising prevalence of MASH and limited therapy available^1,6,7^, there is a strong rationale to better understand impaired oxalate metabolism in MASH and to evaluate the therapeutic potential of targeting this dysregulated metabolic pathway. In the current study, we systematically evaluated the metabolic regulation and levels of hepatic oxalate in multiple well-defined human cohorts, MASH mouse models, and hepatocellular in vitro systems. Furthermore, we determined the effects of oxalate on molecular drivers of MASH within hepatocytes regulating lipid metabolism, mitochondrial function and inflammatory responses. Finally, utilizing hepatocyte-specific overexpression of AGXT and pharmacological inhibition of LDHA and GO combined with transcriptomics, lipidomics and functional assays, we defined the protective effects of genetic and pharmacological targeting of oxalate overproduction in MASH and the underlying mechanisms behind this approach.

## Results

### Hepatic oxalate overproduction in human and mice with MASH

**Fig. 1:**
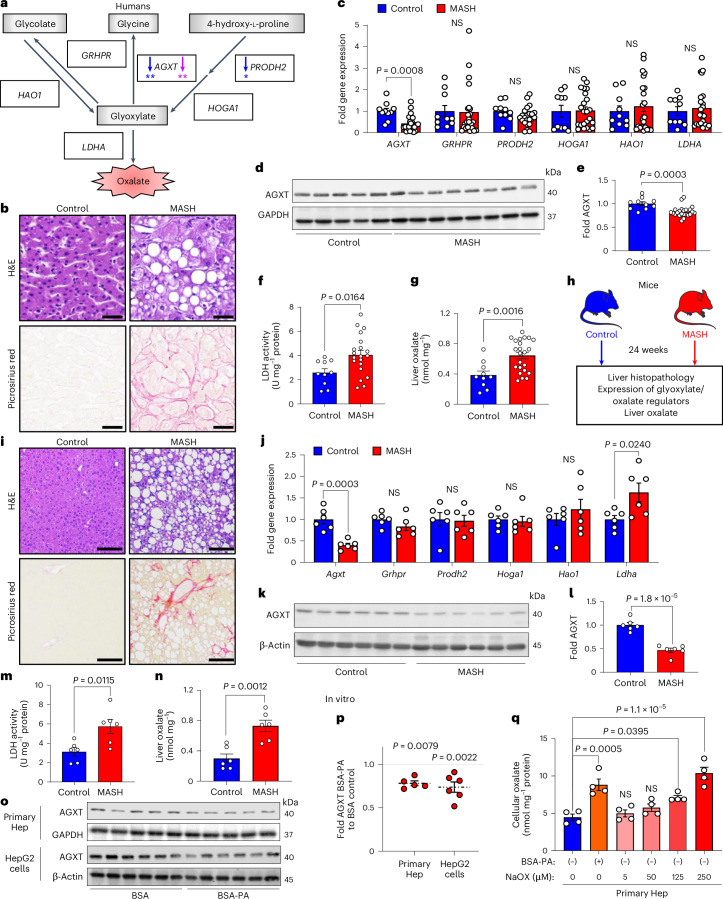
AGXT is suppressed and LDHA is activated leading to oxalate overproduction in livers from humans and mice with MASH. **a**, Schema of glyoxylate metabolism and oxalate formation. Spearman’s correlations were calculated between the expression of genes regulating glyoxylate metabolism or oxalate formation and hepatic fat in livers from transplantation donors (*n* = 206, GSE26106). A significant inverse correlation is denoted by blue arrows (*P < 0.05, **P < 0.01). The association between the expression of genes regulating glyoxylate metabolism or oxalate formation and MASH was assessed through regression models and a meta-analysis based on transcriptomics of livers from patients with or without MASH (MASH, *n* = 104; control, *n* = 44, GSE83452; MASH, *n* = 24; control, *n* = 24, GSE61260). A significant inverse association is denoted by purple arrows (**P < 0.01). **b**, H&E and Picrosirius red staining of liver samples obtained from patients with end-stage MASH (*n* = 22) compared with healthy donors as control (*n* = 10). **c**–**g**, Expression of *AGXT*, *GRHPR*, *PRODH2*, *HOGA1*, *HAO1* and *LDHA* relative to *GAPDH* (**c**), protein abundance (**d**) and quantification of AGXT relative to GAPDH (**e**), from liver samples of MASH patients (*n* = 23) and control (*n* = 10), LDH activity, from liver samples of patients with MASH (*n* = 20) and control (*n* = 10) (**f**) and oxalate concentrations normalized to tissue weight in liver samples from patients with end-stage MASH (*n* = 23) and controls (*n* = 10) (**g**). **h**,**i**, Liver samples were collected from C57BL/6J mice fed a standard chow diet (control, *n* = 6) or a high-fat, high-fructose, high-cholesterol diet (MASH diet, *n* = 6) for 24 weeks (**h**) and stained with H&E and Picrosirius red (**i**). **j**–**n**, Expression of *Agxt*, *Grhpr*, *Prodh2*, *Hoga1*, *Hao1* and *Ldha* relative to *Gapdh* (**j**), protein abundance (**k**) and quantification of AGXT relative to β-actin (**l**), LDH activity (**m**) and oxalate concentrations normalized to tissue weight in liver samples from mice (**n**) with (*n* = 6) or without MASH (*n* = 6). **o**, Primary hepatocytes (Hep) from mice fed a standard chow diet and HepG2 cells were treated with either BSA-conjugated PA (200 µM) or BSA control overnight. **p**, Protein abundance and quantification of AGXT relative to GAPDH (primary hepatocytes, *n* = 5) or β-actin (HepG2 cells, *n* = 6). **q**, Intracellular oxalate normalized to protein concentrations in primary hepatocytes treated with PA (200 µM) or increasing concentrations of sodium oxalate (NaOX; 0–250 µM, *n* = 4). For primary hepatocytes, each point represents an individual mouse. For HepG2 cells, each point represents an independent experiment that included at least two biological repetitions. The samples derived from the same experiment and blots were processed in parallel for **d**,**e**. All data are expressed as mean ± s.e.m. Statistical comparisons were made using two-tailed unpaired *t*-test (**c**,**f**,**j**,**l**,**m**), Mann–Whitney *U*-test (**c**,**e**,**g**,**j**,**n**,**p**) or one-way ANOVA with Tukey’s multiple comparisons test (**q**). All individual points and *P* values are shown. A *P* value < 0.05 was considered statistically significant; NS, not significant. Scale bars, 200 µm. Parts of **h** were drawn by using pictures from Servier Medical Art (licensed under a Creative Commons Attribution 3.0 Unported License at https://creativecommons.org/licenses/by/3.0). Source data

**Extended Data Fig. 1:**
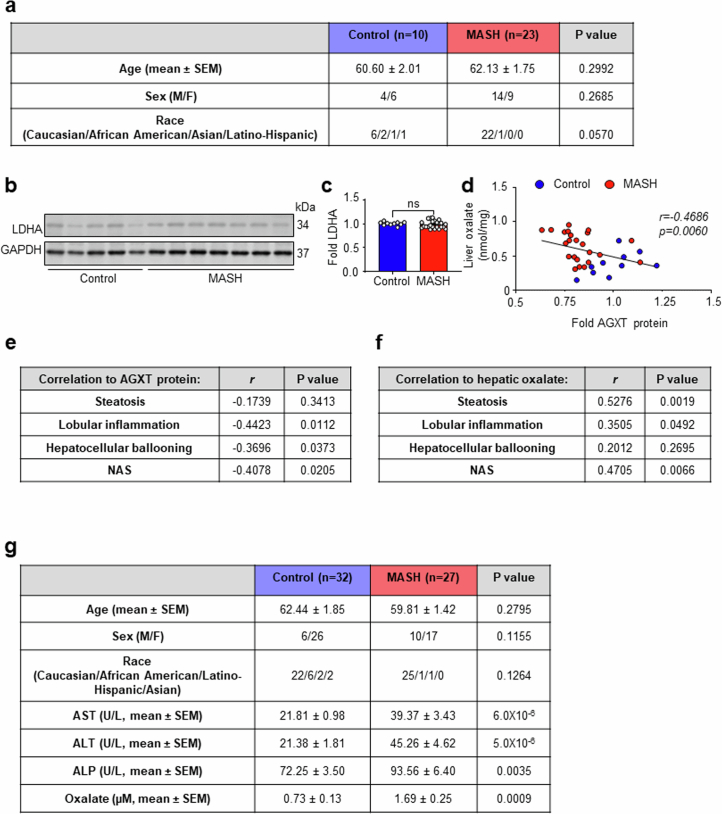
Dysregulated oxalate metabolism in human MASH. **a**, Distribution of age, sex (M, male; F, female), and race among patients with end-stage MASH (n=23) or healthy donors (Control, n=10). **b**, protein abundance and (**c**) quantification of LDHA relative to GAPDH in liver specimens from patients with MASH (n=23) or controls (n=10). **d**, Spearman’s correlation between the relative abundance of AGXT protein and liver oxalate in samples from patients with MASH (n=23) and controls (n=10). **e**, Liver sections from patients with MASH (n=22) and controls (n=10) were scored for steatosis, lobular inflammation, hepatocellular ballooning, and NAS, then correlated with the relative abundance of AGXT protein, and (**f**) liver oxalate concentrations. **g**, Distribution of age, sex (M, male; F, female), and race, and levels of aspartate aminotransferase (AST), alanine aminotransferase (ALT), alkaline phosphatase (ALP), and oxalate in plasma samples from patients with (n=27) or without (Control, n=32) MASH. The samples were derived from the same experiment and blots were processed in parallel for (**b**, **c**). All data are expressed as mean ± SEM. Statistical comparisons were made using two-sided Chi-squared test (**a**, **g**), two-tailed Mann–Whitney U test (**a**, **c**, **g**), or Spearman’s correlation (**d**-**f**). A p value <0.05 was considered statistically significant. Source data

As the first step in exploring the dysregulated oxalate metabolism in MASH, we assessed the hepatic expression of key regulators of glyoxylate metabolism and oxalate formation in well-defined human cohorts. As illustrated in Fig. 1a, glycolate, glycine and 4-hydroxy-l-proline are the precursors for glyoxylate, which is readily oxidized to oxalate by LDHA^19^. Although humans have no enzymes capable of degrading oxalate, specific hepatic enzymes can prevent oxalate overproduction via glyoxylate detoxification^19,27^. Beyond its conversion to glycine by AGXT, glyoxylate can also be converted to glycolate via glycolate reductase/hydroxypyruvate reductase (GRHPR). In an opposite reaction, glycolate can be oxidized to glyoxylate by GO (encoded by *HAO1*). In addition, hydroxyproline dehydrogenase (HYPDH; encoded by *PRODH2*) converts 4-hydroxy-l-proline to 4-hydroxy-2-oxoglutarate, which is further catalysed into glyoxylate by 4-hydroxy-2-oxoglutarate aldolase 1 (HOGA1)^19^. We first determined the association between the expression of the above genes and hepatic steatosis in a cohort of 206 liver transplantation donors (GSE26106)^12,28,29^. This revealed an inverse association between the expression of *AGXT* and *PRODH2* with hepatic fat content, with the most significant negative correlation found for *AGXT* (*AGXT*: *r* = −0.1838, *P* = 0.0083; *PRODH2*: *r* = −0.1530, *P* = 0.0296; Fig. 1a). We further assessed the association between the expression of genes regulating glyoxylate metabolism or oxalate formation and MASH through regression models and a meta-analysis based on transcriptomics of livers from patients with or without MASH (GSE83452 and GSE61260)^30,31^. This meta-analysis revealed that among all the glyoxylate/oxalate metabolic genes, only *AGXT* expression was significantly and inversely associated with MASH (*β* = −0.141, *P* = 0.0089; Fig. 1a). We next assessed dysregulated oxalate metabolism in liver specimens obtained from a cohort of patients with histologically confirmed end-stage MASH compared with healthy donors (Fig. 1b) through quantitative PCR with reverse transcription (qRT–PCR), western blot and enzymatic activity analyses. There were no significant differences in race, age or sex between the patients with MASH and controls (Extended Data Fig. 1a). qRT–PCR analyses demonstrated a significant downregulation of *AGXT* in liver specimens from patients with MASH, without significant changes in other genes regulating glyoxylate metabolism or oxalate formation (*GRHPR*, *PRODH2*, *HOGA1*, *HAO1* and *LDHA*; Fig. 1c). Additionally, we found a significant decrease in AGXT protein abundance in livers from patients with MASH (Fig. 1d,e). Although the mRNA and protein levels of LDHA were not significantly altered (Fig. 1c and Extended Data Fig. 1b,c), LDHA activity was significantly enhanced in patients with MASH (Fig. 1f). Aligned with suppressed AGXT and enhanced LDHA activity, oxalate concentrations assessed using an enzymatic method were significantly increased in livers from patients with MASH compared with controls (Fig. 1g). Notably, a significant inverse correlation was found between AGXT protein abundance and liver oxalate levels (Extended Data Fig. 1d) as well as MASH severity as determined by blinded histopathological analysis of the NAFLD activity score (NAS; Extended Data Fig. 1e). In contrast, a significant positive correlation was found between hepatic oxalate concentrations and MASH severity (Extended Data Fig. 1f). We further assessed the levels of plasma oxalate in another cohort of patients diagnosed with MASH compared to healthy controls. Compared with controls, patients with MASH had significantly elevated levels of plasma transaminases and oxalate with no significant differences in race, age or sex (Extended Data Fig. 1g).

**Fig. 2:**
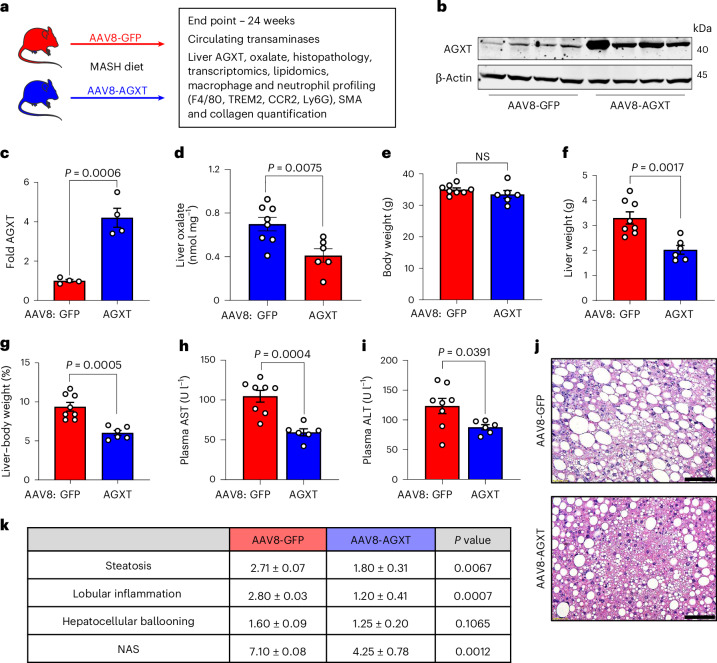
Oxalate lowering via hepatocyte-specific overexpression of AGXT ameliorates MASH. **a**, Male C57BL/6J mice were injected with AAV8-TBG-GFP or AAV8-TBG-AGXT (2 × 10^11^ viral genomes per mouse) and placed on the MASH diet for 24 weeks before end point analyses. **b**,**c**, Protein abundance (**b**) and quantification (**c**) of AGXT relative to β-actin in liver samples from mice treated with AAV8-GFP (*n* = 4) or AAV-AGXT (*n* = 4). **d**, Oxalate concentrations normalized to tissue weight in liver samples from mice treated with AAV8-GFP (*n* = 8) or AAV8-AGXT (*n* = 6). **e**–**g**, Body weight (**e**), liver weight (**f**) and liver-to-body weight ratios (**g**) in mice treated with AAV8-GFP (*n* = 8) or AAV8-AGXT (*n* = 6). **h**,**i**, Plasma samples were analysed for AST (**h**) and ALT (**i**) in mice treated with AAV8-GFP (*n* = 8) or AAV8-AGXT (*n* = 6). **j**,**k**, Liver samples were sectioned and stained with H&E (**j**) and scored for steatosis, lobular inflammation, hepatocellular ballooning and NAS (**k**) from mice treated with AAV8-GFP (*n* = 8) or AAV8-AGXT (*n* = 6). All data are expressed as mean ± s.e.m. Statistical comparisons were made using two-tailed unpaired *t*-test (**c**,**e**–**i**,**k**), or Mann–Whitney *U*-test (**d**,**k**). A *P* value < 0.05 was considered statistically significant. Scale bars, 200 µm. Parts of **a** were drawn by using pictures from Servier Medical Art. Servier Medical Art by Servier is licensed under a Creative Commons Attribution 3.0 Unported License at https://creativecommons.org/licenses/by/3.0/. Source data

**Extended Data Fig. 2:**
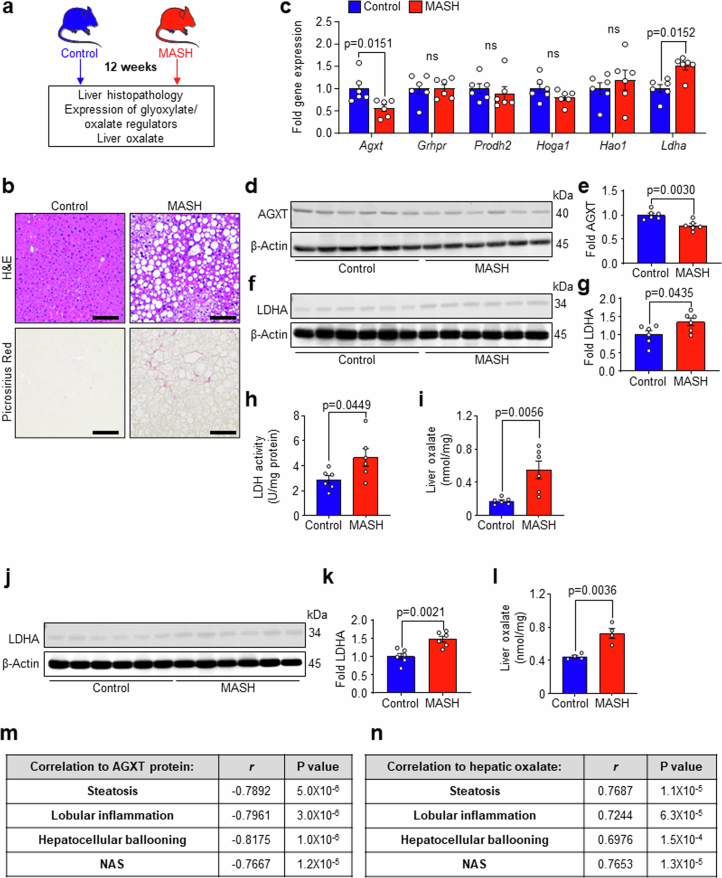
Dysregulated oxalate metabolism in murine MASH. **a**, Liver samples were collected from C57BL/6J mice fed a standard chow diet (Control, n=6) or a high-fat, high-fructose, high-cholesterol diet (MASH diet, n=6) for 12 weeks, and (**b**) stained with H&E and Picrosirius Red. **c**, Expression of *Agxt*, *Grhpr*, *Prodh2*, *Hoga1*, *Hao1*, and *Ldha* relative to *Gapdh*, (**d, e**) protein abundance and quantification of AGXT, and (**f, g**) LDHA relative to β-Actin, (**h**) LDH activity, and (**i**) oxalate concentrations normalized to tissue weight in liver samples from mice with (n=6) or without early MASH (12 weeks, n=6). **j**, Protein abundance and (**k**) quantification of LDHA in liver samples from mice with or without advanced MASH (24 weeks, n=6). **l**, Ion chromatography coupled with mass spectrometry (IC-MS) validation of oxalate concentrations normalized to tissue weight in liver samples from mice with or without MASH (24 weeks, n=4). **m**, Liver sections from mice fed the standard chow or MASH diet for 12 weeks (n=6) or 24 weeks (n=6) were scored for steatosis, lobular inflammation, hepatocellular ballooning, and NAS, then correlated with the relative abundance of AGXT protein, and (**n**) liver oxalate concentrations. Statistical comparisons were made using two-tailed unpaired t-test (**c**, **e**, **g**, **h**, **i**, **k**, **l**), Mann–Whitney U test (**c**), or Spearman’s correlation (**m**, **n**). A p value <0.05 was considered statistically significant. Scale bars = 200 µm. Parts of Extended Data Fig. 2a were drawn by using pictures from Servier Medical Art. Servier Medical Art by Servier is licensed under a Creative Commons Attribution 3.0 Unported License (https://creativecommons.org/licenses/by/3.0/. Source data

**Extended Data Fig. 3:**
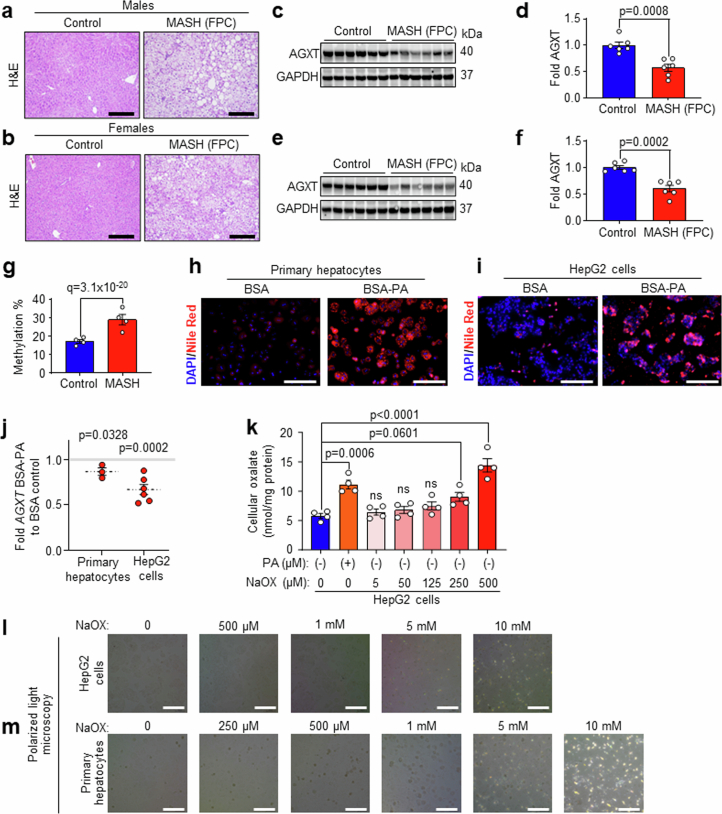
Suppression and hypermethylation of AGXT in MASH and optimization of sodium oxalate studies in hepatocytes. Liver samples were collected from (**a**) male and (**b**) female mice fed a standard chow diet (Control) or the fructose-palmitate-cholesterol (FPC) diet for 4 months and stained with H&E. Protein abundance and quantification of AGXT relative to GAPDH in livers from males (**c, d**) and female mice (**e, f**) with and without MASH (n=6). (**g**) Methylation analysis using the methylKit R package (methylKit_1.28.0) from mice with advanced MASH (24 weeks on the MASH diet) or controls (n=4). **h**, Neutral lipids visualized by Nile Red staining (red) and nuclei labelled with DAPI (blue) in primary hepatocytes from mice fed a standard chow diet (n=3) and HepG2 cells (n=4) (**i**) treated with either BSA-conjugated palmitic acid (BSA-PA, 200 µM) or BSA control overnight. **j**, Expression of *Agxt* relative to *Gapdh* in primary mouse hepatocytes (n=3), and of AGXT relative to GAPDH in HepG2 cells (n=6). **k**, Intracellular oxalate normalized to protein concentrations in HepG2 cells treated with PA (200 µM) or increasing concentrations of sodium oxalate (NaOX, 0–500 µM, n=4). For primary hepatocytes, each point represents an individual mouse. For HepG2 cells, each point represents an independent experiment that included at least 2 biological repetitions. **l**, Assessment of calcium-oxalate deposition as birefringent crystals detected by polarization microscopy in HepG2 cells (n=4), and primary hepatocytes (n=4) (**m**) treated overnight with increasing concentrations of NaOX (0–10 mM). All data are expressed as mean ± SEM. Statistical comparisons were made using two-tailed unpaired t-test (**d**, **f**, **j**), Fisher’s exact test for pairwise comparison (**g**), or one-way ANOVA with Tukey’s multiple comparisons test (**k**). All individual points and p values are shown. A p value <0.05 was considered statistically significant. Scale bars = 200 µm. Source data

We next sought to determine whether suppressed AGXT, activated LDHA and the ensuing accumulation of oxalate are consistently observed in livers from mice with MASH. We fed male C57BL/6J mice a standard chow diet (control) or a high-fat, high-fructose, high-cholesterol diet (MASH diet) for 12 or 24 weeks (Fig. 1h and Extended Data Fig. 2a). This dietary model is known to induce early MASH featuring hepatic steatosis, but mild inflammation and fibrosis at week 12 and advanced steatohepatitis and fibrosis at week 24, which mimics the human disease^12,32–34^, as we confirmed using hematoxylin and eosin (H&E) and Picrosirius red staining (Fig. 1i and Extended Data Fig. 2b). The liver mRNA (Extended Data Fig. 2c) and protein abundance (Extended Data Fig. 2d,e) of AGXT were significantly decreased already in mice with early MASH. Notably, the mRNA (Extended Data Fig. 2c) and protein abundance (Extended Data Fig. 2f,g) of LDHA were significantly increased, together with enhanced lactate dehydrogenase (LDH) activity (Extended Data Fig. 2h), resulting in a significant increase in hepatic oxalate (Extended Data Fig. 2i). In livers from mice with advanced MASH, the suppression of AGXT (Fig. 1j–l), the upregulation of LDHA (Fig. 2j and Extended Data Fig. 2j,k), enhanced LDH activity (Fig. 1m) and the increased hepatic oxalate (Fig. 1n), were more significant. Moreover, we validated the significant increase in hepatic oxalate concentrations in MASH using ion chromatography coupled with mass spectrometry (IC–MS; Extended Data Fig. 2l). Similar to humans, AGXT protein abundance inversely correlated (Extended Data Fig. 2m), whereas hepatic oxalate positively correlated (Extended Data Fig. 2n), with MASH severity in mice. To confirm that the suppression of AGXT in mice with MASH is not diet- or sex-specific, we further assessed the protein abundance of AGXT in male and female mice fed the fructose-palmitate-cholesterol (FPC) diet for 16 weeks, another established model of MASH^35^, as we confirmed histologically (Extended Data Fig. 3a,b). A significant decrease in AGXT protein abundance was found in livers from both male (Extended Data Fig. 3c,d) and female (Extended Data Fig. 3e,f) mice with MASH induced by the FPC diet. It was recently reported that *AGXT* is downregulated due to hypermethylation in hepatic steatosis^15^, and we sought to determine whether hypermethylation mediates suppressed AGXT in advanced MASH. Applying unbiased genome-wide analysis of DNA methylation through reduced representation bisulfite sequencing (RRBS), we found a significant increase in methylation at the *Agxt* promoter and first exon in livers from mice with advanced MASH (Supplementary Fig. 1 and Extended Data Fig. 3g). Last, we sought to determine if lipid loading in hepatocytes is sufficient to suppress AGXT and enhance oxalate accumulation in vitro. We isolated primary hepatocytes from mice, treated them with 200 µM of BSA-conjugated palmitic acid (PA) or BSA (control) and confirmed the accumulation of lipids in the hepatocytes (Extended Data Fig. 3h,i). Following PA treatment, primary hepatocytes showed a significant reduction in mRNA (Extended Data Fig. 3j) and protein abundance (Fig. 1o,p) of AGXT. These observations were consistent in the HepG2 human hepatoma cell line, which largely retains the biochemical pathways of glyoxylate metabolism^36^ and also demonstrated a significant reduction in the mRNA (Extended Data Fig. 3j) and protein abundance of AGXT (Fig. 1o,p) in response to PA treatment. Accordingly, lipid loading induced a significant accumulation of intracellular oxalate in both primary hepatocytes (Fig. 1q) and HepG2 cells (Extended Data Fig. 3k), which was comparable to treatment with exogenous sodium oxalate (NaOX) at 250 µM and 500 µM, respectively. Notably, polarized light microscopy revealed that treatment of primary hepatocytes and HepG2 cells with 250 µM and 500 µM NaOX, respectively, did not result in the formation of oxalate crystals, which were clearly visible in cells treated with ≥5 mM of NaOX (Extended Data Fig. 3l,m). Taken together, these comprehensive studies in multiple human and mouse cohorts as well as in lipid-loaded hepatocytes uncover suppressed hepatic AGXT and enhanced LDHA activity leading to oxalate overproduction in both humans and mice with MASH.

### Oxalate lowering via AGXT overexpression ameliorates MASH

**Extended Data Fig. 4:**
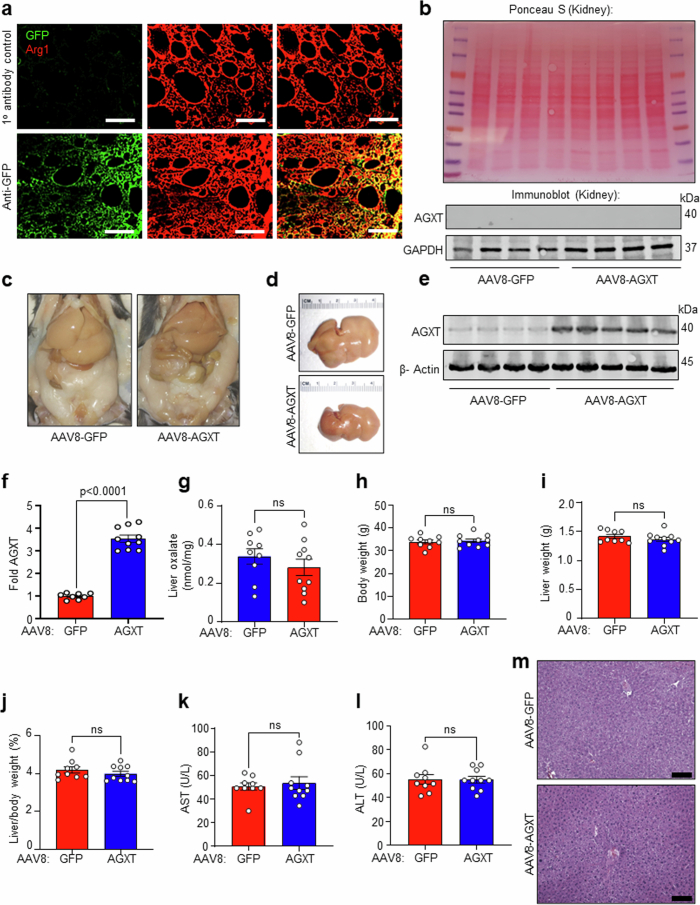
Hepatocyte-specific overexpression of AGXT on MASH or standard chow diet. Male C57BL/6J mice were injected with AAV8-TBG-GFP or AAV8-TBG-AGXT (2x10^11^ viral genomes per mouse) and placed on the MASH diet for 24 weeks prior to end point analyses. **a**, Livers from mice injected with AAV8-TBG-GFP (n=4) were sectioned and assessed by immunofluorescence for GFP (green) expression in hepatocytes (Arg1, red, scale bar = 50 μm). **b**, Kidney lysates from mice injected with AAV8-GFP or AAV8-AGXT were assessed for AGXT by Western blot (n=4). Equal protein loading was verified using GAPDH expression and Ponceau S staining. **c**, Representative gross appearance of the abdominal cavity from mice treated with AAV8-GFP (n=8) or AAV8-AGXT (n=6). **d**, Representative gross appearance of the livers from mice treated with AAV8-GFP (n=8) or AAV8-AGXT (n=6). Male C57BL/6J mice were injected with AAV8-TBG-GFP or AAV8-TBG-AGXT (2x1011 viral genomes per mouse) and placed on the standard chow diet for 12 weeks prior to end point analyses. **e**, Protein abundance, and (**f**) quantification of AGXT relative to β-Actin in liver samples from mice treated with AAV8-GFP (n=8) or AAV8-AGXT (n=10). **g**, Oxalate normalized to liver tissue weight in samples from mice treated with AAV8-GFP (n=9) or AAV8-AGXT (n=10). **h**, Body weight, (**i**) liver weight, and (**j**) liver-to-body weight ratios in mice treated with AAV8-GFP (n=9) or AAV8-AGXT (n=10). **k**, Plasma samples were analysed for aspartate transaminase (AST), and (**l**) alanine transaminase (ALT) in mice treated with AAV8-GFP (n=9) or AAV8-AGXT (n=10). **m**, Liver samples from mice treated with AAV8-GFP (n=9) or AAV8-AGXT (n=10) were sectioned and stained with H&E (scale bar = 200 μm). The samples were derived from the same experiment and blots were processed in parallel for (**e**, **f**). All data are expressed as mean ± SEM. Statistical comparisons were made using two-tailed unpaired t-test (**f**-**j**, **l**), or Mann–Whitney U test (**k**). All individual points and p values are shown. A p value <0.05 was considered statistically significant. Source data

Considering that AGXT is consistently downregulated in MASH leading to hepatic oxalate overproduction in association with disease severity, and that the loss of AGXT accelerates MASH progression in mice^12^, we next sought to determine whether overexpression of AGXT in hepatocytes would improve MASH outcomes by lowering oxalate production. To this end, we injected C57BL/6J mice with AAV8-AGXT or AAV8-GFP control driven by the hepatocyte-specific thyroxine binding globulin (TBG) promoter^24^ and placed the mice on the MASH diet for 24 weeks (Fig. 2a). At the end point, we confirmed that TBG-driven green fluorescent protein (GFP) expression was only observed in hepatocytes (Extended Data Fig. 4a) and that there was no overexpression of AGXT in extrahepatic tissues following AAV8-AGXT injection (Extended Data Fig. 4b). We further confirmed the overexpression of AGXT (Fig. 2b,c) aligned with a significant decrease in oxalate (Fig. 2d) in livers from mice treated with AAV8-AGXT. The mice were further assessed for alterations in body weight, liver weight and circulating transaminases. While mice overexpressing AGXT had no significant differences in body weight (Fig. 2e), they showed significantly reduced liver weights (Fig. 2f) and liver-to-body weight ratios (Fig. 2g) compared with mice treated with AAV8-GFP. Accordingly, plasma aspartate aminotransferase (AST; Fig. 2h) and alanine aminotransferase (ALT; Fig. 2i), established biomarkers of liver injury, were significantly decreased in mice overexpressing AGXT in hepatocytes. Despite no obvious differences in adiposity based on body weight (Fig. 2e) and gross morphology of the abdominal cavity (Extended Data Fig. 4c), livers from mice overexpressing AGXT were markedly smaller than livers from mice treated with AAV8-GFP (Extended Data Fig. 4c,d). Histopathological analyses based on H&E staining (Fig. 2j) demonstrated significant reductions in the scores of hepatic steatosis, lobular inflammation and the overall NAS (Fig. 2k). Notably, in mice fed the standard chow diet, hepatocyte-specific overexpression of AGXT (Extended Data Fig. 4e,f) led to a mild reduction in hepatic oxalate, albeit without statistical significance (Extended Data Fig. 4g). Accordingly, no significant differences in liver damage indices, including the liver-to-body weight ratio (Extended Data Fig. 4h–j), plasma AST (Extended Data Fig. 4k) and ALT (Extended Data Fig. 4l), as well as liver histology (Extended Data Fig. 4m) were noted between mice that were treated with AAV8-AGXT or AAV8-GFP on the standard chow diet. Altogether, these data indicate that lowering the overproduction of hepatic oxalate in MASH through hepatocyte-specific overexpression of AGXT potently lowers the disease severity.

### AGXT overexpression curbs hepatic steatosis by inducing FAO

**Fig. 3:**
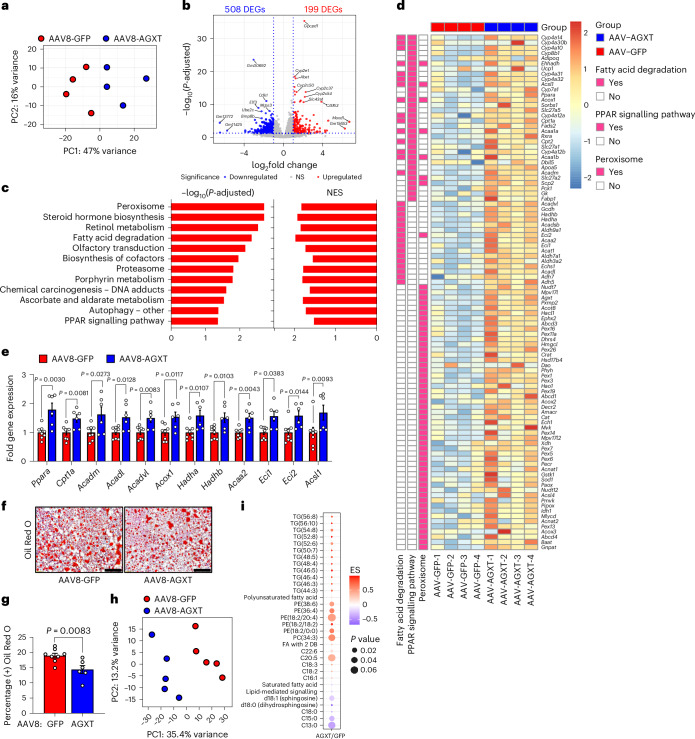
Oxalate lowering via hepatocyte-specific overexpression of AGXT curbs hepatic steatosis through induction of fatty acid β-oxidation pathways. Male C57BL/6J mice were injected with AAV8-TBG-GFP or AAV8-TBG-AGXT (2 × 10^11^ viral genomes per mouse) and placed on the MASH diet for 24 weeks before end point analyses. **a**, PCA was performed based on RNA sequencing of livers from mice treated with AAV8-GFP or AAV8-AGXT (*n* = 4). **b**, Volcano plot of DEGs significantly upregulated (red) or downregulated (blue) in livers from mice treated with AAV8-AGXT compared to AAV8-GFP based on RNA sequencing (*n* = 4). **c**, Pathways significantly enriched in the upregulated DEGs and normalized enrichment scores (NES), based on KEGG pathway analysis comparing livers from mice treated with AAV8-GFP or AAV8-AGXT (*n* = 4). **d**, Heatmap of DEGs related to FAO pathways comparing livers from mice treated with AAV8-GFP or AAV8-AGXT (*n* = 4; colour bar, log_2_ fold change in AAV8-AGXT versus AAV-GFP). **e**, qRT–PCR validation of selected FAO-related DEGs relative to *Gapdh* in livers from mice treated with AAV8-GFP (*n* = 8) or AAV8-AGXT (*n* = 6). **f**, Liver samples were collected from mice treated with AAV8-GFP (*n* = 8) or AAV8-AGXT (*n* = 6) and stained with Oil Red O (red) and Harris hematoxylin nuclear counterstain. **g**, Percent-positive Oil Red O area from mice treated with AAV8-GFP (*n* = 8) or AAV8-AGXT (*n* = 6). **h**, PCA was performed based on untargeted lipidomics of livers from mice treated with AAV8-GFP or AAV8-AGXT (*n* = 5). **i**, Representation of lipid species increased (red) or decreased (blue) in livers from mice treated with AAV8-AGXT compared with AAV8-GFP (*n* = 5; colour bar, enrichment score (ES)). All data are expressed as mean ± s.e.m. Statistical comparisons were made using a two-tailed unpaired *t*-test (**e**,**g**) or Mann–Whitney *U*-test (**e**). A two-sided Wald test was used to identify DEGs (**b**). The significance of the enriched pathways (**c**) was determined by a right-tailed Fisher’s exact test followed by Benjamini–Hochberg multiple testing adjustment. All individual points and *P* values are shown. *P* < 0.05 was considered statistically significant. Scale bars, 200 µm. Source data

**Extended Data Fig. 5:**
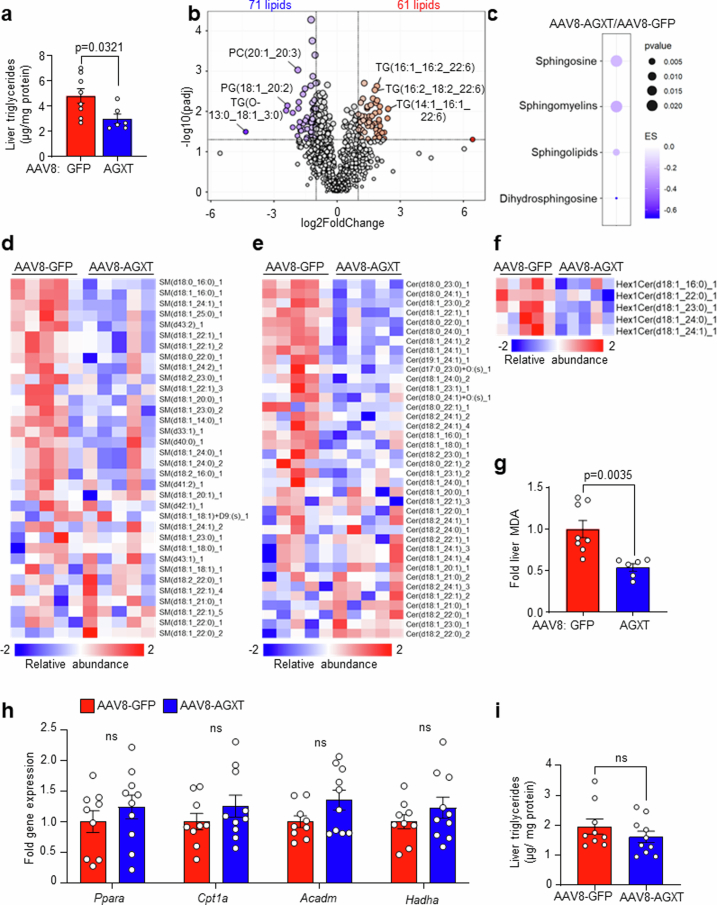
Effects of hepatocyte-specific overexpression of AGXT on hepatic lipids on MASH or standard chow diet. Male C57BL/6J mice were injected with AAV8-TBG-GFP or AAV8-TBG-AGXT (2x10^11^ viral genomes per mouse) and placed on the MASH diet for 24 weeks prior to end point analyses. **a**, Liver triglycerides normalized to protein concentrations in livers from mice treated with AAV8-GFP (n=8) or AAV8-AGXT (n=6). **b**, Volcano plot of lipid metabolites significantly increased (red) or decreased (blue) in livers from mice treated with AAV8-AGXT compared to AAV8-GFP based on untargeted lipidomics (n=5). **c**, Pathway enrichment analysis (scale bar = enrichment score, ES), and heatmaps of (**d**) sphingomyelins, (**e**) ceramides, and (**f**) hexosylceramides in livers from mice treated with AAV8-AGXT compared to AAV8-GFP based on untargeted lipidomics (n=5, scale bar: relative abundance). **g**, Relative liver malondialdehyde (MDA) levels normalized to protein concentrations in mice treated with AAV8-GFP (n=8) or AAV8-AGXT (n=6). **h**, Male C57BL/6J mice were injected with AAV8-TBG-GFP or AAV8-TBG-AGXT (2x10^11^ viral genomes per mouse) and placed on a standard chow diet for 12 weeks prior to end point analyses. qRT-PCR analyses of *Ppara* and its target genes relative to *Gapdh* in livers from mice treated with AAV8-GFP (n=9) or AAV8-AGXT (n=10). **i**, Liver triglycerides normalized to protein concentrations in livers from mice treated with AAV8-GFP (n=9) or AAV8-AGXT (n=10). All data are expressed as mean ± SEM. Statistical comparisons were made using two-tailed unpaired t-test (**a**-**c**, **g**, **h**), or Mann–Whitney U test (**i**). All individual points and p values are shown. A p value <0.05 was considered statistically significant. Source data

To explore potential mechanisms by which oxalate lowering via AGXT overexpression ameliorates MASH, we performed unbiased RNA sequencing of livers from mice treated with AAV8-GFP or AAV8-AGXT following 24 weeks on the MASH diet. Principal-component analysis (PCA) revealed that the gene expression pattern of livers from mice treated with AAV8-AGXT was distinct from those treated with AAV8-GFP (Fig. 3a). Volcano plot representation showed over 700 differentially expressed genes (DEGs), with 508 significantly reduced and 199 significantly elevated in livers from mice overexpressing AGXT compared with mice treated with AAV8-GFP (Fig. 3b). Among the genes that were most significantly upregulated by AGXT overexpression were members of the cytochrome P450 CYP2 family such as *Cyp2c37*, *Cyp2c50*, *Cyp2c54* and *Cyp2e1* that regulate FAO and polyunsaturated fatty acid (PUFA) metabolism^37,38^ and *Cd163*, a known marker of anti-inflammatory macrophages^39^. In contrast, among the genes that were most significantly downregulated by AGXT overexpression were regulators of fibrotic and inflammatory responses in MASH, including E74-like factor 3 (*Elf3*)^40^ and bone morphogenetic protein 8b (*Bmp8b*)^41^. KEGG pathway analysis showed a most significant enrichment in the peroxisome pathway together with other key pathways related to FAO, including fatty acid degradation and peroxisome proliferator-activated receptor (PPAR) signalling pathways (Fig. 3c). RNA sequencing analysis (Fig. 3d), validated by qRT–PCR analyses (Fig. 3e), revealed that key genes driving hepatic FAO, including *Ppara*, the master regulator of FAO^42^, PPARγ coactivator-1α (*Ppargc1a*) and numerous PPARɑ target genes (carnitine palmitoyltransferase 1A, (*Cpt1a*), acyl-CoA dehydrogenase, medium chain (*Acadm*), acyl-CoA dehydrogenase, long chain (*Acadl*), acyl-CoA dehydrogenase very long chain (*Acadvl*), acyl-CoA oxidase 1 (*Acox1*), hydroxyacyl-CoA dehydrogenase subunit α (*Hadha*), *Hadhb*, acetyl-CoA acyltransferase 2 (*Acaa2*) and acyl-CoA synthetase long chain 1 (*Acsl1*))^42^ were significantly upregulated in livers from mice overexpressing AGXT. Because enhanced utilization of fatty acids can reduce hepatic lipid accumulation, we next measured neutral lipids using Oil Red O staining of liver sections from mice treated with AAV8-AGXT or AAV8-GFP and fed the MASH diet. Consistent with the above transcriptional alterations, livers from mice overexpressing AGXT had significantly reduced neutral lipid accumulation (Fig. 3f,g). Furthermore, biochemical analysis of liver lysates confirmed a significant reduction in hepatic triglycerides with AGXT overexpression (Extended Data Fig. 5a). To thoroughly assess the effects of AGXT overexpression during MASH on lipid and fatty acid composition, we further employed untargeted lipidomics. PCA revealed that the global lipidome in livers from mice treated with AAV8-AGXT was distinct from those treated with AAV8-GFP (Fig. 3h). Out of 315 lipid metabolites detected and annotated, 132 were significantly different, with 71 significantly reduced and 61 significantly elevated in livers from mice overexpressing AGXT compared with mice treated with AAV8-GFP (Extended Data Fig. 5b). Notably, while saturated fatty acids were decreased, PUFAs were increased in livers from mice overexpressing AGXT (Fig. 3i). Moreover, proinflammatory and lipotoxic sphingolipid metabolites implicated in MASLD/MASH, including sphingomyelins, ceramides and hexosylceramides^3,43,44^, were significantly decreased in livers from mice overexpressing AGXT (Fig. 3i and Extended Data Fig. 5c–f). In addition, livers from mice overexpressing AGXT had significantly lower levels of the lipid peroxidation marker, malondialdehyde (MDA), assessed by the thiobarbituric acid reactive substances (TBARS) assay (Extended Data Fig. 5g). Notably, in mice fed the standard chow diet, hepatocyte-specific overexpression of AGXT did not significantly upregulate *Ppara* and its target genes regulating FAO (Extended Data Fig. 5h) or lowered hepatic triglycerides (Extended Data Fig. 5i). Taken together, these findings indicate that lowering oxalate overproduction in MASH via AGXT overexpression attenuates hepatic steatosis through induction of FAO.

### Oxalate induces steatosis via suppression of PPARα and FAO

**Fig. 4:**
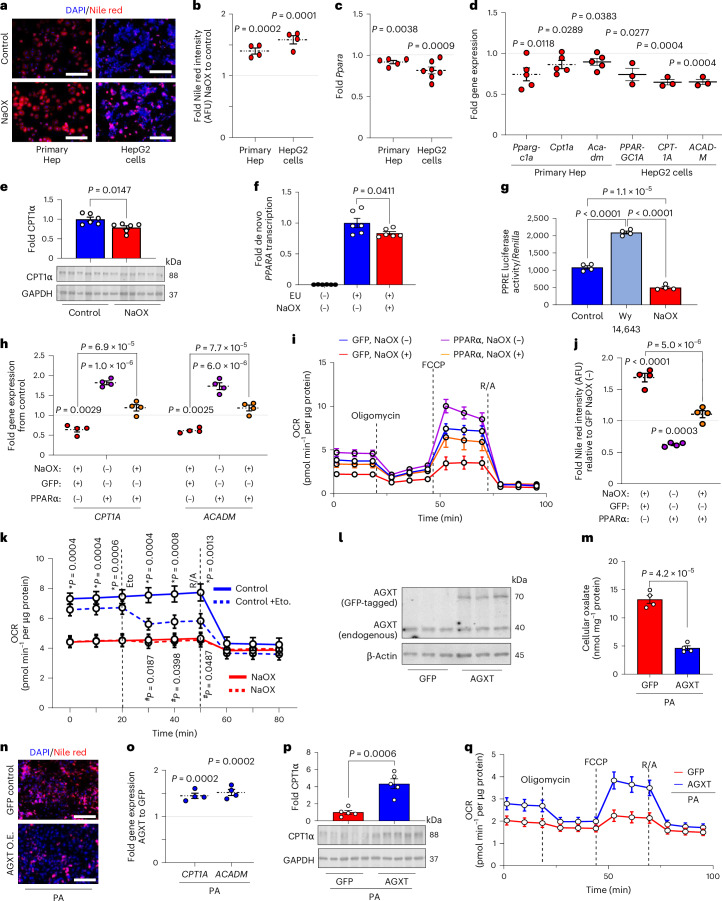
Oxalate induces lipid accumulation in hepatocytes by suppressing PPARα-regulated FAO. Primary hepatocytes (Hep) isolated from mice fed a standard chow diet (*n* = 4) and HepG2 cells (*n* = 4) were treated with or without sodium oxalate (NaOX; 250 μM, primary mouse hepatocytes; 500 μM, HepG2 cells). **a**, Neutral lipids were visualized with Nile red stain (red) and nuclei were labelled with DAPI (blue). **b**, Intensity of Nile red staining was normalized to number of nuclei (DAPI) and expressed as fold change. AFU, arbitrary fluorescence units. **c**,**d**, Expression of *PPARA* (*n* = 5–7) (**c**) and its target genes regulating fatty acid β-oxidation (**d**) in primary hepatocytes (relative to *Gapdh*, *n* = 5) and in HepG2 cells (relative to *GAPDH*, *n* = 3) treated with or without NaOX. **e**, Protein abundance and quantification of CPT1α relative to GAPDH in HepG2 cells treated with and without NaOX overnight and expressed as fold change from control (without NaOX) (*n* = 6). **f**, De novo *PPARA* transcription assessed by the Click-iT Nascent RNA Capture assay in HepG2 cells incubated in the presence or absence of NaOX and the alkyne-modified nucleoside EU overnight. The EU-containing newly synthesized mRNAs were captured and precipitated with streptavidin magnetic beads and analysed by qRT–PCR for de novo synthesis of *PPARA* transcripts (*n* = 6). **g**, PPRE luciferase activity relative to *Renilla* luminescence in HepG2 cells transfected with PPREx3-TK-luciferase, human PPARα and *Renilla* constructs, and treated with vehicle (control), Wy 14,643 or NaOX for 24 h (*n* = 4). HepG2 cells were transfected with either GFP control (GFP) or PPARα plasmids. **h**–**j**, After 24 h, the cells were treated overnight with or without NaOX followed by analyses of *CPT1A* and *ACADM* expression relative to *GAPDH* (*n* = 4) (**h**), OCRs determined by Seahorse and normalized to protein concentrations (*n* = 4) (**i**) and neutral lipids visualized with Nile red stain and normalized to number of nuclei (*n* = 4) (**j**). **k**, HepG2 cells were treated with or without NaOX overnight followed by Seahorse analysis of OCR in the absence or presence of etomoxir (Eto; *, control versus NaOX; ^#^, control versus Control +Eto, *n* = 6). **l**, HepG2 cells were transfected with either GFP control (GFP) or GFP-tagged AGXT (AGXT) plasmids. Western blot analysis for AGXT protein abundance 48 h post-transfection (*n* = 3). **m**–**q**, After 24 h, the cells were treated with BSA-conjugated PA (200 µM) overnight followed by analysis of intracellular oxalate normalized to protein concentrations (*n* = 4) (**m**), neutral lipids visualized with Nile red stain (red) with nuclei labelled with DAPI (blue) (*n* = 3) (**n**), expression of *CPT1A* and *ACADM* relative to *GAPDH* (*n* = 4) (**o**), protein abundance of CPT1α relative to GAPDH (*n* = 5–6) (**p**) and OCR determined by Seahorse analysis and normalized to protein concentrations (*n* = 3) (**q**). For primary hepatocytes, each point represents an individual mouse. For HepG2 cells, each point represents an independent experiment that included at least two biological repetitions. All data are expressed as mean ± s.e.m. Statistical comparisons were made using two-tailed unpaired *t*-test (**b**–**e**,**m**,**o**,**p**), Mann–Whitney *U*-test (**f**), or one-way ANOVA with Tukey’s multiple comparisons test (**g**–**k**). Seahorse analysis and statistical comparisons for **i** and **q** are shown in Extended Data Fig. 7f and Extended Data Fig. 7k, respectively. All individual points and *P* values are shown. *P* < 0.05 was considered statistically significant. Scale bars, 200 µm. O.E., overexpression. Source data

**Extended Data Fig. 6:**
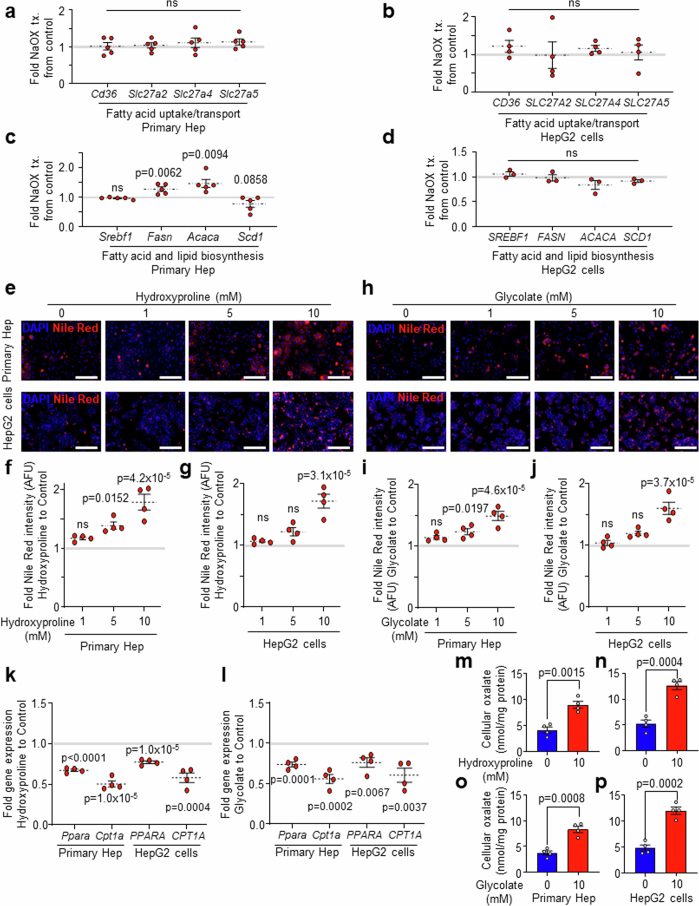
Effects of oxalate and its precursors on genes regulating lipid metabolism and lipid accumulation in hepatocytes. Primary hepatocytes isolated from mice fed a standard chow diet and HepG2 cells were treated overnight with or without sodium oxalate (NaOX, 250 μM, primary mouse hepatocytes; 500 μM, HepG2 cells). Expression of genes regulating fatty acid uptake/transport in (**a**) primary hepatocytes relative to *Gapdh* (n=5), and (**b**) in HepG2 cells relative to *GAPDH* (n=4). Expression of genes regulating fatty acid and lipid biosynthesis in (**c**) primary hepatocytes relative to *Gapdh* (n=5), and (**d**) in HepG2 cells relative to *GAPDH* (n=3). Primary hepatocytes and HepG2 cells were treated overnight with increasing concentrations of the oxalate precursor, hydroxyproline (0–10 mM). **e**, Neutral lipids were visualized with Nile Red stain (red) and nuclei were labelled with DAPI. Intensity of Nile Red staining in primary hepatocytes (n=4) (**f**) and HepG2 cells (n=4) (**g**) was normalized to number of nuclei (DAPI) and expressed as fold change. Primary hepatocytes and HepG2 cells were treated overnight with increasing concentrations of the oxalate precursor, glycolate (0–10 mM). **h**, Neutral lipids were visualized with Nile Red stain (red) and nuclei were labelled with DAPI. Intensity of Nile Red staining in primary hepatocytes (**i**) and HepG2 cells (**j**) was normalized to number of nuclei (DAPI) and expressed as fold change (n=4). Primary hepatocytes and HepG2 cells were treated overnight with 10 mM of hydroxyproline (**k**) or glycolate (**l**). The expression of *Ppara* and *Cpt1a* in primary hepatocytes was normalized to *Gapdh*, and the expression of *PPARA* and *CPT1A* in HepG2 cells was normalized to *GAPDH* (n=4). Primary hepatocytes and HepG2 cells were treated overnight with 10 mM of hydroxyproline (**m, n**) or glycolate (**o, p**), followed by analysis of intracellular oxalate normalized to protein concentrations (n=4). For primary hepatocytes, each point represents an individual mouse. For HepG2 cells, each point represents an independent experiment that included at least 2 biological repetitions. All data are expressed as mean ± SEM. Statistical comparisons were made using two-tailed unpaired t-test (**a**-**d**, **k**-**p**), or one-way ANOVA with Tukey’s multiple comparisons test (**e**-**j**). All individual points and p values are shown. A p value <0.05 was considered statistically significant. Scale bar = 200 µm. Source data

Considering that lipid loading enhances oxalate in hepatocytes and that lowering hepatic oxalate by AGXT overexpression attenuates steatohepatitis through induction of FAO pathways, we next sought to determine the effects of oxalate on lipid metabolism in hepatocytes. Treatment of primary hepatocytes and HepG2 cells with NaOX at concentrations that caused intracellular oxalate accumulation comparable with lipid loading (250 µM and 500 µM, respectively; Fig. 1q and Extended Data Fig. 3k) induced lipid accumulation as assessed by Nile red staining (Fig. 4a,b). To determine the mechanisms by which oxalate promotes lipid accumulation in hepatocytes, we assessed the expression of key genes regulating fatty acid uptake and transport, fatty acid/lipid biosynthesis and FAO by qRT–PCR. In both primary hepatocytes (Extended Data Fig. 6a) and HepG2 cells (Extended Data Fig. 6b), NaOX did not significantly alter the expression of key genes regulating fatty acid uptake and transport, including *CD36*, solute carrier family 27 member 2 (*SLC27A2*), *SLC27A4* and *SLC27A5*. With regards to fatty acid/lipid biosynthesis, primary hepatocytes treated with NaOX showed a significant increase in the key lipogenic genes, fatty acid synthase (*Fasn*) and acetyl-CoA carboxylase α (*Acaca*), with no significant differences in the expression of stearoyl-Coenzyme A desaturase 1 (*Scd1*) or sterol regulatory element binding transcription factor 1 (*Srebf1*), the master regulator of fatty acid/lipid biosynthesis^45^ (Extended Data Fig. 6c). Furthermore, HepG2 cells treated with NaOX showed no significant alterations in genes regulating fatty acid/lipid biosynthesis (Extended Data Fig. 6d). In contrast, and consistent with the changes in hepatic oxalate and the transcriptional alterations in livers from mice treated with AAV8-AGXT or AAV8-GFP, *PPARA* (Fig. 4c) and its target genes regulating FAO, *PPARGC1A*, *CPT1A* and *ACADM* (Fig. 4d), were significantly downregulated in both primary hepatocytes and HepG2 cells treated with NaOX. In line with the qRT–PCR results, NaOX significantly reduced the protein abundance of CPT1α (Fig. 4e), which is regulated by PPARα and catalyses the rate-limiting step of FAO by converting acyl-CoAs into acylcarnitines allowing their subsequent mitochondrial β-oxidation^42^. To further assess whether oxalate overproduction induces lipid accumulation in hepatocytes, we treated primary hepatocytes and HepG2 cells with increasing concentrations of the oxalate precursors, hydroxyproline and glycolate^15,19^. Treatment with oxalate precursors caused a dose-dependent increase in lipid accumulation in primary hepatocytes (Extended Data Fig. 6e–g) and HepG2 cells (Extended Data Fig. 6h–j), with a most significant effect found in cells treated with 10 mM of hydroxyproline and glycolate. Accordingly, *PPARA* and its target gene, *CPT1A*, were significantly downregulated in both primary hepatocytes (Extended Data Fig. 6k) and HepG2 cells (Extended Data Fig. 6l) that were treated with 10 mM hydroxyproline or glycolate, concomitant with a significant increase in intracellular oxalate (Extended Data Fig. 6m–o). Taken together, these studies suggest that overproduction and accumulation of oxalate in hepatocytes induce lipid accumulation by suppressing PPARα.

**Extended Data Fig. 7:**
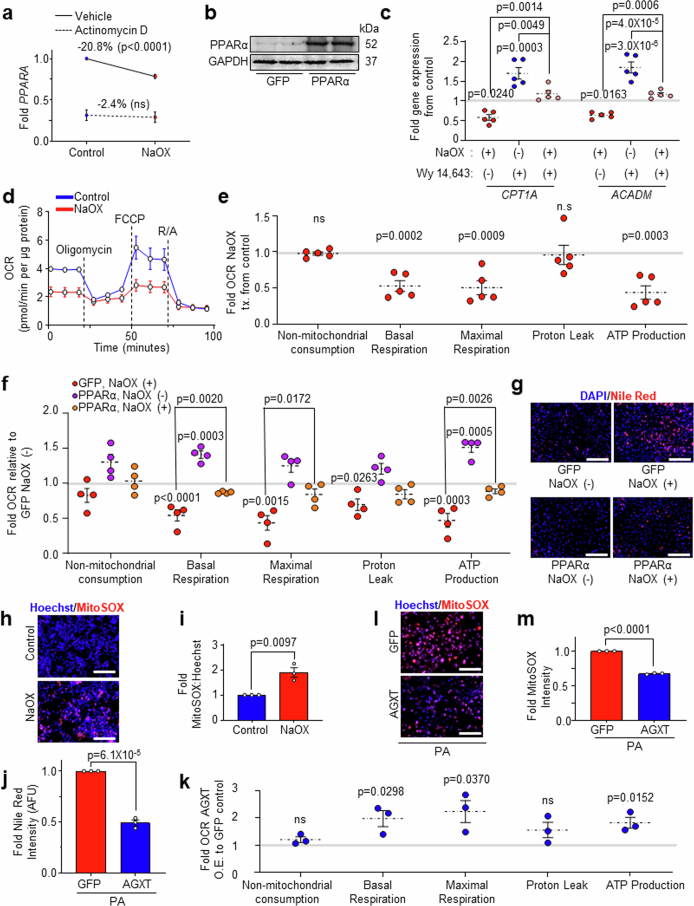
Effects of oxalate and AGXT overexpression on PPARα, mitochondrial bioenergetics and function in hepatocytes. HepG2 cells were treated overnight with or without sodium oxalate (NaOX, 500 μM). **a**, *PPARA* gene expression relative to *GAPDH* in HepG2 cells treated with (red dots) or without (control, blue dots) NaOX and with (dashed line) or without (vehicle, solid line) actinomycin D (5 μg/mL) overnight and expressed as fold change from control (without NaOX or actinomycin D) (n=4). **b**, HepG2 cells were transfected with either GFP control (GFP) or PPARα plasmids. After 48 h, the cells were lysed for Western blot analysis of PPARα relative to GAPDH (n=4). **c**, Expression of *CPT1A* and *ACADM* relative to *GAPDH* in HepG2 cells treated with or without NaOX, Wy 14,643 or vehicle control (ethanol) overnight and expressed as fold change from control (without NaOX with ethanol) (n=5). **d**, Oxygen consumption rate (OCR) determined by Seahorse analysis in HepG2 cells treated with or without NaOX overnight. OCR was normalized to protein concentrations, and (**e**) data were analysed for non-mitochondrial consumption, basal respiration, maximal respiration, proton leak, and ATP production and expressed as fold change from control cells (n=5). HepG2 cells were transfected with either GFP control (GFP) or PPARα plasmids. After 24 h, the cells were treated overnight with or without NaOX followed by analyses of (**f**) OCR by Seahorse and determination of mitochondrial consumption, basal respiration, maximal respiration, proton leak, and ATP production (n=4), and (**g**) neutral lipids visualized with Nile Red stain (red) with nuclei labelled with DAPI (blue) (n=4). **h**, Mitochondrial superoxide was visualized with MitoSOX (red) and nuclei were labelled with Hoechst (blue) in HepG2 cells treated with or without sodium oxalate (NaOX, 500 μM). **i**, Intensity of MitoSOX was normalized to number of nuclei (Hoechst) and expressed as fold change from control (n=3). HepG2 cells were transfected with either GFP control (GFP) or GFP-tagged AGXT (AGXT) plasmids. After 24 h, the cells were treated with either BSA control or BSA-conjugated palmitic acid (PA 200 µM) overnight. **j**, Neutral lipids were visualized with Nile Red stain and nuclei were labelled with DAPI. Intensity of Nile Red staining was normalized to number of nuclei (DAPI) and expressed as fold change from GFP control (n=3). **k**, Seahorse analysis of non-mitochondrial consumption, basal respiration, maximal respiration, proton leak, and ATP production expressed as fold change from GFP control (n=3). **l**, Mitochondrial superoxide was visualized with MitoSOX (red) and nuclei were labelled with Hoechst (blue). **m**, Intensity of MitoSOX was normalized to number of nuclei (Hoechst) and expressed as fold change from GFP control (n=3). Each point represents an independent experiment that included at least 2 biological repetitions. All data are expressed as mean ± SEM. Statistical comparisons were made using two-tailed unpaired t-test (**e**, **i**-**k**, **m**), two-way ANOVA with Bonferroni’s multiple comparisons test (**c**, **f**). All individual points and p values are shown. A p value <0.05 was considered statistically significant. Scale bar = 200 µm. Source data

We next sought to determine how oxalate suppresses the expression of *PPARA* and its target genes promoting FAO. To test whether the effect of oxalate on *PPARA* expression is at the transcriptional level, we treated HepG2 cells with NaOX in the absence or presence of the RNA Pol II inhibitor actinomycin D for 24 h. While NaOX significantly reduced the expression of *PPARA* in the absence of actinomycin D, RNA Pol II inhibition abolished this effect, suggesting that oxalate inhibits *PPARA* transcription (Extended Data Fig. 7a). To directly assess de novo *PPARA* transcription, we further employed a bio-orthogonal ‘click’ chemistry approach. We used the Click-iT Nascent RNA Capture assay in HepG2 cells incubated in the presence or absence of NaOX, with or without the alkyne-modified nucleoside 5-ethynyl uridine (EU), which has a tenfold higher affinity for mRNA incorporation over endogenous uridine and, thus, possesses high sensitivity via bio-orthogonal approaches. The EU-containing newly synthesized mRNAs were captured and precipitated with streptavidin magnetic beads and analysed by qRT–PCR for de novo synthesis of *PPARA* transcripts. A significant reduction in de novo *PPARA* transcription was noted in the presence of NaOX (Fig. 4f). To assess whether oxalate directly regulates PPARα transcriptional activity, we used a dual-luciferase reporter system. HepG2 cells were transfected with a PPAR response element (PPRE) reporter (PPREx3-TK-luciferase), human PPARα and *Renilla* constructs and treated with or without NaOX or with the PPARα agonist Wy 14,643 as a positive control^46^. Treatment with NaOX significantly decreased PPARα transcriptional activity (Fig. 4g), consistent with the downregulation of *PPARA* and its target genes. To determine whether the effects of oxalate are mediated by PPARα, we next restored its expression (Extended Data Fig. 7b) and activity (Wy 14,643) in NaOX-treated cells. We found that a transient overexpression of PPARα in HepG2 cells treated with NaOX reversed the suppression of the PPARα target genes, *CPT1A* and *ACADM*, compared with GFP control (Fig. 4h). Treatment with Wy 14,643 similarly reversed the downregulation of *CPT1A* and *ACADM* in NaOX-treated cells (Extended Data Fig. 7c). As PPARα induces the expression of genes regulating mitochondrial FAO that were suppressed by oxalate treatment, we next assessed the effects of oxalate on mitochondrial bioenergetics using Seahorse assays. HepG2 cells treated with NaOX showed significant reductions in basal respiration, maximal respiration and ATP production (Extended Data Fig. 7d,e). These effects were reversed by restoring PPARα (Fig. 4i and Extended Data Fig. 7f), which also abolished the lipid accumulation induced by NaOX (Fig. 4j and Extended Data Fig. 7g). We then hypothesized that these effects were due to the loss of CPT1α and the subsequent inability of fatty acids to translocate into the mitochondria for FAO. Thus, we treated HepG2 cells with or without NaOX and the CPT1α inhibitor, etomoxir^12,47^ and measured mitochondrial respiration by Seahorse. While HepG2 cells treated with NaOX were unaffected by etomoxir, inhibition of CPT1α in control cells significantly reduced the oxygen consumption rates to a level comparable with cells treated with oxalate (Fig. 4k), indicating that oxalate suppresses mitochondrial respiration in hepatocytes mainly through inhibition of FAO. Furthermore, aligned with impaired mitochondrial function, NaOX significantly enhanced mitochondrial superoxide generation (Extended Data Fig. 7h,i). Together, these findings indicate that oxalate stimulates lipid accumulation in hepatocytes by suppressing PPARα and attenuating the translocation and utilization of fatty acids in mitochondrial respiration.

Considering that overexpression of AGXT ameliorates MASH in mice through induction of FAO pathways, we next sought to determine whether these effects are mediated through the reduction of oxalate overproduction in hepatocytes. We found that a transient overexpression of AGXT (Fig. 4l) was sufficient to significantly lower PA-induced intracellular oxalate in HepG2 cells compared with GFP control (Fig. 4m), concomitant with a significant reduction in lipid accumulation assessed by Nile red (Fig. 4n and Extended Data Fig. 7j). Moreover, HepG2 cells treated with PA and overexpressing AGXT showed enhanced expression of the PPARα-target genes, *CPT1A* and *ACADM* (Fig. 4o), as well as increased CPT1α protein abundance (Fig. 4p). Furthermore, Seahorse experiments demonstrated that overexpression of AGXT in HepG2 cells treated with PA significantly enhanced basal respiration, maximal respiration and ATP production compared with GFP control (Fig. 4q and Extended Data Fig. 7k), with a suppression of mitochondrial superoxide formation (Extended Data Fig. 7l,m). Altogether, these data indicate that inhibiting the overproduction of oxalate reduces lipid accumulation in hepatocytes by inducing genes regulating FAO and mitochondrial respiration.

### AGXT overexpression blunts inflammation and fibrosis in MASH

**Fig. 5:**
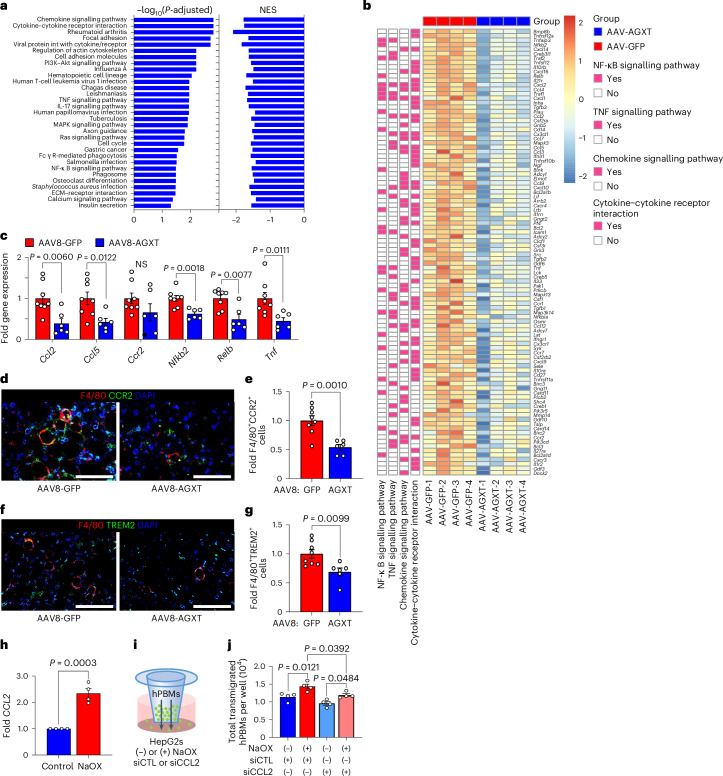
Oxalate lowering via AGXT overexpression blunts monocyte infiltration and hepatic inflammation and fibrosis in MASH. Mice were injected with AAV8-GFP or AAV8-AGXT (2 × 10^11^ viral genomes per mouse) and placed on the MASH diet for 24 weeks before end point analyses. **a**, Pathways significantly enriched in the downregulated DEGs and NES, based on KEGG pathway analysis comparing livers from mice treated with AAV8-GFP or AAV8-AGXT (*n* = 4). **b**, Heatmap of DEGs related to inflammatory pathways comparing livers from mice treated with AAV8-GFP or AAV8-AGXT (colour bar, log_2_ fold change in AAV8-AGXT versus AAV-GFP, *n* = 4). **c**, qRT–PCR validation of selected inflammation-related DEGs relative to *Gapdh* in livers from mice treated with AAV8-GFP (*n* = 8) or AAV8-AGXT (*n* = 6). **d**,**e**, Liver samples were collected from mice treated with AAV8-GFP (*n* = 8) or AAV8-AGXT (*n* = 6), stained for F4/80 (red), CCR2 (green) and DAPI (blue) to visualize nuclei (**d**), analysed for F4/80^+^ and CCR2^+^ cells and expressed as fold change from AAV8-GFP (**e**), from mice treated with AAV8-GFP (*n* = 8) or AAV8-AGXT (*n* = 6). **f**,**g**, Liver samples were collected from mice treated with AAV8-GFP (*n* = 8) or AAV8-AGXT (*n* = 6), stained for F4/80 (red), TREM2 (green) and DAPI (blue) to visualize nuclei (**f**), analysed for F4/80^+^ and TREM2^+^ cells (**g**) and expressed as fold change from AAV8-GFP, from mice treated with AAV8-GFP (*n* = 8) or AAV8-AGXT (*n* = 6). **h**, Expression of *CCL2* relative to *GAPDH* in HepG2 cells treated with or without NaOX overnight and expressed as fold change from control (*n* = 4). **i**, HepG2 cells were plated into the bottom chamber of a Transwell and transfected with siRNA against *CCL2* (siCCL2) or scrambled siRNA control (siCTL). After 24 h, cells were treated with or without NaOX overnight. Fluorescently labelled hPBMs (green) were loaded into the top chamber of the Transwell and allowed to pass through a membrane overnight. **j**, Number of transmigrated hPBMs per well (*n* = 4). All data are expressed as mean ± s.e.m. Statistical comparisons were made using two-tailed unpaired *t*-test (**c**,**e**,**g**,**h**), Mann–Whitney *U*-test (**c**) or one-way ANOVA with Tukey’s multiple comparisons test (**j**). The significance of the enriched pathways (**a**) was determined by right-tailed Fisher’s exact test followed by Benjamini–Hochberg multiple testing adjustment. All individual points and *P* values are shown. *P* < 0.05 was considered statistically significant. Scale bars, 200 µm. Source data

**Extended Data Fig. 8:**
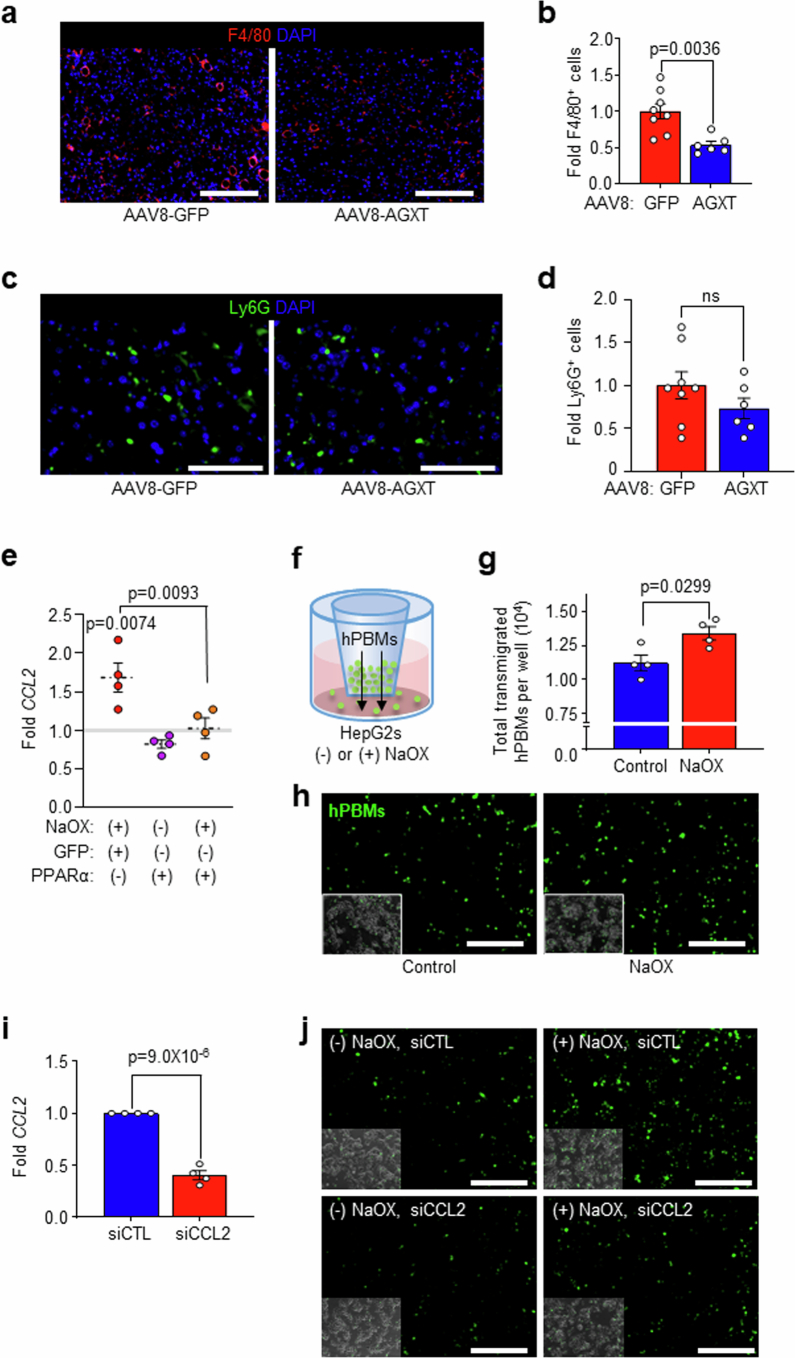
Oxalate lowering via AGXT overexpression reduces hepatic macrophages, while oxalate induces monocyte chemotaxis via CCL2. Mice were injected with AAV8-GFP or AAV8-AGXT (2x10^11^ viral genomes per mouse) and placed on the MASH diet for 24 weeks prior to end point analyses. **a**, Liver samples were collected from mice treated with AAV8-GFP (n=8) or AAV8-AGXT (n=6), stained for F4/80 (red) and DAPI (blue) to visualize nuclei, analysed for (**b**) F4/80+ cells and expressed as fold change from AAV8-GFP. **c**, Liver samples were collected from mice treated with AAV8-GFP (n=8) or AAV8-AGXT (n=6), stained for Ly6G (green) and DAPI (blue) to visualize nuclei, analysed for (**d**) Ly6G+ cells and expressed as fold change from AAV8-GFP. **e**, HepG2 cells were transfected with either GFP control (GFP) or PPARα plasmids. After 24 h, the cells were treated overnight with or without sodium oxalate (NaOX) followed by analysis of *CCL2* expression relative to *GAPDH* (n=4). **f**, HepG2 cells were plated into the bottom chamber of a transwell and treated with or without NaOX overnight. Fluorescently labelled human peripheral blood monocytes (hPBMs, green) were loaded into the top chamber of the transwell and allowed to pass through a membrane overnight. **g**, Number of transmigrated hPBMs per well (n=4). **h**, hPBMs were visualized (green) and shown with HepG2 cells (brightfield, inset) (n=4). **i**, HepG2 cells were transfected with siRNA against CCL2 (siCCL2) or scrambled siRNA control (siCTL). After 48 hours, the expression of *CCL2* relative to *GAPDH* was determined by qRT-PCR (n=4). **j**, HepG2 cells were plated into the bottom chamber of a transwell and transfected with siRNA against CCL2 (siCCL2) or scrambled siRNA control (siRNA). After 24 hours, cells were treated with or without NaOX overnight. Fluorescently labelled human peripheral blood monocytes (hPBMs, green) were loaded into the top chamber of the transwell and allowed to pass through a membrane overnight. hPBMs were visualized (green) and shown with HepG2 cells (brightfield, inset) (n=4). All data are expressed as mean ± SEM. Statistical comparisons were made using two-tailed unpaired t-test (**b**, **d**, **g**, **i**) or one-way ANOVA with Tukey’s multiple comparison’s test (**e**). All individual points and p values are shown. A p value <0.05 was considered statistically significant. Scale bars = 200 µm. Source data

Hepatic lipotoxicity promotes a proinflammatory response that facilitates leucocyte infiltration during the progression of MASH^2–4^. Mice fed the MASH diet and treated with AAV8-AGXT demonstrated a significant reduction in lobular inflammation compared with mice treated with AAV8-GFP (Fig. 2j,k). Accordingly, KEGG pathway analysis based on liver RNA sequencing revealed a significant downregulation of multiple pathways involved in inflammation including chemokine signalling, cytokine–cytokine receptor interaction, as well as nuclear factor (NF)-κB and tumour necrosis factor (TNF) signalling pathways (Fig. 5a). RNA sequencing analysis (Fig. 5b), validated by qRT–PCR analyses (Fig. 5c), indicated significant reductions in key genes of the NF-κB and TNF signalling pathways in livers from mice overexpressing AGXT, including *Nfkb2*, *Relb* and *Tnf* as well as chemokine (C–C motif) ligand 2 (*Ccl2*) and *Ccl5* that are known as drivers and therapeutic targets in MASH together with *Ccr2* that regulate the recruitment of monocyte-derived cells in MASH^48^. Accordingly, immunofluorescence for F4/80 revealed a significant reduction in total hepatic macrophages in livers from mice overexpressing AGXT (Extended Data Fig. 8a,b). Utilizing immunofluorescence analyses, we further assessed alterations in immune cell subsets in livers from mice treated with AAV8-AGXT or AAV8-GFP. While no significant changes in Ly6G^+^ neutrophils were noted (Extended Data Fig. 8c,d), livers from mice treated with AAV8-AGXT had significantly lower levels of F4/80^+^CCR2^+^ macrophages (Fig. 5d,e), and F4/80^+^ TREM2^+^ macrophages (Fig. 5f,g), distinct subsets of recruited monocyte-derived macrophages that infiltrate the liver and form crown-like structures surrounding hepatocytes with large lipid droplets^49^. As these results indicate that lowering oxalate blunts monocyte recruitment to the liver, we next assessed a potential chemotactic effect of oxalate. To this end, HepG2 cells were plated in the lower well of a Transwell, and the transmigration of fluorescently labelled human primary blood monocytes (hPBMs) was measured in cells treated with or without NaOX. Consistent with our in vivo findings, and with a PPARɑ dependent upregulation of *CCL2* in HepG2 cells treated with NaOX (Fig. 5h and Extended Data Fig. 8e), there was a significant elevation of monocytes transmigrating toward the bottom well containing HepG2 cells treated with NaOX compared with control cells (Extended Data Fig. 8f–h). To test whether CCL2 is mediating the chemotactic effects of oxalate, we knocked down *CCL2* in HepG2 cells using siRNA (siCCL2) as confirmed by qRT–PCR (Extended Data Fig. 8i). HepG2 cells treated with siCCL2 or scrambled siRNA control (siCTL) were plated in the lower well of the Transwell, and the transmigration of fluorescently labelled hPBMs was measured in cells treated with or without NaOX (Fig. 5i). In HepG2 cells treated with siCTL, NaOX caused a significant increase in monocyte transmigration and this effect was significantly attenuated by *CCL2* knockdown (Fig. 5j and Extended Data Fig. 8j). Together, these data indicate that oxalate promotes monocyte chemotaxis by upregulating hepatocyte CCL2 and that lowering oxalate overproduction in MASH through AGXT overexpression blunts monocyte infiltration and hepatic inflammation.

**Fig. 6:**
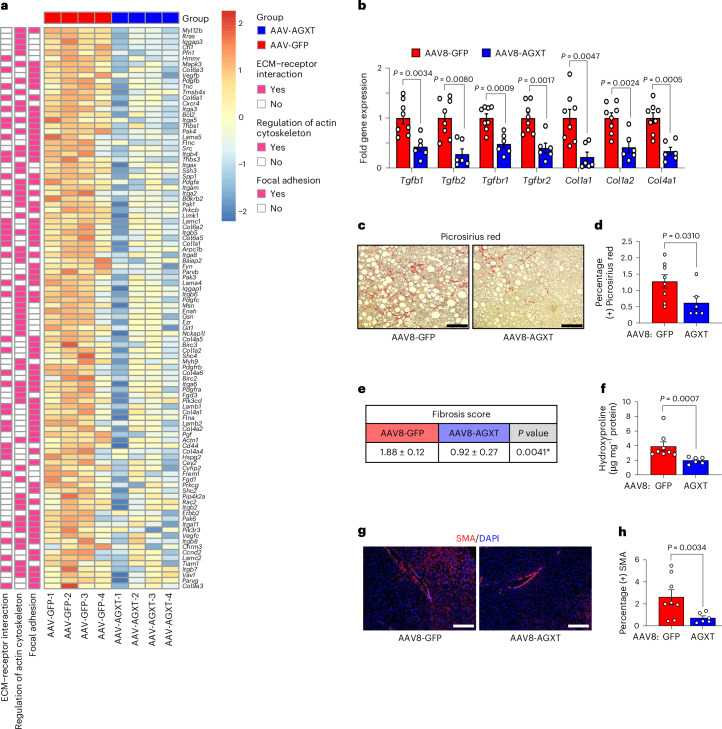
Oxalate lowering via AGXT overexpression decreases hepatic fibrosis in MASH. Mice were injected with AAV8-GFP or AAV8-AGXT (2 × 10^11^ viral genomes per mouse) and placed on the MASH diet for 24 weeks before end point analyses. **a**, Heatmap of DEGs related to fibrosis pathways comparing livers from mice treated with AAV8-GFP or AAV8-AGXT (colour bar, log_2_ fold change in AAV8-AGXT versus AAV-GFP, *n* = 4). **b**, qRT–PCR validation of selected fibrosis-related DEGs relative to *Gapdh* in livers from mice treated with AAV8-GFP (*n* = 8) or AAV8-AGXT (*n* = 6). **c**, Liver samples were collected from mice treated with AAV8-GFP (*n* = 8) or AAV8-AGXT (*n* = 6) and stained with Picrosirius red (red). **d**, Percent-positive Picrosirius red area from mice treated with AAV8-GFP (*n* = 8) or AAV8-AGXT (*n* = 6). **e**, Liver sections from mice treated with AAV8-GFP (*n* = 8) or AAV8-AGXT (*n* = 6) were scored for fibrosis. **f**, Hydroxyproline contents normalized to protein concentrations in liver samples from mice treated with AAV8-GFP (*n* = 8) or AAV8-AGXT (*n* = 6). **g**, Liver samples were collected from mice treated with AAV8-GFP (n = 8) or AAV8-AGXT (*n* = 6) and stained with α-SMA (red) and DAPI (blue). **h**, Percent-positive α-SMA area from mice treated with AAV8-GFP (*n* = 8) or AAV8-AGXT (*n* = 6). All data are expressed as mean ± s.e.m. Statistical comparisons were made using two-tailed unpaired *t*-test (**b**,**e**,**h**) or Mann–Whitney *U*-test (**b**,**d**,**f**). All individual points and *P* values are shown. *P* < 0.05 was considered statistically significant. Scale bars, 200 µm. Source data

**Extended Data Fig. 9:**
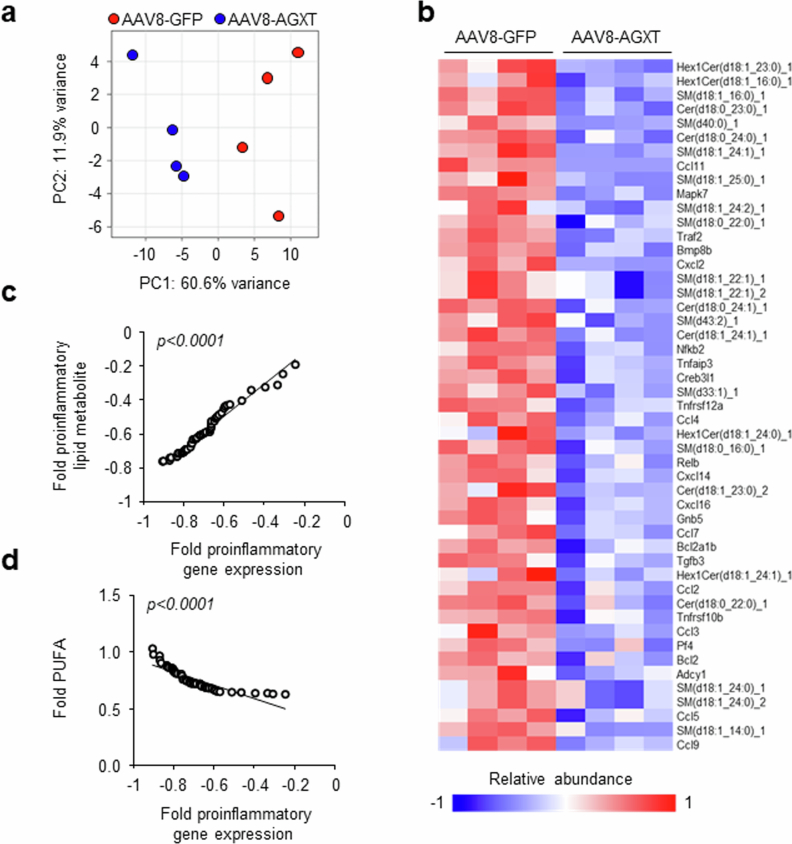
Integration of the hepatic lipidome and transcriptome in mice overexpressing AGXT. Male C57BL/6J mice were injected with AAV8-TBG-GFP or AAV8-TBG-AGXT (2x10^11^ viral genomes per mouse) and placed on the MASH diet for 24 weeks prior to end point analyses. **a**, Principal component analysis was performed based on untargeted lipidomics integrated with RNA-sequencing of livers from the same mice treated with AAV8-GFP or AAV8-AGXT (n=4). **b**, Heatmap of proinflammatory and lipotoxic sphingolipid metabolites (sphingomyelins, ceramides, and hexosylceramides) integrated with DEGs related to inflammatory and fibrotic pathways comparing livers from mice treated with AAV8-GFP or AAV8-AGXT (n=4, scale bar: relative abundance). Spearman’s correlations were calculated between the expression of proinflammatory/fibrotic genes and the abundance of (**c**) proinflammatory sphingolipid metabolites, and (**d**) PUFAs in triglycerides in livers from mice treated with AAV8-GFP or AAV8-AGXT. A p value <0.05 was considered statistically significant. Source data

Hepatic fibrosis is the main determinant of liver-related events and mortality in MASH^50^. In addition to the suppression of proinflammatory pathways in mice overexpressing AGXT in hepatocytes during MASH, the KEGG pathway analysis revealed a significant downregulation of pathways associated with hepatic fibrosis including focal adhesion signalling, regulation of actin cytoskeleton and extracellular matrix (ECM)–receptor interactions (Fig. 5a). RNA sequencing analysis (Fig. 6a), validated by qRT–PCR analyses (Fig. 6b), indicated a significant downregulation of genes within the transforming growth factor (TGF)β signalling pathway, a main regulator of hepatic fibrosis^51^ (*Tgfb1*, *Tgfb2*, *Tgfbr1* and *Tgfbr2*) as well as key genes related to ECM remodelling (*collagen*, *type I*, *α-1* (*Col1a1*), *Col1a2* and *Col4a1*) in mice overexpressing AGXT. As these transcriptional alterations suggest a reduction in hepatic fibrosis in response to lowering oxalate via AGXT overexpression, we next evaluated hepatic fibrosis through histopathological and immunofluorescence analyses coupled with biochemical verification. Histopathological analysis based on Picrosirius red staining revealed a significant reduction in collagen accumulation (Fig. 6c,d) and decreased fibrosis scores (Fig. 6e) in mice overexpressing AGXT compared with GFP control. These findings were confirmed biochemically through the measurement of liver hydroxyproline content, which was significantly lower in mice overexpressing AGXT compared with GFP control (Fig. 6f). Concomitant with these results, immunofluorescence for α-smooth muscle actin (SMA) indicated reduced presence of hepatic stellate cells, the main cells driving fibrogenesis^3,51^, in livers from mice treated with AAV8-AGXT (Fig. 6g,h). Last, we sought to determine the link between altered lipid metabolism due to AGXT overexpression, hepatic inflammation and fibrosis in MASH. To this end, we integrated the untargeted lipidomics with RNA sequencing of livers from the same mice that were treated with AAV8-AGXT or AAV8-GFP. PCA revealed that the integrated lipidome and transcriptome of livers from mice treated with AAV8-GFP was distinct from those treated with AAV8-AGXT (Extended Data Fig. 9a). Of note, the reduced hepatic expression of known proinflammatory and profibrotic genes (for example, *Nfkb2*, *Relb*, *Ccl2*, *Ccl5*, *Tgfb3* and *Bmp8b*) in livers from mice overexpressing AGXT aligned with lower abundance of the proinflammatory and lipotoxic sphingolipid metabolites (sphingomyelins, ceramides and hexosylceramides; Extended Data Fig. 9b). Accordingly, the expression of proinflammatory/fibrotic genes significantly and positively correlated with the abundance of sphingolipid metabolites (Extended Data Fig. 9c), but negatively correlated with the abundance of PUFAs (Extended Data Fig. 9d). Taken together, these data demonstrate that lowering oxalate via AGXT overexpression in MASH protects against hepatic inflammation and fibrosis in association with reduced lipotoxicity.

### Pharmacological inhibition of oxalate production lowers MASH

**Fig. 7:**
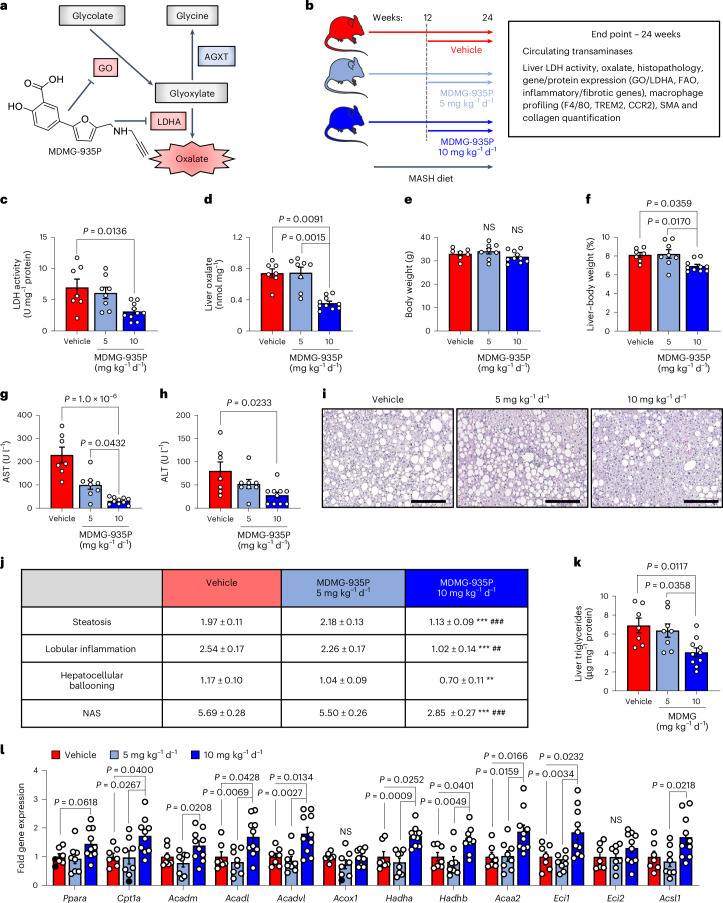
Pharmacological targeting of hepatic oxalate overproduction ameliorates established MASH. **a**, Schema of glyoxylate/oxalate metabolism, including the chemical structure of MDMG-935P and its inhibitory effects. **b**, Male C57BL/6J mice were fed the MASH diet for 12 weeks, then orally administered vehicle (*n* = 7), 5 mg kg^−1^ d^−1^ (*n* = 8) or 10 mg kg^−1^ d^−1^ (*n* = 10) of MDMG-935P for an additional 12 weeks on the MASH diet before end point analyses. **c**,**d**, Liver LDH activity (**c**) and oxalate concentrations normalized to tissue weight (**d**) in liver samples from mice administered vehicle (*n* = 7), 5 mg kg^−1^ d^−1^ (*n* = 8) or 10 mg kg^−1^ d^−1^ (*n* = 10) of MDMG-935P. **e**–**j**, Body weight (**e**) and liver-to-body weight ratios (**f**) in mice administered vehicle (*n* = 7), 5 mg kg^−1^ d^−1^ (*n* = 8) or 10 mg kg^−1^ d^−1^ (*n* = 10) of MDMG-935P. Plasma samples were analysed for AST (**g**) and ALT (**h**) in mice administered vehicle (*n* = 7), 5 mg kg^−1^ d^−1^ (*n* = 8) or 10 mg kg^−1^ d^−1^ (*n* = 10) of MDMG-935P. Liver samples were collected from mice administered vehicle (*n* = 7), 5 mg kg^−1^ d^−1^ (*n* = 8) or 10 mg kg^−1^ d^−1^ (*n* = 10) of MDMG-935P, stained with H&E (**i**) and scored for steatosis, lobular inflammation, hepatocellular ballooning and NAS (**j**). **P < 0.01, ***P < 0.001 versus vehicle; ^##^*P* < 0.01, ^###^*P* < 0.001 versus 5 mg kg^−1^ d^−1^ of MDMG-935P. **k**, Liver triglycerides normalized to protein concentrations from mice treated with vehicle (*n* = 7), 5 mg kg^−1^ d^−1^ (*n* = 8) or 10 mg kg^−1^ d^−1^ (*n* = 10) of MDMG-935P. **l**, Liver samples were collected from mice treated with vehicle (*n* = 7), 5 mg kg^−1^ d^−1^ (*n* = 8) or 10 mg kg^−1^ d^−1^ (*n* = 10) of MDMG-935P and FAO-related genes relative to *Gapdh* were assessed by qRT–PCR. All data are expressed as mean ± s.e.m. Statistical comparisons were made using one-way ANOVA with Tukey’s multiple comparisons test (**c**–**g**,**j**–**l**) or Kruskal–Wallis with Dunn’s multiple comparisons test (**h**,**j**,**l**). *P* < 0.05 was considered statistically significant. Scale bars, 200 µm. Parts of **b** were drawn by using pictures from Servier Medical Art. Servier Medical Art by Servier is licensed under a Creative Commons Attribution 3.0 Unported License at https://creativecommons.org/licenses/by/3.0/. Source data

**Extended Data Fig. 10:**
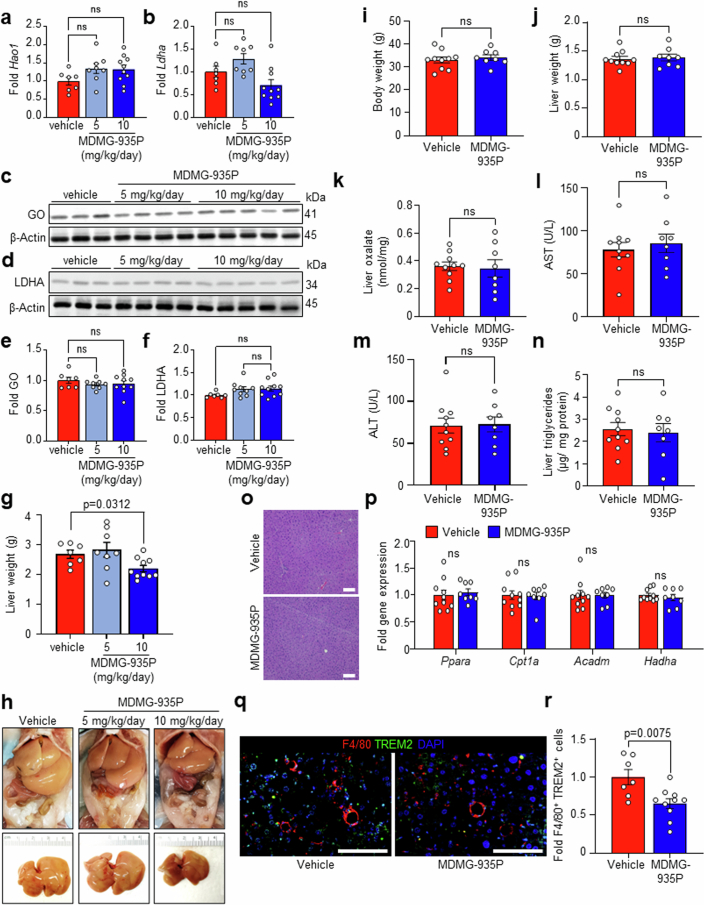
Effects of MDMG-935P treatment on MASH or standard chow diet. Male C57BL/6J mice were fed the MASH diet for 12 weeks, then orally administered vehicle (n=7), 5 mg/kg/day (n=8) or 10 mg/kg/day (n=10) of MDMG-935P for an additional 12 weeks on the MASH diet prior to end point analyses. Liver samples were collected from the treated mice and the expression of (**a**) *Hao1*, and (**b**) *Ldha* relative to *Gapdh* was assessed by qRT-PCR. Protein abundance of GO (**c, e**), and LDHA (**d, f**) relative to β-Actin in liver samples from mice treated with vehicle, 5 mg/kg/day or 10 mg/kg/day of MDMG-935P. **g**, Liver weight, and (**h**) representative gross appearance of abdominal cavities and livers from mice treated with vehicle, 5 mg/kg/day or 10 mg/kg/day of MDMG-935P. C57BL/6J mice were fed a standard chow diet for 12 weeks, then orally administered vehicle (n=10), or 10 mg/kg/day of MDMG-935P (n=8) for an additional 12 weeks on the standard chow diet prior to end point analyses. **i**, body weight, (**j**) liver weight, and (**k**) liver oxalate in mice treated with vehicle (n=10) or 10 mg/kg/day of MDMG-935P (n=8). **l**, Plasma samples were analysed for aspartate transaminase (AST), and (**m**) alanine transaminase (ALT) in mice treated with vehicle (n=10) or 10 mg/kg/day of MDMG-935P (n=8). **n**, Liver triglycerides normalized to protein concentrations in livers from mice treated with vehicle (n=10) or 10 mg/kg/day of MDMG-935P (n=8). **o**, Livers from mice treated with vehicle (n=10) or 10 mg/kg/day of MDMG-935P (n=8) were sectioned and stained with H&E. **p**, Liver samples were collected from mice treated with vehicle (n=10), or 10 mg/kg/day of MDMG-935P (n=8), and FAO-related genes relative to *Gapdh* were assessed by qRT-PCR. **q**, Male C57BL/6J mice were fed the MASH diet for 12 weeks, then orally administered vehicle or 10 mg/kg/day of MDMG-935P for an additional 12 weeks on the MASH diet prior to end point analyses. Liver samples were collected from mice treated with vehicle (n=7), or 10 mg/kg/day (n=10) of MDMG-935P, stained for F4/80 (red), TREM2 (green), and DAPI (blue) to visualize nuclei, analysed for (**r**) F4/80+ and TREM2+ cells and expressed as fold change from vehicle. The samples were derived from the same experiment and the blots were processed in parallel for (**c**, **d**, **e**, **f**). All data are expressed as mean ± SEM. Statistical comparisons were made using one-way ANOVA with Tukey’s multiple comparisons test (**a**, **b**, **e**, **f**, **g**), or two-tailed unpaired t-test (**i**-**n**, **p**, **r**). All individual points and p values are shown. A p value <0.05 was considered statistically significant. Scale bars = 200 μm. Source data

Given that AGXT overexpression lowers hepatic oxalate overproduction and prevents MASH, we next sought to determine (1) whether these protective effects are mediated by AGXT or through an oxalate-lowering effect; and (2) to assess the therapeutic value of pharmacologically targeting hepatic oxalate overproduction in established MASH. While GO converts glycolate to glyoxylate, which is cleared by AGXT, LDHA catalyses the oxidation of glyoxylate to form oxalate^19^ (Fig. 1a). Considering the enhanced activity of LDHA found in livers from patients and mice with MASH (Fig. 1f,m), together with the recent advances in targeting GO or LDHA for the treatment of primary hyperoxaluria^52,53^, we utilized our recently developed salicylic acid derivative, MDMG-935P (Fig. 7a), which potently decreases oxalate production by inhibiting GO and LDHA^54^. To assess the therapeutic potential of oxalate lowering, we devised an experimental approach to test MDMG-935P in mice with established MASH. Mice were fed the MASH diet for 12 weeks, then orally administered either vehicle, 5 mg kg^−1^ or 10 mg kg^−1^ of MDMG-935P daily for an additional 12 weeks on the MASH diet (Fig. 7b). As previous studies demonstrated that a daily dose of 20 mg kg^−1^ of MDMG-935P for only 5 days effectively reduced oxalate in primary hyperoxaluric mice^54^, we chose to administer up to 10 mg kg^−1^ d^−1^ to account for the long duration of the treatments. At the end point, we found that 10 mg kg^−1^ d^−1^ of MDMG-935P significantly reduced hepatic LDH activity (Fig. 7c), and oxalate levels (Fig. 7d) compared with vehicle, while 5 mg kg^−1^ d^−1^ had no significant effect. The mRNA (Extended Data Fig. 10a,b) and protein (Extended Data Fig. 10c–f) levels of GO and LDHA in the liver were unaltered by MDMG-P35P treatment. While the mice showed no significant alterations in body weight (Fig. 7e), treatment with 10 mg kg^−1^ d^−1^ of MDMG-935P significantly reduced the liver weight (Extended Data Fig. 10g) and liver-to-body weight ratio (Fig. 7f) compared to mice treated with vehicle. Furthermore, circulating markers of liver injury, AST (Fig. 7g) and ALT (Fig. 7h), were significantly decreased in mice treated with 10 mg kg^−1^ d^−1^ of MDMG-935P. While gross morphology of the abdominal cavity showed no obvious differences in adiposity in all groups (Extended Data Fig. 10h), histopathological analysis of the livers (Fig. 7i) revealed significant reductions in steatosis, lobular inflammation, hepatocellular ballooning and overall NAS in mice treated with 10 mg kg^−1^ d^−1^ of MDMG-935P (Fig. 7j). Consistent with the reduction in steatosis scoring, biochemical analysis of liver lysates confirmed a significant reduction in hepatic triglycerides in mice treated with 10 mg kg^−1^ d^−1^ of MDMG-935P (Fig. 7k). In line with the findings in mice and cells overexpressing AGXT, and the inhibitory effects of oxalate on PPARα-mediated FAO, genes regulating FAO were significantly upregulated in livers from mice treated with 10 mg kg^−1^ d^−1^ of MDMG-935P including *Cpt1a*, *Acadl*, *Acadvl*, *Hadha*, *Hadhb* and *Acaa2* (Fig. 7l). Notably, in mice fed the standard chow diet, treatment with 10 mg kg^−1^ d^−1^ MDMG-935P for 12 weeks had no significant effects on body (Extended Data Fig. 10i) and liver (Extended Data Fig. 10j) weights, liver oxalate (Extended Data Fig. 10k), circulating transaminases (Extended Data Fig. 10l,m), hepatic steatosis (Extended Data Fig. 10n,o) or the expression of genes regulating FAO (Extended Data Fig. 10p). Altogether, these data indicate that targeting hepatic oxalate overproduction in MASH by inhibiting GO and LDHA can be therapeutically utilized to enhance FAO and lower hepatic steatosis.

### Inhibiting oxalate formation curbs inflammation and fibrosis

**Fig. 8:**
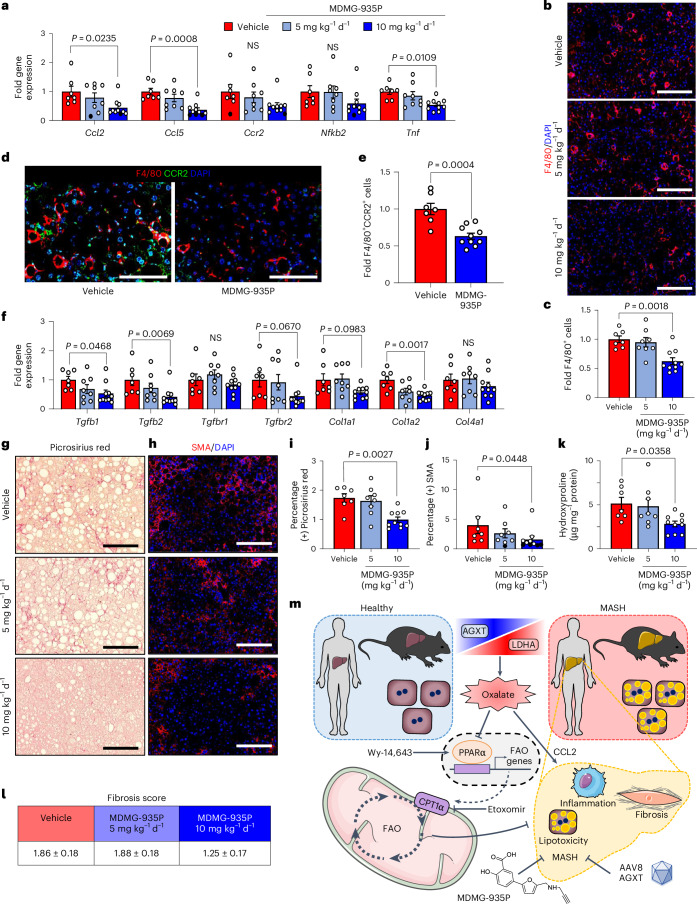
Pharmacological targeting of hepatic oxalate overproduction reduces hepatic inflammation and fibrosis. Male C57BL/6J mice were fed the MASH diet for 12 weeks, then orally administered vehicle (*n* = 7), 5 mg kg^−1^ d^−1^ (*n* = 8) or 10 mg kg^−1^ d^−1^ (*n* = 10) of MDMG-935P for an additional 12 weeks on the MASH diet before end point analyses. **a**, Liver samples were collected from the treated mice, and inflammation-related genes were assessed by qRT–PCR relative to *Gapdh* from mice treated with vehicle (*n* = 7), 5 mg kg^−1^ d^−1^ (*n* = 8) or 10 mg kg^−1^ d^−1^ (*n* = 10) MDMG-935P. **b**,**c**, Liver samples were collected from the mice treated with vehicle (*n* = 7), 5 mg kg^−1^ d^−1^ (*n* = 8) or 10 mg kg^−1^ d^−1^ (*n* = 10) MDMG-935P, stained with F4/80 (red) and DAPI (blue) to visualize nuclei (**b**), analysed for F4/80^+^ cells and expressed (**c**) as fold change from vehicle. **d**,**e**, Liver samples were collected from mice treated with vehicle (*n* = 7) or 10 mg kg^−1^ d^−1^ (*n* = 10) of MDMG-935P, stained for F4/80 (red), CCR2 (green) and DAPI (blue) (**d**), analysed for F4/80^+^ and CCR2^+^ cells (**e**) and expressed as fold change from vehicle. **f**, Liver samples were collected from the treated mice and fibrosis-related genes were assessed by qRT–PCR relative to *Gapdh* from mice treated with vehicle (*n* = 7), 5 mg kg^−1^ d^−1^ (*n* = 8) or 10 mg kg^−1^ d^−1^ (*n* = 10) MDMG-935P. **g**,**i**, Liver sections were stained with Picrosirius red (red) (**g**) and quantified for percent-positive Picrosirius red area (**i**) from mice treated with vehicle (*n* = 7), 5 mg kg^−1^ d^−1^ (*n* = 8) or 10 mg kg^−1^ d^−1^ (*n* = 10) MDMG-935P. **h**,**j**, Liver sections were stained with α-SMA and DAPI (blue) (**h**) and analysed for percent-positive α-SMA area (**j**) from mice treated with vehicle (*n* = 7), 5 mg kg^−1^ d^−1^ (*n* = 8) or 10 mg kg^−1^ d^−1^ (*n* = 10) MDMG-935P. **k**, Hydroxyproline contents normalized to protein concentration in liver samples from mice treated with vehicle (*n* = 7), 5 mg kg^−1^ d^−1^ (*n* = 8) or 10 mg kg^−1^ d^−1^ (*n* = 10) MDMG-935P. **l**, Liver sections were scored for fibrosis based on Picrosirius red staining. **m**, Schematic summary of oxalate overproduction in MASH, the effects of oxalate on MASH, and inhibition of oxalate production by either AAV-AGXT overexpression or pharmacological targeting using MDMG-935P. All data are expressed as mean ± s.e.m. Statistical comparisons were made using one-way ANOVA with Tukey’s multiple comparisons test (**a**,**c**,**i**,**k**,**l**), Kruskal–Wallis with Dunn’s multiple comparisons test (**a**,**f**,**j**), or two-tailed unpaired *t*-test (**e**). All individual points and *P* values are shown. *P* < 0.05 was considered statistically significant. Scale bars, 200 μm. Parts of **m** were drawn by using pictures from Servier Medical Art. Servier Medical Art by Servier is licensed under a Creative Commons Attribution 3.0 Unported License at https://creativecommons.org/licenses/by/3.0/. Source data

Considering that AGXT overexpression attenuated hepatic inflammation and fibrosis in MASH, we next sought to determine whether the pharmacological targeting of hepatic oxalate overproduction in mice with established MASH is sufficient to suppress proinflammatory and profibrotic responses. Consistent with the findings from mice overexpressing AGXT, mice administered 10 mg kg^−1^ d^−1^, but not 5 mg kg^−1^ d^−1^ of MDMG-935P, exhibited significant reductions in the expression of key chemokines and cytokines driving the proinflammatory response in MASH including *Ccl2*, *Ccl5* and *Tnf* (Fig. 8a). Accordingly, immunofluorescence for F4/80 revealed a significant reduction in hepatic macrophages in livers from mice treated with 10 mg kg^−1^ d^−1^ of MDMG-935P compared with vehicle (Fig. 8b,c). Immunofluorescence analyses of macrophage subsets revealed a significant reduction in recruited monocyte-derived F4/80^+^CCR2^+^ macrophages (Fig. 8d,e) and F4/80^+^TREM2^+^ macrophages (Extended Data Fig. 10q,r) in livers from mice treated with 10 mg kg^−1^ d^−1^ of MDMG-935P. Furthermore, treatment with MDMG-935P at 10 mg kg^−1^ d^−1^ had potent anti-fibrotic effects and significantly lowered the expression of key genes within the TGFβ signalling and ECM remodelling pathways, including *Tgfb1*, *Tgfb2* and *Col1a2* (Fig. 8f). These findings were aligned with histopathological analyses coupled with biochemical assessment of fibrosis, which revealed significant reductions in Picrosirius red staining (Fig. 8g,i), SMA immunofluorescence (Fig. 8h,j) and hepatic hydroxyproline content (Fig. 8k) in mice treated with 10 mg kg^−1^ d^−1^ of MDMG-935P. Finally, assessment of fibrosis scores showed similar levels between vehicle and 5 mg kg^−1^ d^−1^ and a downward trend with 10 mg kg^−1^ d^−1^ of MDMG-935P, although not reaching statistical significance (*P* = 0.0798; Fig. 8l). Overall, these data indicate that pharmacologically inhibiting oxalate overproduction in mice with established MASH suppresses oxalate-induced proinflammatory responses and hepatic fibrosis.

## Discussion

The findings herein were obtained from studies in multiple human cohorts, MASH mouse models and hepatocellular systems combined with genetic and pharmacological manipulation of glyoxylate/oxalate metabolism together with transcriptomics, lipidomics, as well as molecular and functional analyses. Through this comprehensive approach, we uncovered molecular and metabolic mechanisms by which (1) oxalate is overproduced in hepatocytes during MASH; (2) oxalate overload promotes MASH; and (3) lowering oxalate ameliorates MASH across all aspects of the disease. Our findings unveil the overproduction of oxalate due to suppressed AGXT and activated LDHA, both in humans and mice with MASH. In turn, oxalate overload suppresses PPARα transcription and the expression of its target genes controlling FAO (CPT1α), leading to impaired mitochondrial fatty acid utilization and lipotoxicity, as well as the upregulation of MASH-promoting chemokines (CCL2), monocyte chemotaxis, hepatic inflammation and fibrosis. Notably, lowering hepatic oxalate in MASH by genetic (hepatocyte-specific AGXT overexpression by AAV8-AGXT) and pharmacological (GO and LDHA inhibition by MDMG-935P) targeting of oxalate overproduction ameliorates hepatic steatosis, inflammation and fibrosis through induction of PPARα-driven FAO and suppression of monocyte chemotaxis, NF-κB and TGFβ targets (Fig. 8m).

Because dysregulated oxalate metabolism is known to cause renal and cardiovascular diseases^22–26^, previous studies have focused on the deleterious effects of oxalate on the kidneys and renal cells as well as monocytes and macrophages^22–25,55^. However, primary hyperoxaluria and AGXT dysfunction have recently been associated with liver disease and MASLD^12,15,20,21^. A recent study comparing clinical characteristics of patients with or without primary hyperoxaluria reported significantly higher rates of liver disease in those with primary hyperoxaluria^20^. Moreover, a systematic analysis of liver tissues from patients with primary hyperoxaluria revealed high rates of chronic liver disease, hepatic inflammation and fibrosis in patients with AGXT dysfunction^21^. Previous studies also reported that *AGXT* transcript is downregulated in livers from patients and mice with obesity, MASLD or MASH^12,15–17^. Nevertheless, the hepatic regulation of oxalate metabolism, the levels of oxalate in patients and mice with MASH and the effects of oxalate on the liver and hepatocytes, the primary cells responsible for its formation^19^, have not been systematically studied in the context of MASH yet. Herein, we thoroughly assessed the expression of the major regulators of glyoxylate metabolism and oxalate formation in multiple cohorts, including liver samples from transplantation donors, patients with or without histologically confirmed MASH and different mouse models of MASH. Our studies revealed that AGXT was significantly downregulated in all the human and mouse MASH cohorts tested, supporting previous reports from our group and others^12,15–17^. The expression of *AGXT* significantly and inversely correlated with the severity of hepatic steatosis in our cohort of liver transplantation donors. Moreover, our meta-analysis based on transcriptomics of livers from patients with or without MASH revealed that among all the glyoxylate metabolic genes, only lower expression of *AGXT* is significantly associated with MASH. Similarly, in our cohort of liver specimens from patients with histologically confirmed end-stage MASH, among all the regulators of glyoxylate metabolism or oxalate formation, only AGXT was significantly suppressed as compared to healthy donors. Although altered expression of LDHA was not associated with MASH in any of the human cohorts, hepatic LDH activity was significantly enhanced in patients with MASH. Taken together, the current comprehensive studies, together with previous reports^12,15–17^, establish the suppression of AGXT and enhanced activity of LDHA as the underlying mechanisms for the overproduction of oxalate in MASH.

In line with the human findings above, suppressed AGXT and increased hepatic oxalate were also found in our MASH mouse model that closely mimics the human disease, including the presence of hepatic fibrosis^12,32–34^. Using transcriptomics, histopathological and western blot analyses, the liver fibrosis markers previously found after 24 weeks on this diet include a significant upregulation of pathways/genes related to fibrogenesis and ECM remodelling (*Col1a1*, *Col1a2*, *Col3a1*, *Col4a1*, *Col4a2*, *Timp1* and *Serpine1*), TGFβ (*Tgfb1*, *Tgfb2*, *Tgfb3*, *Tgfbr1* and *Tgfbr2*) and SMAD signalling (SMAD2 Ser^465/467^ phosphorylation), as well as perisinusoidal and portal fibrosis^12,32–34^. Similar markers of liver fibrosis were also found in the current study where hypermethylation at the *Agxt* promoter and first exon accounted for the suppression of AGXT in support of a recent study reporting hypermethylation in similar regions in steatotic hepatocytes^15^. Notably, we found that AGXT was downregulated in both female and male mice with MASH induced by another dietary model^35^, indicating that this effect is independent of diet or sex. Notably, the suppression of AGXT and increased hepatic oxalate found in our cohort of liver specimens from patients with end-stage MASH were also independent of potential confounding factors, including race, age or sex. Both in humans and mice, AGXT expression was inversely associated, whereas hepatic oxalate levels were positively associated, with histopathological indices of MASH severity. These correlations were more significant in the mouse model compared with the human cohort, likely due to the advanced disease stage of the human liver specimens and the hepatic fat loss commonly seen in advanced fibrotic MASH^56^. While hepatic LDH activity was significantly enhanced in both humans and mice with MASH, the mRNA and protein levels of LDHA were increased in livers from mice, but not humans, with MASH. These findings are consistent with a recent study reporting enhanced LDHA expression in steatotic livers from mice, but not in human steatotic hepatocytes^15^. While the species differences in LDHA expression in MASH warrant further investigation, the findings of the current and previous studies^12,15–17^ provide a rationale for the therapeutic targeting of glyoxylate detoxification or oxalate formation in MASH by overexpressing AGXT or inhibiting LDHA.

In addition to the accumulating evidence above establishing AGXT suppression in MASH, our recent studies uncovered a causative role for the loss of AGXT in MASH. As we reported^12^, mice deficient in AGXT demonstrate accelerated diet-induced MASH. Yet, whether these effects are mediated by increased liver oxalate and the therapeutic value of lowering hepatic oxalate overproduction in MASH were unknown. Here, we addressed those questions using a multidisciplinary approach combining genetic and pharmacological manipulation of glyoxylate metabolism and oxalate formation with molecular and metabolic in vivo and in vitro studies. Our genetic approach was based on overexpressing AGXT specifically in hepatocytes using AAV8 driven by the TBG promoter^24,57^, followed by 24 weeks on the MASH diet. Hepatic gene overexpression using AAV vectors is commonly used for prolonged studies, even longer than 24 weeks^58,59^. Indeed, using western blot and immunofluorescence, we confirmed that treatment with AAV8-AGXT or AAV8-GFP resulted in efficient and hepatic-specific overexpression after 24 weeks on the MASH diet. These studies revealed that AGXT overexpression in hepatocytes significantly lowers hepatic oxalate and prevents diet-induced MASH and liver injury, independent of changes in body weight or adiposity. To address the therapeutic potential of targeting hepatic oxalate overproduction for the treatment of established MASH, we utilized our approach for inhibiting GO and LDHA using the salicylic acid derivative MDMG-935P^54^. Notably, we found that oral administration of MDMG-935P to mice with established MASH significantly reduced hepatic oxalate overproduction, leading to a marked reduction in steatohepatitis, hepatic injury and fibrosis. Unlike the mice with diet-induced MASH that demonstrated hepatic oxalate overproduction due to suppressed AGXT and enhanced LDHA activity, overexpression of AGXT or treatment with MDMG-935P in mice fed the standard chow diet had no significant effects on liver oxalate, indices of liver damage or lipid metabolism. These extensive, long-term studies not only indicate a causative role of hepatic oxalate overproduction in MASH, but also highlight the therapeutic potential of targeting AGXT, GO and LDHA to lower hepatic oxalate overproduction for the treatment of MASH.

Previous proteomics studies on livers from mice deficient in AGXT revealed alterations in fatty acid metabolic pathways, independent of MASH^60^. Utilizing transcriptomics, we recently reported that the loss of AGXT suppresses FAO pathways and accelerates diet-induced MASH in mice^12^. Considering the emerging reports of suppressed AGXT and impaired glyoxylate/oxalate metabolism in MASLD^12,15–17^, and to address the mechanisms by which oxalate promotes MASH, we studied the effects of oxalate on molecular and metabolic drivers of MASH in hepatocytes. While previous studies in renal cells, monocytes and macrophages used sodium oxalate (NaOX) at doses up to 2 mM (refs. ^22,24,55^), here, we carefully selected the NaOX doses for in vitro studies by treating primary hepatocytes and HepG2 cells with increasing concentrations of NaOX or with PA, which significantly downregulated AGXT and enhanced intracellular oxalate accumulation as found in MASH. Of note, cells treated with NaOX at concentrations lower than 250 µM (primary hepatocytes) or 500 µM (HepG2 cells) did not show intracellular accumulation of oxalate as observed in cells treated with PA or in liver tissues from patients and mice with MASH. At these doses, NaOX significantly enhanced lipid accumulation in both primary hepatocytes and HepG2 cells. In line with our transcriptomics results in livers from *Agxt*^*−*/*−*^ mice^12^, oxalate potently inhibited PPARα-mediated FAO, an established driver of MASH^3,12,61^, with negligible effects on regulators of fatty acid uptake, transport and biosynthesis. Mechanistically, using RNA Pol II inhibition, a bio-orthogonal ‘click’ chemistry approach to measure de novo *PPARA* transcription, PPARα agonism and luciferase assays, we found that oxalate suppresses PPARα transcription and activity, and the expression of PPARα target genes that promote FAO. Notably, restoring PPARα expression or activity abolished oxalate-induced suppression of PPARα target genes and lipid accumulation. Moreover, livers from mice overexpressing AGXT specifically in hepatocytes or treated with MDMG-935P demonstrated lower liver oxalate and steatohepatitis aligned with an upregulation of PPARα target genes. These in vitro and in vivo studies, based on complementary genetic and pharmacological approaches, indicate that suppressed PPARα is a key mediator of oxalate toxicity in the liver.

Previous studies in renal cells, monocytes and macrophages demonstrated that oxalate causes mitochondrial dysfunction leading to enhanced generation of reactive oxygen species and lipid peroxidation^22,24,55^. Consistent with those previous reports, we found that oxalate suppresses mitochondrial respiration in HepG2 cells and enhances mitochondrial superoxide formation. In addition, our studies in primary mouse hepatocytes and human hepatic cell lines uncovered suppressed CPT1α through the inhibition of PPARα activity in response to oxalate. CPT1α critically regulates fatty acid transport into the mitochondria^62^ and FAO is a main pathway for energy production by the mitochondria. Indeed, PPARα overexpression and CPT1α inhibition demonstrated that oxalate impairs mitochondrial respiration mainly through the suppression of FAO. Accordingly, transient overexpression of AGXT not only lowered intracellular oxalate and lipid accumulation, but also significantly upregulated CPT1α and augmented mitochondrial respiration in lipid-loaded HepG2 cells. These findings are supported by our in vivo studies in which *Cpt1a* was significantly upregulated both in livers from mice overexpressing AGXT and in livers from mice treated with MDMG-935P, which not only demonstrated decreased hepatic oxalate, but also reduced steatohepatitis. Thus, while previous reports indicated that oxalate causes mitochondrial dysfunction in various cell types^22,24,55^, our current studies reveal that oxalate impairs mitochondrial respiration in hepatocytes by inhibiting PPARα-regulated FAO leading to intracellular lipid accumulation. These effects are rescued by restoring PPARα expression or activity in vitro and by genetic and pharmacological approaches to lower oxalate overproduction in MASH in vivo.

During the progression of MASH, the accumulation of lipotoxic and reactive oxygen species induce hepatocellular injury, inflammasome activation, release of proinflammatory chemokines with subsequent monocyte infiltration and activation of macrophages and hepatic stellate cells that drive steatohepatitis and hepatic fibrosis^2–4^. While our recent studies in mice deficient in AGXT demonstrated enhanced steatohepatitis and hepatic fibrosis with significant enrichment of proinflammatory and profibrotic pathways^12,24^, the current RNA sequencing findings reveal that proinflammatory (chemokine signalling, cytokine–cytokine receptor interaction, NF-κB and TNF signalling) and profibrotic (focal adhesion signalling, regulation of actin cytoskeleton and ECM–receptor interactions) pathways are significantly suppressed in livers from mice overexpressing AGXT in hepatocytes during MASH. Similar anti-inflammatory and anti-fibrotic effects were found in livers from mice with established MASH that were treated with MDMG-935P. Of note, through histopathological and immunofluorescence analyses of immune cell subsets, we confirmed that lowering oxalate via AGXT overexpression or pharmacological inhibition of LDHA and GO reduces steatohepatitis aligned with the decrease in distinct subsets of recruited monocyte-derived macrophages (CCR2^+^ and TREM2^+^ macrophages) that are known to infiltrate the liver in MASH and form crown-like structures surrounding hepatocytes with large lipid droplets^49^. While these findings can be explained by reduced lipotoxicity secondary to activation of PPARα and improved FAO through lowering of hepatic oxalate, our in vitro studies indicate that upregulated CCL2 due to oxalate overload mediates monocyte chemotaxis. These findings are supported by the downregulation of *Ccl2* and lower monocyte-derived macrophages in livers from mice treated with AAV8-AGXT or MDMG-935P that showed lower liver oxalate, and by previous studies demonstrating that oxalate directly enhances proinflammatory gene expression and stimulates the release of chemoattractant proteins in other cell types^24,63^. Notably, aligned with reduced steatohepatitis, our histopathological and immunofluorescence analyses coupled with biochemical verification revealed that lowering oxalate overproduction in MASH via AGXT overexpression or pharmacological inhibition of LDHA and GO potently reduces hepatic stellate cells and fibrosis, a main determinant of liver-related events and mortality in MASH^50^.

Given that the predominant cause of mortality in patients with MASH is complications due to cardiovascular disease^64^, identifying pathways that can be targeted for simultaneous treatment of MASH and cardiovascular disease is challenging and urgently needed. Emerging studies from our group and others uncovered oxalate metabolism commonly dysregulated in MASLD and cardiovascular disease^12,15,24–26,65^. Notably, increased circulating oxalate was recently identified as a risk factor for cardiovascular events in patients on dialysis^26,65^. Exogenous oxalate not only induces chronic kidney disease, but also causes cardiac fibrosis in C57BL/6 mice^23^, and accelerates atherosclerosis development in apolipoprotein E-deficient (*Apoe*^*−*/*−*^) mice^24^. Similarly, hepatic oxalate overproduction due to the loss of AGXT enhances atherosclerosis, while overexpression of AGXT in hepatocytes lowers oxalate and ameliorates atherosclerosis in *Apoe*^*−*/*−*^ mice^24^. Taken together, the current and previous studies suggest that hepatic oxalate overproduction in the steatotic liver concurrently accelerates the progression of MASH and atherosclerotic cardiovascular disease, highlighting the potential of strategies aimed at lowering oxalate overproduction for dual-targeting of these two prominent diseases. As MDMG-935P potently lowered hepatic oxalate, steatosis, inflammation and fibrosis in mice with established MASH, further evaluation of GO and LDHA inhibition for concurrent treatment of MASH and associated cardiovascular disease is warranted.

Our study has some limitations that may serve as avenues for future research. While we uncovered suppression of AGXT and induction of LDHA as the underlying mechanisms for oxalate overproduction in MASH, the possibility of altered ratios of cofactors for these and other enzymatic reactions (for example, GRHPR) that regulate glyoxylate/oxalate metabolism in MASH warrants further investigation. In addition, we found that plasma oxalate levels were significantly increased in patients with MASH, albeit with high variability. The variability in plasma oxalate measurements may be explained by pre-analytical factors related to sample collection and preparation^66^. While our repository plasma samples were acidified to prevent an excessive formation of oxalate from ascorbate degradation post-collection^67,68^, they were, however, collected in sodium citrate tubes. In contrast, previous studies have reported the use of plasma collected exclusively in sodium heparin tubes^67,68^, which is acceptable for determining oxalate in most clinical laboratories. Given that citrate and heparin inhibit coagulation through distinct mechanisms (citrate binds reversibly to calcium ions, whereas heparin activates anti-thrombin to inhibit coagulation factors), this pre-analytical difference may explain the high variability in plasma oxalate levels observed in the current study.

In summary, combining data from multiple well-defined human cohorts, MASH mouse models and hepatocellular systems, the current study uncovers overproduction of hepatic oxalate due to suppression of AGXT and enhanced LDHA activity that further accelerates MASH through inhibition of PPARα-regulated FAO, enhanced monocyte chemotaxis and ensuing proinflammatory/fibrotic responses. Genetic and pharmacological targeting of hepatic oxalate overproduction lowers hepatic steatosis, inflammation and fibrosis and may have translational potential for the treatment of MASH, currently with limited treatment available. Considering the recent development and approval of therapeutic agents that reduce hepatic oxalate production^52,53^, the safety and efficacy of this strategy as a treatment for MASH warrants further clinical evaluation.

## Methods

### Human studies

The collection of deidentified human plasma and liver specimens was approved by the Institutional Review Board of Ochsner Clinic Foundation (protocols 2010.179, 2016.131.B, and 2020.039). The studies were conducted through the Ochsner Multi-Organ Transplant Institute with specimens collected following informed consent. Liver specimens were obtained during orthotopic liver transplantation due to end-stage liver disease with histologically confirmed stage F4 fibrosis (*n* = 23, 2010.179, 2020.039). Liver specimens were placed into formalin and further processed to formalin-fixed, paraffin-embedded tissue blocks or flash-frozen in liquid nitrogen and stored at −80 °C until analysis. Liver specimens from histologically confirmed (NAS < 1, average steatosis score = 0.3, lobular inflammation score = 0, hepatocellular ballooning score = 0 and fibrosis score = 0) healthy donors (control, *n* = 10) were obtained from BioIVT. Liver specimens from donors with or without MASH were sectioned and stained with H&E. Blinded assessment of NAS was performed by a gastrointestinal pathologist. Steatosis was scored from 0–3 (0, <5% steatosis; 1, 5–33%; 2, 34–66%; and 3, >67%). Hepatocyte ballooning was scored from 0–2 (0, normal hepatocytes; 1, normal-sized with pale cytoplasm; and 2, pale and enlarged hepatocytes, at least twofold). Lobular inflammation was scored from 0–3 based on foci of inflammation counted at 20× (0, none; 1, <2 foci; 2, 2–4 foci; and 3, ≥4 foci). NAS was calculated as the sum of steatosis, hepatocyte ballooning and lobular inflammation scores^69^. Spearman’s correlations between NAS indices and AGXT protein abundance or hepatic oxalate concentrations were evaluated. Peripheral blood specimens from patients with no history of liver disease were obtained before a routine screening colonoscopy or mammogram (2015.101.C). Peripheral blood specimens from patients with MASH, as confirmed by biopsy, magnetic resonance elastography or ultrasonic transient elastography, were obtained during routine hepatology surveillance laboratory visits (2016.131.B). Plasma samples were obtained from peripheral blood specimens in BD Vacutainer CPT mononuclear cell preparation sodium citrate tubes (BD Biosciences, 362761). Plasma was obtained following centrifugation and separation according to the manufacturer’s protocol and stored at −80 °C until analysis. Patient demographics and laboratory values were exported from the electronic medical record and are described in Extended Data Fig. 1.

### Hepatic expression of glyoxylate metabolic genes and MASH

The relationship between the expression of genes regulating glyoxylate metabolism/oxalate formation and hepatic fat content were tested using our previously published microarray data from liver transplantation donors (*n* = 206; GSE26106)^12,28,29^. The tissue dissection, microarray data, and determination of hepatic fat content using hexane/isopropanol extraction were previously described^12,28,29^. Spearman’s correlation was used to determine the significance of the hepatic fat normalized to total protein concentration and transformed to log_10_ scale correlated with the expression of genes regulating glyoxylate metabolism/oxalate formation. The association between the hepatic expression of genes regulating glyoxylate metabolism/oxalate formation and MASH was assessed in patients with or without MASH using two public datasets: (1) liver microarray data (GSE83452) obtained from patients with MASH (*n* = 104) and normal controls (*n* = 44)^30^ and (2) liver microarray data (GSE61260) obtained from patients with MASH (*n* = 24) and healthy obese controls (*n* = 24)^31^. A linear regression model was applied with age, sex and body mass index (BMI) as covariates to identify significant genes regulating glyoxylate metabolism/oxalate formation that are associated with MASH: gene expression = β_MASH × MASH status + β_Age × Age + β_Sex × Sex + β_BMI × BMI + *ε*, where MASH status was coded as 1 for MASH and 0 for healthy controls. A meta-analysis of the two studies with a fixed effect model using the metafor R package was used to increase the statistical power and compare the results. Genes with Benjamini–Hochberg-adjusted *P* < 0.05 and Cochran’s *Q* heterogeneity test *P* > 0.05 were considered as significant.

### Animal studies

All animal procedures were approved by the Institutional Animal Care & Use Committees of Louisiana State University Health Sciences Center-Shreveport (P-21-043, P22-035, and P-24-025) and the University of Michigan (PRO00008239). All studies were performed in accordance with the institutional guidelines. Mice were randomly allocated to treatment groups followed by confirmation of equal body weights before treatment. C57BL/6J (stock 000664) were purchased from The Jackson Laboratories. Eight-week-old C57BL/6J male and female mice were housed under controlled temperature (22 ± 2 °C) and humidity conditions (40–60%) on a 12-h light–dark cycle and fed ad libitum either a standard chow diet (LabDiet, 5053, 13% of calories from fat) or established^12,32–35^ MASH-inducing diets (Research Diets, D17010103, 40% of calories from fat or Envigo, TD.160785, 52.6% of calories from fat) for 12, 16 or 24 weeks before killing and tissue collection. Primary hepatocytes were isolated from 8–10-week-old male C57BL/6J mice fed a standard chow diet as described below. AAV8-AGXT expressing human AGXT and AAV8-GFP control driven by the hepatocyte-specific TBG promoter were administered by intraperitoneal injection into 7-week-old male C57BL/6J mice at 2 × 10^11^ viral genomes and a final volume of 200 µl per mouse, as we previously described^24^. Starting from 8 weeks of age, mice were fed the MASH diet (Research Diets, D17010103) ad libitum for 24 weeks. AAV8-AGXT and AAV8-TBG-GFP were similarly administered to 11-week-old male C57BL/6J mice that were kept on the standard chow (LabDiet, 5053) ad libitum for an additional 12 weeks of age. MDMG-935P is a salicylic acid derivative that we recently developed that potently decreases oxalate production by inhibiting GO and LDHA^54^. The therapeutic potential of MDMG-935P was evaluated in mice with MASH using established protocols^12,32,33,54^. Eight-week-old C57BL/6J male mice were fed the MASH diet (Research Diets, D17010103) ad libitum for 12 weeks. Mice were then randomized to receive MDMG-935P solubilized in 0.5% methylcellulose by oral gavage at a concentration of 0 mg kg^−1^ d^−1^ (vehicle), 5 mg kg^−1^ d^−1^ or 10 mg kg^−1^ d^−1^ for an additional 12 weeks on the MASH diet until killing and tissue collection. Similar studies were performed in mice fed the standard chow diet. Eight-week-old C57BL/6J male mice were fed the standard chow diet (LabDiet, 5053) ad libitum for 12 weeks. Mice were then randomized to receive MDMG-935P solubilized in 0.5% methylcellulose by oral gavage at a concentration of 0 mg kg^−1^ d^−1^ (vehicle) or 10 mg kg^−1^ d^−1^ for an additional 12 weeks on standard chow diet until killing and tissue collection.

### Histology, immunohistochemistry and immunofluorescence

Histological procedures were performed by technicians blinded to experimental groups at the University of Michigan IVAC Histology Laboratory or at Louisiana State University Health Sciences Center-Shreveport as previously described^12,32–34^. In brief, formalin-fixed tissues were sectioned on a M355S rotary microtome (Thermo Fisher Scientific) at 4-μm thickness and mounted on glass slides. Slides were stained for H&E (Thermo Fisher Scientific). For Picrosirius red staining, slides were treated with 0.2 N phosphomolybdic acid for 3 min and transferred to 0.1% Sirius red saturated in picric acid (Rowley Biochemical) for 90 min, then transferred to 0.01 N hydrochloric acid for 3 min. Picrosirius red staining was used to score hepatic fibrosis from 0–4 (0, no fibrosis; 1, perisinusoidal or portal fibrosis; 2, perisinusoidal and portal fibrosis; 3, bridging fibrosis; 4, cirrhosis)^69^. Frozen section processing was used for Oil Red O staining. Formalin-fixed liver samples were cryoprotected in 20% sucrose at 4 °C overnight, blotted, then liquid-nitrogen snap frozen in OCT compound (Tissue-Tek) and stored at −80 °C. Before sectioning, frozen blocks were brought up to about −20 °C, then sectioned at 5 μm on a Cryotome SME (Thermo-Shandon). Before staining, slides were thawed to room temperature for 30 min and then fixed in 10% neutral buffered formalin for 20 min, rinsed in double-distilled water, followed by rinsing in 60% isopropanol before being placed in working ORO-isopropanol stain (Rowley Biochemical, H-503-1B) for 5 min. Slides were then rinsed in 60% isopropanol followed by three changes of double-distilled water and nuclear counterstained with Harris hematoxylin. Immunofluorescence was performed using rat anti-F4/80 (Bio-Rad ABD Serotec, MCA497R, 1:400 dilution), rabbit anti-CCR2 (Abcam, ab273050, 1:250 dilution), rabbit anti-TREM2 (Proteintech, 27599-1-AP, 1:200 dilution), rat anti-Ly6g FITC conjugated (Thermo Scientific, 11-9668-82, 1:150 dilution), mouse anti-smooth muscle actin-Cy3 (1:400 dilution, Sigma, C6198) or rabbit anti-arginase1 (Arg1, Sigma, HPA024006, 1:200 dilution) and nuclei were visualized with 4,6-diamidino-2-phenylindole (DAPI). To visualize F4/80, goat-anti-rat secondary antibody was used (Invitrogen, A-21247, 1:200 dilution). To visualize CCR2 and TREM2, donkey anti-rabbit secondary antibody was used (1:200 dilution, Invitrogen, A-21206). For Nile red in hepatocytes, cells were fixed with 3.7% neutral buffered formalin and stained with Nile red (TCI Chemicals, N0659, 1:2,000 dilution) and DAPI (MP Biometicals, 0215757410, 1:50,000 dilution) for 30 min. For mitochondrial superoxide analysis, cells were treated with MitoSOX superoxide fluorescent dye (Invitrogen, M36008, 4 µM) and Hoechst nuclear stain (Thermo Scientific, 62249) for 30 min. Liver sections and cells were imaged on a Keyence BZ-X810 all-in-one fluorescence microscope. Images were analysed using the Keyence BZ-X800 analyser software.

### RNA isolation and quantitative real-time PCR

For liver tissue samples, RNA was isolated from approximately 50 mg liver tissue. Tissue was lysed using TRIzol (Invitrogen, 15596018) and Precellys soft tissue homogenizing ceramic beads (Cayman Chemical Company, 10011152) in a Precellys Evolution homogenizer (Bertin Technologies). Following isolation of the aqueous layer by chloroform extraction, RNA was isolated using the RNeasy Mini kit (QIAGEN, 74106) as per manufacturer’s instructions. Cells were lysed using the RNeasy Mini kit (QIAGEN, 74106) as per manufacturer’s instructions. Complementary DNA was synthesized using the SuperScript III First-Strand Synthesis System (Invitrogen, 18080-051) as per manufacturer’s instructions. cDNA synthesis was performed in a Mastercycler nexus gradient thermocycler (Eppendorf). Primers were purchased from Integrated DNA Technologies (Supplementary Tables 1 and 2) and qRT–PCR was performed using SSoAdvanced Uniersal SYBR Green Supermix (Bio-Rad, 175271) with a CFX96 Touch Real-Time PCR Detection System (Bio-Rad) according to manufacturer’s instructions. Results were normalized to housekeeping genes (*GAPDH*) and expressed as a fold change from control treatments using the ΔΔCt threshold cycle method of normalization.

### Protein isolation and immunoblotting

For liver tissue samples, protein was isolated from approximately 50 mg liver tissue. Tissue was lysed in RIPA lysis and extraction buffer (G Biosciences, 786-489) supplemented with 1% Halt protease inhibitor cocktail (Thermo Scientific, 78429) and 1% phosphatase inhibitor cocktail A (Alfa Aesar, J65354.LQ) and Precellys soft tissue homogenizing ceramic beads (Cayman Chemical Company, 10011152) in a Precellys Evolution homogenizer (Bertin Technologies). Cells were lysed in either RIPA lysis and extraction buffer (G Biosciences, 786-489) supplemented with 1% Halt protease inhibitor cocktail (Thermo Scientific, 78429) and 1% phosphatase inhibitor cocktail A (Alfa Aesar, J65354.LQ) or 2× Laemmli Sample buffer (Bio-Rad, 1610737). Protein concentrations were adjusted using Quick Start Bradford 1× Dye Reagent (Bio-Rad, 5000205). Membranes were labelled with rabbit anti-AGXT (Sigma, HPA035370, 1:1,000 dilution), mouse anti-AGXT (Santa Cruz Biotechnology, SC-517388, 1:500 dilution), rabbit anti-LDHA (Cell Signalling Technology, 2012, 1:1,000 dilution), rabbit anti-CPT1α (Abcam, ab234111, 1:1,000 dilution), rabbit anti-PPARA (Proteintech, 15540-1-AP, 1:1,000 dilution), rabbit anti-HAO1 (Abcam, 194790, 1:1,000 dilution), mouse anti-β-actin (Cell Signalling Technology, 3700S, 1:1,000 dilution) and mouse anti-GAPDH (Santa Cruz Biotechnology, sc365062, 1:5,000 dilution). Primary antibodies were visualized by fluorescent secondary donkey anti-rabbit antibody (Li-Cor, 926-68073, 1:20,000 dilution) or donkey anti-mouse antibody (Li-Cor, 926-32212, 1:20,000 dilution) on a Li-Cor Odyssey XF Imager. Densitometry was performed using Image Studio Lite v.5.2 software and normalized to GAPDH or β-actin.

### Biochemical analyses of plasma, livers and hepatocytes

Immediately following mouse euthanasia, plasma was separated from whole blood by centrifugation in PST Tubes with lithium heparin (BD Microtainer, 365985). AST and ALT were measured in mouse plasma using the EnzyChrom Aspartate Transaminase or Alanine Transaminase Assay kits (BioAssay Systems, EASTR-100 and EALT-100, respectively) per the manufacturer’s instructions. Human plasma samples were acidified with 10 μl concentrated (12 M) hydrochloric acid per 1.0 ml plasma^67,68^ and assessed for oxalate concentrations using the Oxalate Assay kit (Abcam, ab196990) per the manufacturer’s instructions. For liver oxalate measurements, approximately 50 mg of liver tissue was lysed in ice-cold PBS by sonication. Liver LDH activity was measured using the LDH Assay kit (Abcam, ab102526) according to the manufacturer’s instructions. Primary mouse hepatocytes or HepG2 cells (approximately 5 × 10^5^ cells) were trypsinized, pelleted by centrifugation and lysed in 150 µl ice-cold PBS by sonication. Liver oxalate was measured using the Oxalate Assay kit (Abcam, ab196990) and normalized to tissue weight. For triglyceride analysis, approximately 50 mg frozen liver samples were homogenized in PBS and the soluble fraction was removed by centrifugation. Lipids were extracted as described previously^12,32^. In brief, lipids were extracted using 3:2 hexane:isopropanol. Aqueous components were separated out with the addition of PBS, and the remaining hexane fraction was permitted to evaporate for 48 h. Triglycerides were determined using the LabAssay Triglyceride measurement kit (Fuji Film, 632-50991) as per the manufacturer’s instructions. Liver hydroxyproline was measured using a hydroxyproline assay kit (Abcam, ab222941) as per the manufacturer’s instructions. MDA was measured using the TBARS assay kit (Cayman Chemical Company, 10009055) according to the manufacturer’s instructions.

### Oxalate analysis using IC–MS

Approximately 20–30 mg liver tissue was snap frozen in liquid nitrogen, then homogenized with Precellys Tissue Homogenizer. Oxalate was extracted using ice-cold 80:20 (*v*/*v*) methanol:water with 0.1% ammonium hydroxide. Extracts were vortexed vigorously for 2 min, centrifuged at 17,000*g* for 10 min at 4 °C and supernatants were transferred to clean tubes, followed by evaporation to dryness under nitrogen. Dried extracts were reconstituted in 100 μl 1 mM potassium hydroxide (KOH) in deionized water and 10 μl were injected for analysis by IC–MS. The IC mobile phase A (MPA; weak) was water and the mobile phase B (MPB; strong) was water containing 100 mM KOH. A Thermo Scientific Dionex ICS-6000+ system, including a Thermo IonPac AS11 column (4-µm particle size, 250 × 2 mm), with the column compartment kept at 35 °C, was used for separating metabolites. The autosampler tray was chilled to 4 °C. The mobile phase flow rate was 360 µl min^−1^ and the gradient elution programme was 0–5 min, 1% MPB; 5–25 min, 1–35% MPB; 25–39 min, 35–99% MPB; 39–49 min, 99% MPB; 49–50 min and 99–1% MPB. The total run time was 55 min. To enhance sensitivity, methanol was delivered by an external pump and combined with the eluent via a low dead volume mixing tee. Data were acquired using a Thermo Orbitrap IQ-X Tribrid Mass Spectrometer under negative electrospray ionization.

### Reduced representation bisulfite sequencing

Unbiased genome-wide DNA methylation analysis was carried out by Active Motif. Genomic DNA was extracted from livers of mice fed the MASH-inducing diet (Research Diets, D17010103) or standard chow diet (LabDiet, 5053) for 24 weeks using the Quick-gDNA MiniPrep kit (Zymo Research, D3024) following the manufacturer’s instructions. Samples were first Proteinase K digested (0.5% SDS, 0.5 mg ml^−1^ PK, 100 mM EDTA, in TE pH 8) rotating at 55 °C overnight. For library preparation and sequencing, 100 ng of genomic DNA was digested with TaqaI (New England Biolabs, R0149) at 65 °C for 2 h followed by MspI (New England Biolabs, R0106) at 37 °C overnight. Following enzymatic digestion, samples were used for library generation using the Ovation RRBS Methyl-Seq System (Tecan, 0353-32) following the manufacturer’s instructions. In brief, digested DNA was randomly ligated and, following fragment end repair, bisulfite was converted using the EpiTect Fast DNA Bisulfite kit (QIAGEN, 59824) following the manufacturer’s protocol. After conversion and cleanup, samples were amplified resuming the Ovation RRBS Methyl-Seq System protocol for library amplification and purification. Libraries were measured using Agilent 2200 TapeStation System and quantified using the KAPA Library Quant kit ABI Prism qPCR Mix (Roche, KK4835). Libraries were sequenced on a NovaSeq 6000 at SE75. Reads were mapped to the genome using Bismark (v.0.23.0) and Bowtie2 (v.2.4.2) allowing for no mismatches (-N 0) and a seed substring length of 20 (-L 20). Following alignment, PCR duplicates were removed using a custom analysis script. Each read had a randomized 6-mer barcode and if more than one read had the same start and end coordinates and the same barcode, all but one of the reads were discarded. CpG reports from the Bismark alignment were processed with the methylKit R package (v.1.28.0) and only CpG sites covered with at least ten reads were retained for the downstream analyses. MethylKit was used to perform Fisher’s exact test pairwise comparisons between the samples from mice with or without MASH. The raw RRBS data have been deposited in NCBI’s Gene Expression Omnibus (GEO) database under accession no. GSE265985.

### Cloning and production of AAV8-GFP and AAV8-AGXT

AAV8-AGXT expressing the human AGXT and AAV8-GFP control were prepared as we previously described^24^. In brief, plasmids for AAV8 package (pAdDeltaF6, pAAV2/8, pAAV-TBG-GFP, pAAV-TBG-MCS) were kindly provided by J. Lin (University of Michigan). The human *AGXT* was cloned from plasmid #RG212899 (Origene) into the backbone plasmid pAAV-TBG-MCS using the Gibson assembly kit (New England Biolabs). The *AGXT* sequence and proper insertion were confirmed by Sanger sequencing. pAAV-TBG-GFP, with the same backbone but expressing GFP, was used as a control. Seventy micrograms of AAV shuttle vector, 200 µg Delta F6 helper plasmid and 70 µg AAV2/8 Rep/Cap plasmid were prepared with EndoFree Plasmid Maxi kit (QIAGEN) and transfected into 15-cm plates of HEK293T cells using PEI transfection reagent (Sigma-Aldrich). After 96 h, the cells were lysed (20 mM Tris, pH 8.0, 150 mM NaCl) and 1 M MgCl_2_ and 25 kU ml^−1^ Benzonase were added after three freeze–thaw cycles between liquid nitrogen and 37 °C. Cell lysates were incubated at 37 °C for 15 min and then centrifuged at 1,500*g* and 4 °C for 30 min. AAVs in the supernatant were purified by ultracentrifugation in a density gradient iodixanol solution with a T865 rotor for 160 min at 367,000*g* and 14 °C. AAVs were concentrated in PBS with 0.01% Poloxamer 188 (Sigma-Aldrich) using a 100-kDa filter tube (Millipore, 910096) and the titre was quantified by qPCR.

### RNA sequencing and data analysis

As described above, RNA was isolated from liver samples of mice treated with AAV8-GFP or AAV8-AGXT (*n* = 4 per group, randomly selected). Samples were quantitated with a Qubit RNA assay (Thermo Fisher Scientific) and RNA quality was determined with the Agilent TapeStation RNA assay (Agilent Technologies). All samples had RNA integrity numbers of at least 8.3. Libraries were prepared with the Stranded mRNA Prep, Ligation kit (Illumina). One µg of RNA was processed for each sample and mRNA was purified and fragmented. cDNA was synthesized, and 3′ ends were adenylated. Anchor sequences were ligated to each sample and a limited-cycle PCR was performed to amplify and index the libraries. The average library size was determined using an Agilent TapeStation D1000 assay (Agilent Technologies) and libraries were quantitated with qPCR (Bio-Rad CFX96 Touch Real-Time PCR, NEB Library Quant kit for Illumina). Libraries were normalized to 0.5 nM and pooled. The library pool was denatured and diluted to approximately 100 pM. A 1% library of 2.5 pM PhiX was spiked in as an internal control. Paired end 76 × 76-bp sequencing was performed on an Illumina NovaSeq 6000. Primary analysis, including base calling and quality scoring, was performed onboard the Illumina NovaSeq 6000 (NovaSeq Control Software v.1.8.0; RTA v.3). Samples were de-multiplexed, the adaptor sequences were removed (the first nine cycles of sequencing were trimmed) and FASTQ files were generated. Data analysis was performed as we previously described^12,24,32,34^. The quality of the raw FASTQ files was checked through FastQC v.0.11.8 (https://www.bioinformatics.babraham.ac.uk/projects/fastqc/). Trimmomatic v.0.35 was used to trim the low-quality reads with the parameters: SLIDINGWINDOW:4:20 MINLEN:25. The resulted high-quality reads were then mapped to the mouse reference genome (GRCm38.90) using HISAT2 v.2.1.0.13. Gene level quantification was performed using HTSeq-counts v.0.6.0 based on the GRCm38.90 genome annotations. The R package DESeq2 v.1.42.1 was then used to identify significant DEGs. Genes with an adjusted *P* value < 0.05 were considered significant. The upregulated and downregulated DEGs were analysed for significantly enriched KEGG pathways using the clusterProfiler v.4.10.1 package. The significance of the enrichment was determined by a right-tailed Fisher’s exact test followed by Benjamini–Hochberg multiple testing adjustment. The raw RNA sequencing data have been deposited in NCBI’s GEO database under accession no. GSE224097.

### Untargeted lipidomics and data analysis

Approximately 30 mg liver tissue from mice treated with AAV8-AGXT or AAV8-GFP was used for untargeted lipidomics. Hepatic lipids were extracted by adding 150 µl ice-cold ethanol containing 1% 10 mM butylated hydroxytoluene and 2% Avanti SPLASH LIPIDOMIX MS Standards to each liver sample. The liver samples were then homogenized with Precellys Tissue Homogenizer. Next, the samples were vortexed for 5 min, placed on ice for 10 min and centrifuged at 17,000*g* for 10 min at 4 °C. The supernatants were then collected for LC–MS analysis. Mobile phase A (MPA) was 40:60 acetonitrile: water with 0.1% formic acid and 10 mM ammonium formate. MPB was 90:9:1 isopropanol:acetonitrile: water with 0.1% formic acid and 10 mM ammonium formate. The chromatographic method included a Thermo Fisher Scientific Accucore C30 column (2.6 µm, 150 × 2.1 mm) maintained at 40 °C, a mobile phase flow rate of 0.200 ml min^−1^, an autosampler tray chilling at 8 °C and a gradient elution programme as follows: 0–3 min, 30% MPB; 3–13 min, 30–43% MPB; 13.1–33 min, 50–70% MPB; 33–48 min, 70–99% MPB; 48–55 min, 99% MPB; and 55.1–60 min, 30% MPB. The injection volume was 10 µl. A Thermo Fisher Scientific Orbitrap Fusion Lumos Tribrid mass spectrometer with heated electrospray ionization source was operated in data dependent acquisition mode, in both positive and negative ionization modes, with scan ranges of 150–1,500 *m/z*. An Orbitrap resolution of 240,000 (full width at half maximum) was used for MS^1^ acquisition and spray voltages of 3,600 and −2,900 V were used for positive and negative ionization modes, respectively. Vaporizer and ion transfer tube temperatures were set at 275 °C and 300 °C, respectively. The sheath, auxiliary and sweep gas pressures were 35, 10 and 0 (arbitrary units), respectively. For MS^2^ and MS^3^ fragmentation a hybridized HCD/CID approach was used. Data were analysed using Thermo Scientific LipidSearch software (v.5.1) and R scripts written in house. The raw untargeted lipidomics data have been deposited in MassIVE (accession no. MSV000094587).

### Primary hepatocyte isolation

Primary hepatocytes were isolated from 8–10-week-old male C57BL/6J mice fed a standard diet. Following euthanasia by isoflurane, the portal vein was cannulated using a 24G IV catheter (Terumo, SR-OX2419CA). The catheter was held within the portal vein by applying a surgical knot. The inferior vena cava was cut and 50 mL of warm liver perfusion medium (Gibco, 17701038 supplemented with 1% penicillin/streptomycin (GenClone, 25-512)) was perfused through the liver at 5 ml min^−1^. Pressure was applied to the inferior vena cava using a sterile cotton swab every 10 ml for approximately 15 s to allow backflow and thorough perfusion. Liver digest medium (Gibco, 17703034; warmed to 40 °C) was perfused through the mouse at 5 ml min^−1^. Pressure was applied to the inferior vena cava using a sterile cotton swab every 10 ml for approximately 15 s to allow backflow and thorough digestion. The liver was removed from the body and the gallbladder was removed. Cells were dissociated from the liver by gentle mechanical separation using forceps in ice-cold plating and thawing medium (William’s E Medium (Gibco, A1217601) supplemented with thawing and plating supplements (Gibco, CM3000)) and passed through a 100-µm filter. Cells were rinsed twice by centrifugation at 50*g* for 3 min and purified by Percoll gradient (20% Percoll (Cytiva, 45-001-748), 80% thawing and plating medium). Percoll was removed by centrifugation at 150*g* for 3 min, and cells were rinsed twice by centrifugation at 50 g for 3 min. Cells were resuspended in warm thawing and plating medium and plated at approximately 80% confluence into collagen-coated plates (0.01% *w*/*v*, Advanced Biomatrix NC0476635). Cells were allowed to adhere for approximately 3–4 h and non-adhered cells and debris were rinsed with warm PBS. Hepatocyte maintenance medium (William’s E Medium (Gibco, A1217601) with maintenance supplements (Gibco, CM4000)) was added for experiments.

### Cell culture, treatments and polarized microscopy

Mouse primary hepatocytes were maintained in maintenance medium and utilized for experiments no longer than 24 h post-isolation. Hepatocytes were plated at approximately 80% confluence and allowed to adhere for approximately 6 h before treatment. HepG2 cells were maintained in DMEM (Gibco, 1059-010) supplemented with 10% FBS (Gibco, 10438-026) and 1% penicillin/streptomycin (GenClone, 25-512) and plated at approximately 5 × 10^4^ cells per cm^2^. Cells were treated with sodium oxalate (NaOX, 0–500 µM, Sigma, 223433), BSA-conjugated PA (200 µM, Cayman Chemical Company, 29558), BSA control (200 µM, Cayman Chemical Company, 29556) or Wy 14,643 (10 µM, Cayman Chemical Company, 70730). For inhibition of transcription, HepG2 cells were treated with actinomycin D (5 μg ml^−1^, Sigma-Aldrich, A1410) for 24 h and lysed for qRT–PCR analysis as described above. Assessment of calcium-oxalate deposition as birefringent crystals in HepG2 cells and primary hepatocytes treated with increasing concentrations of NaOX (0–10 mM) was performed using a Nikon Eclipse TS2R-FL microscope equipped with a polarized light filter.

### Click-iT nascent RNA capture assay

The assessment of *PPARA* mRNA turnover utilized the Click-iT Nascent RNA Capture kit (Life Technologies, C10365) following the manufacturer’s instructions. HepG2 cells were cultured in the presence or absence of NaOX (500 µM) with or without the alkyne-modified nucleoside EU at a concentration of 0.25 mM. Next, the cell culture medium was aspirated and cells were rinsed to eliminate the uridine analogue. Cell lysis ensued, and mRNA was extracted. Click-iT reactions were executed to conjugate biotinylated azide to EU-labelled mRNA, utilizing 0.5 mM of biotinylated azide per 1 mg of mRNA. EU-labelled mRNAs were then captured and precipitated via streptavidin T1 magnetic beads, which underwent meticulous washing before proceeding to cDNA synthesis for subsequent quantification of *PPARA* gene expression via qRT–PCR.

### Transfections and luciferase assays

For in vitro PPARα overexpression, HepG2 cells were seeded at a density of 2.5 × 10^5^ cells per well in a 12-well plate and treated with Lipofectamine 3000 following the manufacturer’s protocol. Cells were transfected with either pcDNA3.1-GFP control (1 µg DNA per 1.5 × 10^5^ cells) or pcDNA3.1/hPPARα (1 µg DNA per 1.5 × 105 cells, OriGene, NM_000030). After 24 h of transfection period, cells were subjected to treatment with or without NaOX (500 µM) overnight. Subsequently, cells were lysed to extract RNA and protein for the assessment of gene and protein expression levels or Seahorse analyses. For in vitro AGXT overexpression, HepG2 cells were plated at 2.5 × 10^5^ cells per well in a 12-well plate with Lipofectamine 3000 according to manufacturer’s instructions with pcDNA3.1-GFP control (1 µg DNA per 2 × 10^5^ cells) or pcDNA3.1-AGXT-GFP (1 µg DNA per 2 × 10^5^ cells, OriGene, NM_000030). For luciferase reporter assays, 1 × 10^4^ HepG2 cells per well were plated in a 96-well plate and transfected with 80 ng per well of PPREx3-TK-luciferase (pGL3/PPREx3, Addgene), 10 ng per well recombinant human PPARα (pcDNA3.1/hPPARα, NM_001001930) and 10 ng per well *Renilla* (pRL-TK, Promega) constructs using Lipofectamine 3000 (Invitrogen, L3000-015). Approximately 18 h following transfection, cells were treated with or without NaOX (500 µM) or Wy 14,643 (10 µM) for 24 h. Cells were lysed and luminescence was measured (Promega, E1980) as per manufacturer’s instructions on a CLARIOstar Plus High-Performance Multimode Microplate Reader.

### Seahorse analysis

Oxygen consumption rates (OCRs) and dependency on FAO were assessed using an Agilent Seahorse XFe24 Analyser at the Cellular Metabolism Core, Louisiana State University Health Sciences Center-Shreveport. As we previously described^12^, HepG2 cells were seeded at 2.5 × 10^4^ per well in XF24 cell culture microplates (Agilent, 103015-100). The next day, cells were treated with or without 500 µM NaOX for approximately 18 h. For the overexpression of AGXT or PPARα, HepG2 cells were seeded at a density of 1 × 10^4^ cells per well in a XF24 cell culture microplate. Using Lipofectamine 3000, cells were transfected with either pcDNA3.1-GFP control, pcDNA3.1-AGXT-GFP or pcDNA3.1/hPPARα. After 24 h, cells were subjected to treatment with PA (200 µM) or NaOX (500 µM) overnight. XFe24 sensor cartridges were hydrated in accordance with the manufacturer’s protocol. Oligomycin, FCCP, rotenone + antimycin A (R/A) (Agilent, 103015-100) and etomoxir (Cayman Chemical Company, 11969) were used at final concentrations of 2.5 µM, 1 µM, 0.5 µM, and 20 µM, respectively.

### Human peripheral blood monocyte isolation

hPBMs were isolated according to the Institutional Review Board and Health Insurance Portability and Accountability Act guidelines (approval no. H99-064) as described previously^70^. In brief, blood was drawn by median cubital vein venipuncture from healthy volunteers and centrifuged through a Ficoll Histopaque 1077 gradient (Sigma) to isolate mononuclear cells. Cells were then washed with saline and monocytes were isolated by centrifugation through a Percoll (Pharmacia) gradient. Cells were washed once in serum-free RPMI medium and resuspended in serum-free RPMI medium. hPBMs were used within 24 h. To label monocytes with green fluorescence, hPBMs were suspended in warm HBSS (Gibco, 14025-076) at a concentration of approximately 1 × 10^6^ cells per ml and 5 µl ml^−1^ of Vybrant DiO cell-labelling solution (Invitrogen, V22886) was added as per manufacturer’s instructions. Cells were incubated at 37 °C for 20 min, then rinsed twice with fresh HBSS before a final resuspension of 5 × 10^6^ cells per ml before Transwell experiments. HepG2 cells were plated at 1 × 10^5^ cells per well of a 24-well plate and allowed to adhere overnight in 500 µl DMEM supplemented with 10% FBS and 1% penicillin/streptomycin.

### Transwell chemotaxis assay

HepG2 cells were then treated with or without 500 µM NaOX for approximately 18 h. For *CCL2* knockdown experiments, HepG2 cells were plated at 1 × 10^5^ cells per well of a 24-well plate and transfected with 20 nM of siRNA against *CCL2* (siCCL2, CCL2 Human ON-TARGETplus siRNA, Dharmacon, L-007831-00-000) using Lipofectamine RNAiMAX (Invitrogen, 13778150) in Opti-MEM reduced-serum medium (Gibco) in accordance with the manufacturer’s protocol. Scrambled siRNA was used as a negative control (ON-TARGETplus non-targeting pool, Dharmacon, CO, D-001810-10-05). After 24 h, cells were treated with or without 500 µM NaOX for 18 h. hPBMs were labelled with Vybrant DiO cell-labelling solution and suspended at approximately 5 × 10^6^ cells per ml of warmed HBSS. Millicell cell culture hanging inserts with 8-µm pores (Millipore, PTEP24H48) were inserted into each well and 100 µl hPBMs were added to the top well of each insert. hPBMs were permitted to incubate for approximately 18 h in the insert and pass through the pores into the bottom well. Following hPBM transmigration, inserts were carefully removed and the bottom well containing both HepG2 cells and hPBMs were fixed in 3.7% neutral buffered formalin for at least 20 min. Cells were visualized on a Keyence BZ-X810 all-in-one fluorescence microscope and the total number of hPBMs that passed into the bottom well were quantified using Keyence BZ-X800 analyser software. Representative images of the fluorescent hPBMs (shown in green) were taken with a brightfield overlay to visualize equal numbers of HepG2 cells for each treatment.

### Statistical analyses

All statistical analyses were performed using GraphPad Prism v.10 software. No statistical methods were used to pre-determine sample sizes, but our sample sizes are similar to those reported in previous publications^11,12,15,24,34^. The following analyses were conducted by technicians or investigators blinded to the experimental groups: histopathology, RNA sequencing, lipidomics, IC–MS and RRBS. All other data collection and analyses were not performed blind to the conditions of the experiment. All data were expressed as mean ± s.e.m. and repeated with at least three independent experiments. Biological replications were performed as indicated and averaged for each individual experiment. Each data point presented represents an independent experiment or an individual subject. Due to insufficient human liver tissue, one MASH sample was excluded from the histopathological analysis and three MASH samples were excluded from the LDH activity assay. Before statistical comparisons, data were tested for equal variance and normality using Shapiro–Wilk and Kolmogorov–Smirnov tests. If data passed, an unpaired *t*-test was used to compare two groups and a one-way ANOVA followed by Tukey’s post hoc test for comparisons among more than two groups. Otherwise, nonparametric tests (Mann–Whitney *U*-test or Kruskal–Wallis test followed by Dunn’s post hoc test) were used. Data comparing multiple groups utilized two-way analysis of variance (ANOVA) with Bonferroni multiple comparisons test. Differences between categorical variables (sex and race) were tested using chi-squared analysis. *P* < 0.05 was considered statistically significant.

### Reporting summary

Further information on research design is available in the Nature Portfolio Reporting Summary linked to this article.

## Supplementary information

Supplementary Information Supplementary Fig. 1 and Tables 1 and 2.

Reporting Summary

## Source data

Source Data Fig. 1 Statistical source data.

Source Data Fig. 1 Unprocessed western blots/gels.

Source Data Fig. 2 Statistical source data.

Source Data Fig. 2 Unprocessed western blots/gels.

Source Data Fig. 3 Statistical source data.

Source Data Fig. 4 Statistical source data.

Source Data Fig. 4 Unprocessed western blots/gels.

Source Data Fig. 5 Statistical source data.

Source Data Fig. 6 Statistical source data.

Source Data Fig. 7 Statistical source data.

Source Data Fig. 8 Statistical source data.

Source Data Extended Data Fig. 1 Statistical source data.

Source Data Extended Data Fig. 1 Unprocessed western blots/gels.

Source Data Extended Data Fig. 2 Statistical source data.

Source Data Extended Data Fig. 2 Unprocessed western blots/gels.

Source Data Extended Data Fig. 3 Statistical source data.

Source Data Extended Data Fig. 3 Unprocessed western blots/gels.

Source Data Extended Data Fig. 4 Statistical source data.

Source Data Extended Data Fig. 4 Unprocessed western blots/gels.

Source Data Extended Data Fig. 5 Statistical source data.

Source Data Extended Data Fig. 6 Statistical source data.

Source Data Extended Data Fig. 7 Statistical source data.

Source Data Extended Data Fig. 7 Unprocessed western blots/gels.

Source Data Extended Data Fig. 8 Statistical source data.

Source Data Extended Data Fig. 9 Statistical source data.

Source Data Extended Data Fig. 10 Statistical source data.

Source Data Extended Data Fig. 10 Unprocessed western blots/gels.

## Data Availability

All data are available within the paper, extended data, source data files and supplementary files. Raw RNA sequencing and RRBS data have been deposited in NCBI’s GEO database under accession nos. GSE224097 and GSE265985, respectively. Raw lipidomics data have been deposited in MassIVE under accession no. MSV000094587. Source data are provided with this paper.
