# Supplementary material for: Inhibition of hepatic oxalate overproduction ameliorates metabolic dysfunction-associated steatohepatitis
Source: Nat Metab. 2024 Sep 27;6(10):1939–62. doi: 10.1038/s42255-024-01134-4 (PMC11495999; doi:10.1038/s42255-024-01134-4)
Supplement: Supplementary file 1 — Supplementary Fig. 1 and Tables 1 and 2. [file 42255_2024_1134_MOESM1_ESM.pdf]

nature metabolism

Supplementary information https://doi.org/10.1038/s42255-024-01134-4

Inhibition of hepatic oxalate overproduction ameliorates metabolic dysfunction-associated steatohepatitis

In the format provided by the authors and unedited

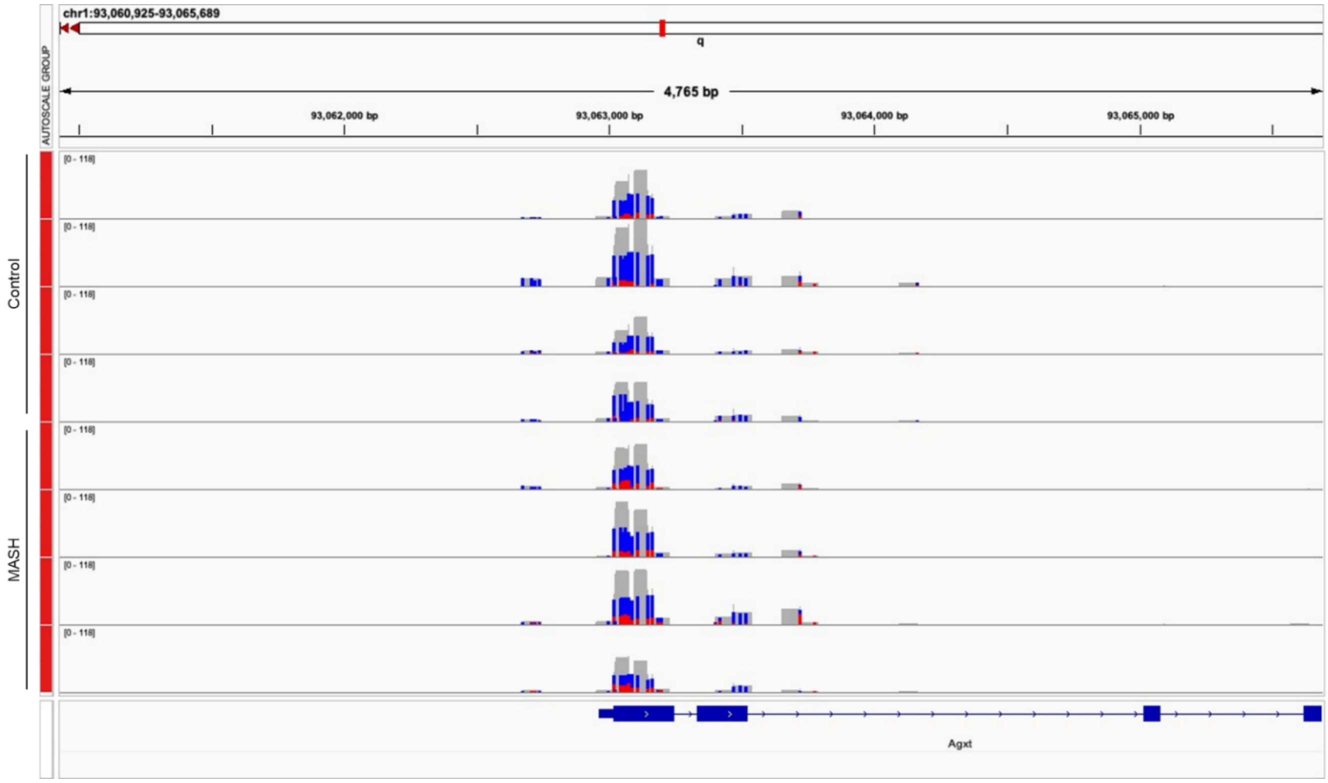

Supplementary Fig. 1: Hypermethylation of AGXT in MASH: Integrative Genomics Viewer (IGV) screenshot showing methylation rates (red) at the promoter and first exon region of Agxt in livers from mice with advanced MASH (24 weeks on the MASH diet) or controls (n=4).

Supplementary Table 1: Human primers used for qRT-PCR analyses

Gene name (Human)	Forward primer	Reverse primer
Alanine-glyoxylate aminotransferase (*AGXT)*	AGAGACATCGTCAGCTACGTCA	CAGGTCACAGCTTCTTCTTGG
Glycolate reductase/hydroxypyruvate reductase (*GRHPR*)	GATGTCCTGACAGATACCACCG	TCTGCGTGAGTCCATAGCCACA
Hydroxyproline dehydrogenase (*PRODH2*)	GGACCTGTTACGTGCTCTGTT	AGACGGATGCTCGGAGAAATG
4-Hydroxy-2-oxoglutarate aldolase 1 (*HOGA1*)	GGTCCCCAAGTCTGGTCTTCT	GATACCCGCAATGTCCACCTT
Hydroxyacid oxidase 1 *(HAO1)*	GTCCTGAGGCACTTCGTTGG	CCCAGGTAAGGTGTGTCCA
Lactate dehydrogenase (*LDHA*)	TATGGAGTGGAATGAATGTTGC	CCCTTAATCATGGTGGAAACTC
Glyceraldehyde-3-phosphate dehydrogenase (*GAPDH*)	ACAACTTTGGTATCGTGGAAGG	GCCATCACGCCACAGTTTC
Peroxisome proliferator activated receptor alpha (*PPARA*)	CGGTGACTTATCCTGTGGTCC	CCGCAGATTCTACATTCGATGTT
PPARG coactivator 1 alpha (*PPARGC1A*)	GCTTTCTGGGTGGACTCAAGT	GAGGGCAATCCGTCTTCATCC
Carnitine palmitoyltransferase 1A (*CPT1A*)	TCCAGTTGGCTTATCGTGGTG	TCCAGAGTCCGATTGATTTTTGC
Acyl-CoA Dehydrogenase Medium Chain (*ACADM*)	ACAGGGGTTCAGACTGCTATT	TCCTCCGTTGGTTATCCACAT
Sterol regulatory element binding transcription factor (*SREBF1*)	GGCTCCTGCCTACAGCTTCT	CAGCCAGTGGATCACCACA
Fatty acid synthase (*FASN*)	TATGCTTCTTCGTGCAGCAGTT	GCTGCCACACGCTCCTCTAG
Acetyl-CoA carboxylase alpha (*ACACA*)	CAGAAGTGACAGACTACAGG	ATCCATGGCTTCCAGGAGTA
Steroyl-Coenzyme A desaturase (*SCD1*)	CACCACATTCTTCATTGATTGCA	ATGGCGGCCTTGGAGACT
Cluster of differentiation 36, fatty acid translocase (*CD36)*	AAGCCAGGTATTGCAGTTCTTT	GCATTTGCTGATGTCTAGCACA

Solute carrier family 27A1-2, fatty acid transporter protein 2 (*SLC27A2)*	GGAGATACATTCCGGTGGAA	TGATCTCAATGGTGTCCTGT
Solute carrier family 27A1-2, fatty acid transporter protein 4 (*SLC27A4)*	GTGAAGGCAAAGGTGCGAC	CGGAAGGTCCAGTGGGTATC
Solute carrier family 27A1-2, fatty acid transporter protein 5 (*SLC27A5)*	TGGAGGAGATCCTTCCCAAGC	TGGTCCCCGAGGTATAGATGAA
C-C motif chemokine ligand 2 (*CCL2*)	GCTCAGCCAGATGCAATCA	AGATCTCCTTGGCCACAATG

Supplementary Table 2: Mouse primers used for qRT-PCR analyses

Gene Name (Mouse)	Forward Primer	Reverse Primer
Alanine-glyoxylate aminotransferase (*Agxt)*	AAGGCATCCAGTATGTGTTCCA	TTCCGGTTAGAAAGGAGTCCC
Glycolate reductase/ hydroxypyruvate reductase (*Grhpr*)	GTGTCCTGACAGATGCCACTG	CACATCCATAATGGGCTCCAG
Hydroxyproline dehydrogenase (*Prodh2*)	GCCTTCCATGTCAAGGGAACC	CCTGAAACGCTAGTCCATGAGT
4-Hydroxy-2-oxoglutarate aldolase 1 (*Hoga1*)	GCAGGGGCTTGTCTAGGAATG	TCAGGAACGGAAACTCTCCAG
Hydroxyacid oxidase 1 *(Hao1)*	TTGGGCTACCTCCTCAATAGAA	TCTGTCTGCTGATCTCACGGT
Lactate dehydrogenase (*Ldha*)	CAAAGACTACTGTGTAACTGCGA	TGGACTGTACTTGACAATGTTGG
Glyceraldehyde-3-phosphate dehydrogenase (*Gapdh*)	CTGCGACTTCAACAGCAACT	GAGTTGGGATAGGGCCTCTC
Peroxisome proliferator activated receptor alpha (*Ppara*)	AACATCGAGTGTCGAATATGTGG	CCGAATAGTTCGCCGAAAGAA
Peroxisome proliferator activated receptor alpha (*Ppargc1a*)	ATCACGTTCAAGGTCACCCTAC	TTCTGCTTCTGCCTCTCTCTCT
Carnitine palmitoyltransferase 1A (*Cpt1a*)	AGATCAATCGGACCCTAGACAC	CAGCGAGTAGCGCATAGTCA
Acyl-CoA Dehydrogenase, Medium Chain (*Acadm*)	CTGTGATTCTTGCTGGAAATGA	GCCGTTGATAACATACTCGTCA
Acyl-CoA Dehydrogenase, long Chain (*Acadl*)	CTATATTGCGAATTACGGCACA	ACACCTTGCTTCCATTGAGAAT
Acyl-CoA Dehydrogenase very long Chain (*Acadvl*)	GCAGATGAGTGCATCCAAATAA	TGAGTTCCTTTCCTTTGTCCAT

Acyl-Coenzyme A oxidase 1 (Acox1*)*	CCGCCACCTTCAATCCAGAG	CAAGTTCTCGATTTCTCGACGG
Hydroxyacyl-Coenzyme A dehydrogenase α (*Hadha*)	ACCTCGGTGTAAAGCACAAAGT	GAGGTTTTGTCAGTGGTGATGA
Hydroxyacyl-Coenzyme A dehydrogenase β (*Hadhb*)	AAACAAGCAATGTGGCTAGAGAG	CAGACATTAACTCAACACCACCA
Acetyl-Coenzyme A acyltransferase 2 (*Acaa2*)	AGTCCCCCTACTGTGTCAGAAA	CCATCTCCTCATTGAAGTAGCC
Enoyl-Coenzyme A Δ isomerase 1 (*Eci1*)	TGCTGTGACTACAGGGTTATGG	GATCCTCAGGTACCACCTCATC
Enoyl-Coenzyme A Δ isomerase 2 (*Eci2*)	ACTACTGCAGTGGGAATGACCT	ATAGTCCCAGAAGGGTGACAGA
Acyl-CoA Synthetase Long Chain 1 (*Acsl1*)	GAGCAATGATCACTCACCAAAA	TCTTAGCTCCATGACACAGCAT
Fatty acid translocase or Cluster of differentiation 36 (*Cd36*)	GAGCCATCTTTGAGCCTTCA	TCAGATCCGAACACAGCGTA
Solute carrier family 27A1-2, fatty acid transporter protein 5 (*Slc27a5*)	CACCCCCAGGGCTACGCT	CAGTGCTTGCCGCTCTAAA
Solute carrier family 27A1-2, fatty acid transporter protein 2 (*Slc27a2*)	CTGTTCCGAGACGAGACGC	TGGCACGAATGTTGTAGTTGAG
Solute carrier family 27A1-2, fatty acid transporter protein 4 (*Slc27a4*)	TGAAATCACCGCAGACGACAGG	GCTTGTCACCATCTCGTTTTCTC
Sterol regulatory element binding transcription factor (*Srebpf1*)	TAGAGCATATCCCCCAGGTG	GGTACGGGCCACAAGAAGTA
Fatty acid synthase (*Fasn*)	CTCATCCACTCAGGTTCAG	AGGTATGCTCGCTTCTCT

Acetyl-CoA carboxylase alpha (*Acaca*)	AATGAACGTGCAATCCGATTTG	ACTCCACATTTGCGTAATTGTTG
Steroyl-Coenzyme A desaturase (*Scd1*)	CGAGGGTTGGTTGTTGATCT	GCCCATGTCTCTGGTGTTTT
C-C motif chemokine ligand 2 (*Ccl2*)	TTAAAAACCTGGATCGGAACCAA	GCATTAGCTTCAGATTTACGGGT
C-C motif chemokine ligand 5 (*Ccl5*)	ATATGGCTCGGACACCACTC	CCACTTCTTCTCTGGGTTGG
C-C motif chemokine receptor 2 (*Ccr2*)	GATGATGGTGAGCCTTGTCATA	AGTGAGCCCAGAATGGTAATGT
Nuclear factor of kappa light polypeptide gene enhancer in B cells 2, p49/p100 (*Nfkb2*)	GAGGTTCGGTTCTATGAGGATG	CTCTGCACTTCCTCCTTGTCTT
Avian reticuloendotheliosis viral (v-rel) oncogene related B (*Relb*)	ATCCTCTCTGAGCCTGTCTACG	CACATCAGCTTGAGAGAAGTCG
Tumor necrosis factor (*Tnf*)	CTGTGAAGGGAATGGGTGTT	GGTCACTGTCCCAGCATCTT
Transforming growth factor β-1 (*Tgfb1*)	TGCGCTTGCAGAGATTAAAA	CTGCCGTACAACTCCAGTGA
Transforming growth factor β-2 (*Tgfb2*)	CTAATGTTGTTGCCCTCCTACAG	GCACAGAAGTTAGCATTGTACCC
Transforming growth factor β receptor type 1 (*Tgfbr1*)	GGACCATTGTGTTACAAGAAAGC	CATGGCGTAACATTACAGTCTGA
Transforming growth factor β receptor type 2 (*Tgfbr2)*	TCCTAGTGAAGAACGACTTGACC	TACCAGAGCCATGGAGTAGACAT
Collagen 1A1 (*Col1a1*)	TGAACGTGACCAAAAACCAA	GCAGAAAAGGCAGCATTAGG
Collagen 1A2 (*Col1a2*)	AGGCAGGTCTGGGCTTTATT	CGTATCCACAAAGCTGAGCA
Collagen 4A1 (*Col4a1*)	CAGGCATAGTCAGACAACAGATG	TGGACAGCCAGTAAGAGTAGTCG
