# Supplementary material for: Inhibition of hepatic oxalate overproduction ameliorates metabolic dysfunction-associated steatohepatitis
Source: Nat Metab. 2024 Sep 27;6(10):1939–62. doi: 10.1038/s42255-024-01134-4 (PMC11495999; doi:10.1038/s42255-024-01134-4)
Supplement: Supplementary file 2 — Reporting Summary [file 42255_2024_1134_MOESM2_ESM.pdf]

nature portfolio

Corresponding author(s): Oren Rom
Last updated by author(s): Aug 9, 2024

Reporting Summary

Nature Portfolio wishes to improve the reproducibility of the work that we publish. This form provides structure for consistency and transparency in reporting. For further information on Nature Portfolio policies, see our Editorial Policies and the Editorial Policy Checklist.

Statistics

For all statistical analyses, confirm that the following items are present in the figure legend, table legend, main text, or Methods section.

n/a	Confirmed	
☐	☒	The exact sample size (*n*) for each experimental group/condition, given as a discrete number and unit of measurement
☐	☒	A statement on whether measurements were taken from distinct samples or whether the same sample was measured repeatedly
☐	☒	The statistical test(s) used AND whether they are one- or two-sided<br>*Only common tests should be described solely by name; describe more complex techniques in the Methods section.*
☐	☒	A description of all covariates tested
☐	☒	A description of any assumptions or corrections, such as tests of normality and adjustment for multiple comparisons
☐	☒	A full description of the statistical parameters including central tendency (e.g. means) or other basic estimates (e.g. regression coefficient) AND variation (e.g. standard deviation) or associated estimates of uncertainty (e.g. confidence intervals)
☐	☒	For null hypothesis testing, the test statistic (e.g. *F*, *t*, *r*) with confidence intervals, effect sizes, degrees of freedom and *P* value noted<br>*Give P values as exact values whenever suitable.*
☒	☐	For Bayesian analysis, information on the choice of priors and Markov chain Monte Carlo settings
☒	☐	For hierarchical and complex designs, identification of the appropriate level for tests and full reporting of outcomes
☒	☐	Estimates of effect sizes (e.g. Cohen's *d*, Pearson's *r*), indicating how they were calculated

Our web collection on statistics for biologists contains articles on many of the points above.

Software and code

Policy information about availability of computer code

Data collection	Reduced representation bisulfite sequencing (RRBS): methylKit R package (Bismark-0.23.0, bowtie2-2.4.2, methylKit_1.28.0), RNA sequencing: NovaSeq Control Software v1.8.0; RTA v3 (primary analysis), FastQC v0.11.8 (quality analysis of raw FASTQ files), Trimmomatic v.0.35 (trimming of low-quality reads), HISAT2 v.2.1.0.13 (mapping to the mouse reference genome, GRCm38.90), R package DESeq2_1.42.1 (identification of significant differentially expressed genes), clusterProfiler_4.10.1 (KEGG pathway analysis). Lipidomics: Thermo Scientific LipidSearch software (version 5.1). Immunohistochemical and immunofluorescence analyses: Keyence BZ-X800 analyzer software. Western blot: Li-Cor Odyssey XF Imager and Image Studio Lite v5.2 software.
Data analysis	GraphPad Prism v10 software

For manuscripts utilizing custom algorithms or software that are central to the research but not yet described in published literature, software must be made available to editors and reviewers. We strongly encourage code deposition in a community repository (e.g. GitHub). See the Nature Portfolio guidelines for submitting code & software for further information.

Data

Policy information about availability of data

All manuscripts must include a data availability statement. This statement should provide the following information, where applicable:

- Accession codes, unique identifiers, or web links for publicly available datasets
- A description of any restrictions on data availability
- For clinical datasets or third party data, please ensure that the statement adheres to our policy

All data are available within the manuscript, extended data, source data files and supplementary files. Raw RNA-sequencing and RRBS data have been deposited in NCBI's GEO database under accession numbers GSE224097 and GSE265985, respectively. Raw lipidomics data have been deposited in MassIVE under accession number MSV000094587.

Research involving human participants, their data, or biological material

Policy information about studies with human participants or human data. See also policy information about sex, gender (identity/presentation), and sexual orientation and race, ethnicity and racism.

Reporting on sex and gender	The term sex was carefully used as a biological attribute. The findings apply to both males and females. Sex was determined based on self-report, demographics and clinical information obtained at the Multi-Organ Transplant Institute, Ochsner Clinic Foundation (New Orleans, LA, USA) and BioIVT, LLC (Kansas City, United States). The collection of deidentified human liver and plasma specimens was approved by the Institutional Review Board of Ochsner Clinic Foundation (protocols 2010.179, 2016.131.B, and 2020.039). The studies were conducted through the Ochsner Multi-Organ Transplant Institute with specimens collected following informed consent. Liver and plasma oxalate concentrations as well as hepatic expression of genes regulating glyoxylate metabolism or oxalate formation among patients with or without MASH were not analyzed based on sex due to 1) the low sample size, and 2) no significant differences in sex (or other potential confounders such as age and race) between the patients with or without MASH.
Reporting on race, ethnicity, or other socially relevant groupings	Race and age were determined based on self-report, demographics and clinical information obtained at the Multi-Organ Transplant Institute, Ochsner Clinic Foundation (New Orleans, LA, USA) and BioIVT, LLC (Kansas City, United States). The collection of deidentified human liver and plasma specimens was approved by the Institutional Review Board of Ochsner Clinic Foundation (protocols 2010.179, 2016.131.B, and 2020.039). The studies were conducted through the Ochsner Multi-Organ Transplant Institute with specimens collected following informed consent. Liver and plasma oxalate concentrations as well as hepatic expression of genes regulating glyoxylate metabolism or oxalate formation among patients with or without MASH were not analyzed based on race and ethnicity due to 1) the low sample size, and 2) no significant differences in race between the patients with or without MASH.
Population characteristics	For liver specimens, the sex distribution (M/F), age (mean ± SEM), and race distribution (Caucasian/African American/Asian/Latino-Hispanic) among the patients with MASH were 14/9, 62.13 ± 1.75, and 22/1/0/0, respectively. The sex distribution (M/F), age (mean ± SEM), and race distribution (Caucasian/African American/Asian/Latino-Hispanic) among the patients without MASH were 4/6, 60.60 ± 2.01, and 6/2/1/1, respectively. There were no significant differences in sex, age, or race between the patients with or without MASH. For plasma specimens, the sex distribution (M/F), age (mean ± SEM), and race distribution (Caucasian/African American/Latino-Hispanic/Asian) among the patients with NASH were 10/17, 59.81 ± 1.42, and 25/1/1/0. The sex distribution (M/F), age (mean ± SEM), and race distribution (Caucasian/African American/Latino-Hispanic/Asian) among the patients without MASH were 6/26, 62.44 ± 1.85, and 22/6/2/2, respectively. There were no significant differences in sex, age, or race between the patients with or without MASH.
Recruitment	All available samples were analyzed. No potential self-selection bias or any other biases to report.
Ethics oversight	The studies of deidentified human liver and plasma specimens were approved by the Institutional Review Board of Ochsner Clinic Foundation (protocols 2010.179, 2016.131.B, and 2020.039).

Note that full information on the approval of the study protocol must also be provided in the manuscript.

Field-specific reporting

Please select the one below that is the best fit for your research. If you are not sure, read the appropriate sections before making your selection.

☒ Life sciences ☐ Behavioural & social sciences ☐ Ecological, evolutionary & environmental sciences

For a reference copy of the document with all sections, see nature.com/documents/nr-reporting-summary-flat.pdf

Life sciences study design

All studies must disclose on these points even when the disclosure is negative.

Sample size	The sample size for the mouse and in vitro studies was determined based on power analysis (WINPEPI software) with a 5% significance level and 80% power using data from our previous studies on MASH and AGXT (Sci Transl Med. 2020, PMID: 33268508; Cell Rep. 2021, PMID:

34320345; Cell Metab. 2023. PMID: 37040763). The sample size for the human study was not predetermined. All available human samples were analyzed.

Data exclusions	No data were excluded from the analyses.
Replication	The reproducibility of the findings was verified in liver samples from patients with MASH, multiple mouse models of MASH, mouse primary hepatocytes and the HepG2 human hepatoma cell line. Findings from humans, mice, mouse primary hepatocytes and HepG2 cells were replicated using at least 3 independent and biologically distinct samples.
Randomization	Randomization was not relevant to the human data due to the observational study design. C57BL/6J mice ordered from the Jackson Laboratories were randomly selected to receive either AAV8-TBG-AGXT expressing human AGXT or AAV8-TNG-GFP control driven by the hepatocyte-specific TBG promoter. There were no significant differences in body weight between the groups before or after AAV8 injection. C57BL/6J ordered from the Jackson Laboratories were randomly selected to receive vehicle, 5 mg/kg/day MDMG-935P, or 10 mg/kg/day MDMG-935P. There were no significant differences in body weight between the groups before or after MDMG-935P treatment.
Blinding	The following analyses were conducted by technicians or investigators blinded to experimental groups: histopathology, RNA-sequencing, lipidomics, IC-MS, and RRBS. Other data collection and analyses were not performed blindly due to the complexity of the experiments or because they were conducted by a single investigator. However, the use of software and machine-based analysis minimized potential investigator bias.

Reporting for specific materials, systems and methods

We require information from authors about some types of materials, experimental systems and methods used in many studies. Here, indicate whether each material, system or method listed is relevant to your study. If you are not sure if a list item applies to your research, read the appropriate section before selecting a response.

Materials & experimental systems

n/a	Involved in the study	
☐	☒	Antibodies
☐	☒	Eukaryotic cell lines
☒	☐	Palaeontology and archaeology
☐	☒	Animals and other organisms
☒	☐	Clinical data
☒	☐	Dual use research of concern
☒	☐	Plants

Methods

n/a	Involved in the study	
☒	☐	ChIP-seq
☒	☐	Flow cytometry
☒	☐	MRI-based neuroimaging

Antibodies

Antibodies used	Anti-AGXT (Sigma; #HPA035370, lot: B119109; and Santa Cruz, #SC-517388, lot: #G3021), anti-CPT1α (Abcam, #ab234111, lot: GR3351270-28), anti-β-actin (Cell Signaling Technology, #3700S, lot: 13), anti-GAPDH (Santa Cruz Biotechnology, #sc365062, lot:#K0321), anti-F4/80 (Bio-Rad ABD Serotec, #MCA497R, lot: 159786), anti-CCR2 (Abcam, #ab273050, lot: 1003175-20), anti-TREM2 (Proteintech ,#27599-1-AP, lot: 00055044), anti-Ly6g (Thermo scientific, #11-9668-82, lot: 2750881), anti-PPARA (Proteintech, #15540-1-AP, lot: 00110519), anti-HAO1 (Abcam, #194790, lot: 1090660-1), anti-LDHA (Cell signaling technology, #2012, lot: 2), anti-smooth muscle actin-Cy3 (Sigma, #C6198, lot: 0000214427), anti-arginase1 (Arg1, Sigma #HPA024006), and goat-anti-rat secondary antibody conjugated to Cy5 fluorophore (Invitrogen, #A-21247).
Validation	We validated the anti-AGXT antibodies (Sigma #HPA035370, and Santa Cruz #SC-517388) using liver samples from mice that are either deficient in AGXT (described in Sci Transl Med. 2020, PMID: 33268508) or overexpressing the human AGXT in hepatocytes (described in Cell Rep. 2021, PMID: 34320345, and in the current manuscript).<br><br>The anti-CPT1α antibody (Abcam #ab234111) was validated in human and mouse tissues using WB and IHC. Loss of signal was observed when knockout cell line ab266319 (knockout cell lysate ab256880) was used. This antibody was referenced in 29 publications. https://www.abcam.com/en-us/products/primary-antibodies/cpt1a-antibody-epr21843-71-2f-ab234111#.<br><br>The anti-β-actin antibody (Cell Signaling Technology #3700S) was validated in human and mouse tissues and cells using WB, IHC, IF and flow cytometry. This antibody was referenced in 4458 publications. https://www.cellsignal.com/products/primary-antibodies/b-actin-8h10d10-mouse-mab/3700<br><br>The anti-GAPDH antibody (Santa Cruz Biotechnology #sc365062) was validated in human and mouse tissues and cells using WB and IHC. This antibody was referenced in 1,534 publications. https://www.scbt.com/p/gapdh-antibody-g-9?gad_source=1&gclid=CjwKCAjw_Na1BhAlEiwAM-dm7LckvoSxfVfuqJrK37Ud-vChzBKHZqfl4-VY6oDrS-h00-ktrg6e4xoCsFMQAvD_BwE<br><br>The anti-F4/80 antibody (Bio-Rad ABD Serotec #MCA497R) was validated in mouse tissue using WB, IHC, IF and flow cytometry. This antibody was referenced in 298 publications. https://www.bio-rad-antibodies.com/monoclonal/mouse-f4-80-antibody-cl-a3-1-mca497.html?f=purified<br><br>The anti-smooth muscle actin-Cy3 antibody (Sigma #C6198) was validated in human and mouse tissues using IF. This antibody was referenced in 47 publications. https://www.sigmaaldrich.com/US/en/product/sigma/c6198?utm_source=google&utm_medium=cpc&utm_campaign=21466469979&utm_content=165394577192&gclid=CjwKCAjw_Na1BhAlEi

wAM-dm7APCkDkfKeIvoeEVcnD1siRQ7a2a9xR_NzcO9q6lAElUDCfeAaXOSxoCFFwQAvD_BwE

The anti-PPARA antibody (Proteintech #15540-1-AP) was validated in human and mouse cells using WB and IP. This antibody was referenced in 200 publications. https://www.ptglab.com/products/PPARA-Antibody-15540-1-AP.htm

The anti-HAO1 (Abcam# 194790) was validated in mouse tissue using WB. https://www.abcam.com/en-us/products/primary-antibodies/hao1-gox-antibody-ab194790#

The anti-LDHA antibody (Cell signaling technology #2012) was validated in human and mouse cells using WB. This antibody was referenced in 200 publications. https://www.cellsignal.com/products/primary-antibodies/ldha-antibody/2012

The anti-arginase1 antibody (Arg1, Sigma #HPA024006) was validated in human tissues by WB and IHC. This antibody was referenced in 16 publications. https://www.sigmaaldrich.com/US/en/product/sigma/hpa003595?utm_source=google&utm_medium=cpc&utm_campaign=21466469979&utm_content=165394639632&gclid=CjwKCAjw_Na1BhAlEiwAM-dm7O5epwdz2dmTcZTDMKgTbr2C0IiWr9XNVFMYmVXD7gsyQSovcC0iyhoCkmcQAvD_BwE

The anti-CCR2 antibody (Abcam #ab273050) was validated in mouse tissues, including CCR2 KO mice, using WB. This antibody was referenced in 16 publications. https://www.abcam.com/en-us/products/primary-antibodies/ccr2-antibody-epr20844-15-ab273050#application=wb

The anti-TREM2 antibody (Proteintech # 27599-1-AP) was validated in human activated and non-activated macrophages using WB. This antibody was referenced in 18 publications. https://www.ptglab.com/products/TREM2-Antibody-27599-1-AP.htm

The anti-Ly6g antibody (Thermo scientific #11-9668-82) was validated in mouse cells using flow cytometry. This antibody was referenced in 61 publications. https://www.thermofisher.com/antibody/product/Ly-6G-Antibody-clone-1A8-Ly6g-Monoclonal/11-9668-82

Eukaryotic cell lines

Policy information about cell lines and Sex and Gender in Research

Cell line source(s)	The HepG2 human hepatoma cell line was obtained from the American Type Culture Collection (ATCC, HB-8065). The HEK293T cell line that was obtained from American Type Culture Collection (ATCC, CRL-3216).
Authentication	HepG2 cells express known hepatocyte markers, including albumin, and also express AGXT. The HEK293T was authenticated by ATCC.
Mycoplasma contamination	Cells were not tested for Mycoplasma contamination
Commonly misidentified lines (See ICLAC register)	No commonly misidentified cell lines were used in this study.

Animals and other research organisms

Policy information about studies involving animals; ARRIVE guidelines recommended for reporting animal research, and Sex and Gender in Research

Laboratory animals	C57BL/6J (stock: 000664) were purchased from the Jackson Laboratories. Eight-week-old C57BL/6J male and female mice were housed under controlled temperature (22±2˚C) and humidity conditions (40-60%) on a 12-hour light/dark cycle and fed ad libitum either a standard chow diet (LabDiet #5053, 13% of calories from fat) or established12,32-35 MASH-inducing diets (Research Diets #D17010103, 40% of calories from fat or Envigo, #TD.160785, 52.6% of calories from fat) for 12, 16, or 24 weeks prior to euthanasia and tissue harvest. Primary hepatocytes were isolated from 8-10-week-old male C57BL/6J mice fed a standard chow diet as described below. AAV8-AGXT expressing human AGXT and AAV8-GFP control driven by the hepatocyte-specific TBG promoter were administered by intraperitoneal injection into 7-week-old male C57BL/6J mice at 2x1011 viral genomes and a final volume of 200 µL per mouse, as we previously described24. Starting from 8 weeks of age, mice were fed the MASH diet (Research Diets #D17010103) ad libitum for 24 weeks. AAV8-AGXT and AAV8-TBG-GFP were similarly administered to 11-week-old male C57BL/6J mice that were kept on the standard chow (LabDiet #5053) ad libitum for an additional 12 weeks of age. MDMG-935P is a salicylic acid derivative we recently developed that potently decreases oxalate production by inhibiting GO and LDHA54. The therapeutic potential of MDMG-935P was evaluated in mice with MASH using established protocols12,32,33,54. Eight-week-old C57BL/6J male mice were fed the MASH diet (Research Diets #D17010103) ad libitum for 12 weeks. Mice were then randomized to receive MDMG-935P solubilized in 0.5% methylcellulose by oral gavage at a concentration of 0 mg/kg/day (vehicle), 5 mg/kg/day, or 10 mg/kg/day for an additional 12 weeks on the MASH diet until euthanasia and tissue harvest. Similar studies were performed in mice fed the standard chow diet. Eight-week-old C57BL/6J male mice were fed the standard chow diet (LabDiet #5053) ad libitum for 12 weeks. Mice were then randomized to receive MDMG-935P solubilized in 0.5% methylcellulose by oral gavage at a concentration of 0 mg/kg/day (vehicle) or 10 mg/kg/day for an additional 12 weeks on standard chow diet until euthanasia and tissue harvest.
Wild animals	No wild animals were used in this study.
Reporting on sex	We confirmed the suppression of AGXT in livers from both male and female mice with NASH in separate analyses (n=6 per group/sex). The AGXT overexpression and MDMG-935P studies were done in male mice.
Field-collected samples	No field-collected samples were used in the study.

Ethics oversight	All animal procedures were approved by the Institutional Animal Care & Use Committees of Louisiana State University Health Sciences Center-Shreveport (P-21-043, P22-035, and P-24-025) and the University of Michigan (PRO00008239). All studies were performed in accordance with the institutional guidelines.

Note that full information on the approval of the study protocol must also be provided in the manuscript.

Plants

Seed stocks	n/a
Novel plant genotypes	n/a
Authentication	n/a
