# Supplementary material for: Inhibition of hepatic oxalate overproduction ameliorates metabolic dysfunction-associated steatohepatitis
Source: Nat Metab. 2024 Sep 27;6(10):1939–62. doi: 10.1038/s42255-024-01134-4 (PMC11495999; doi:10.1038/s42255-024-01134-4)

Figure 1d: Protein abundance and quantification of AGXT relative to GAPDH in liver samples from in liver samples from patients with end-stage MASH (n=23) and controls (n=10).

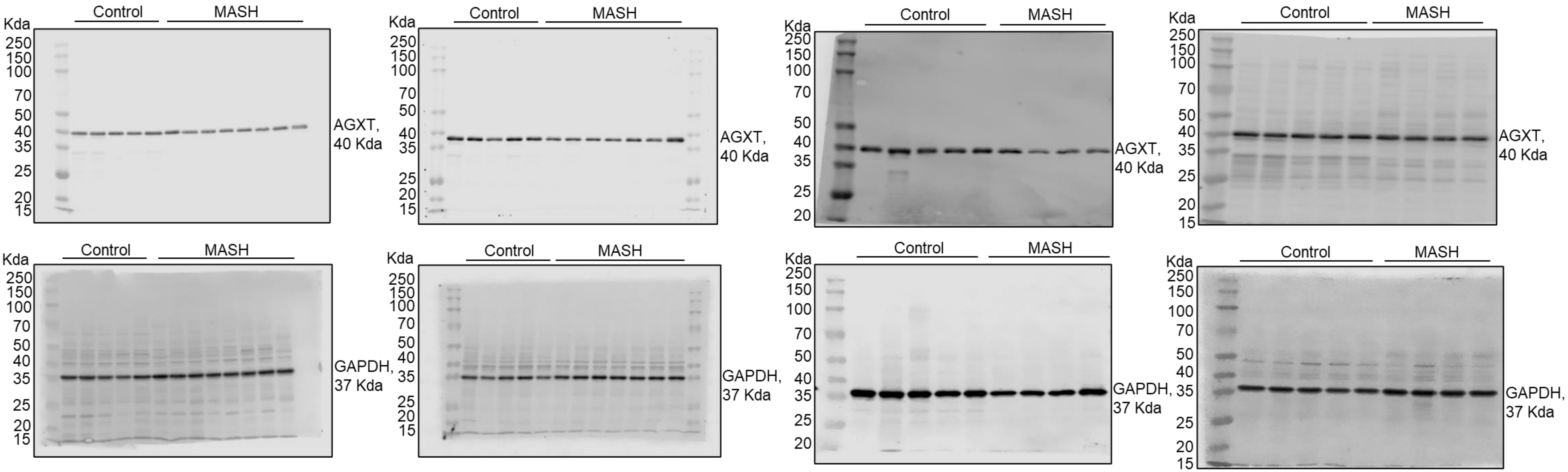

Fig 1k: Liver samples were collected from C57BL/6J mice fed a standard chow diet (Control) or a high-fat, high-fructose, high-cholesterol diet (MASH diet) for 24 weeks. Protein abundance and quantification of AGXT relative to β-Actin in liver samples from mice with or without MASH (n=6).

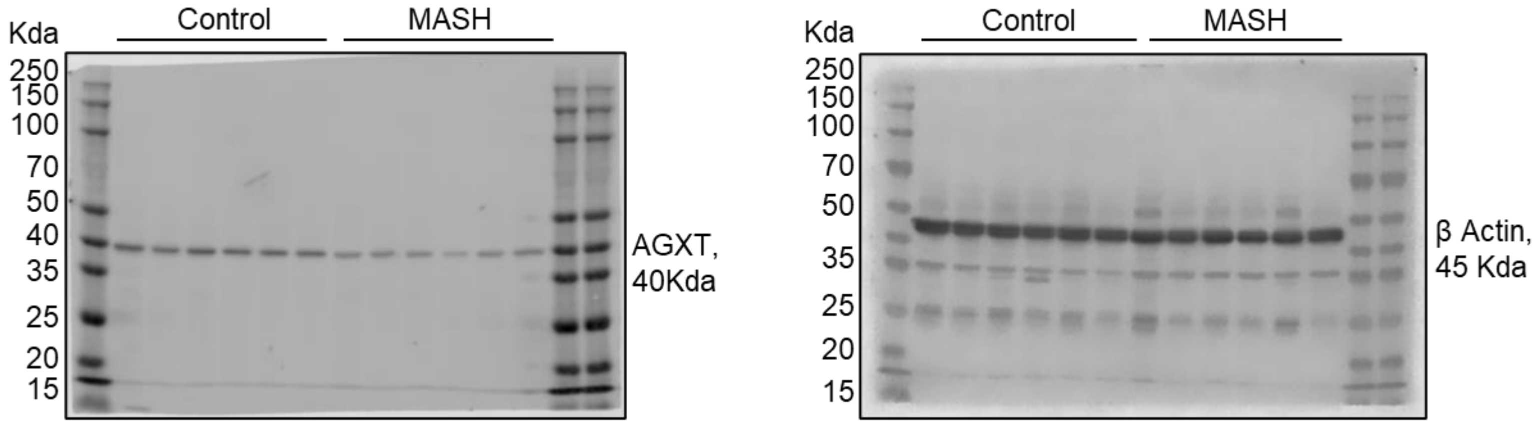

Figure 1o: Primary hepatocytes (Hep) from mice fed a standard chow diet and HepG2 cells were treated with either BSA-conjugated palmitic acid (PA, 200 µM) or BSA control overnight. Protein abundance and quantification of AGXT relative to GAPDH (Primary hepatocytes, n=5) or β-Actin (HepG2 cells, n=6).

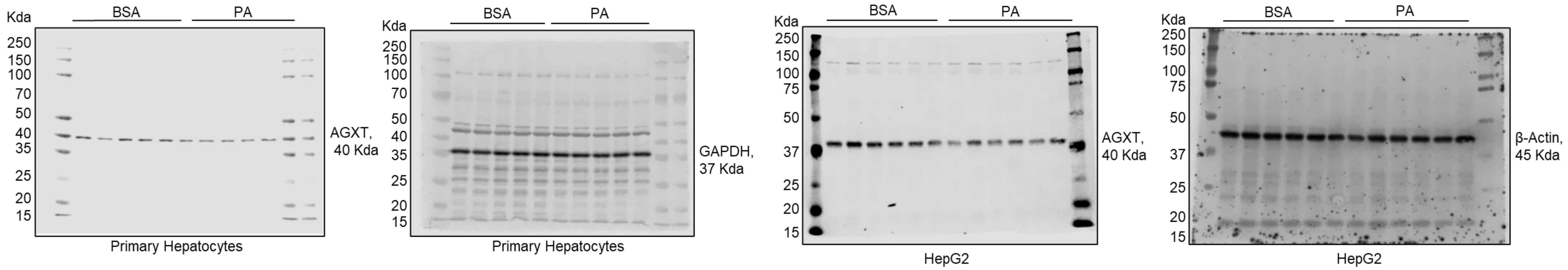

Supplement: Supplementary file 4 — Unprocessed western blots/gels. [file 42255_2024_1134_MOESM4_ESM.pdf]
