# Supplementary material for: Inhibition of hepatic oxalate overproduction ameliorates metabolic dysfunction-associated steatohepatitis
Source: Nat Metab. 2024 Sep 27;6(10):1939–62. doi: 10.1038/s42255-024-01134-4 (PMC11495999; doi:10.1038/s42255-024-01134-4)

Figure 2b: Male C57BL/6J mice were injected with AAV8-TBG-GFP or AAV8-TBG-AGXT ($2x10^{11}$ viral genomes per mouse) and placed on the MASH diet for 24 weeks prior to endpoint analyses. Protein abundance, and quantification of AGXT relative to β-Actin in liver samples from mice treated with AAV8-GFP or AAV-AGXT (n=4).

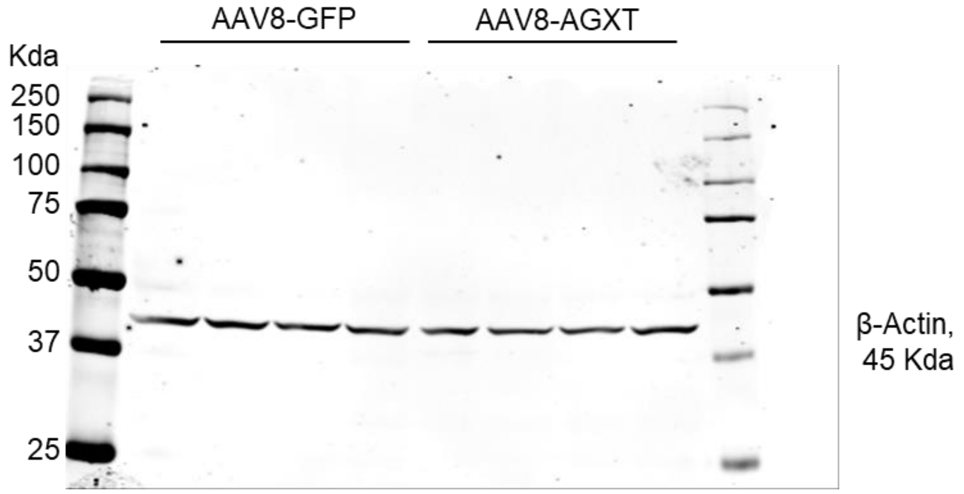

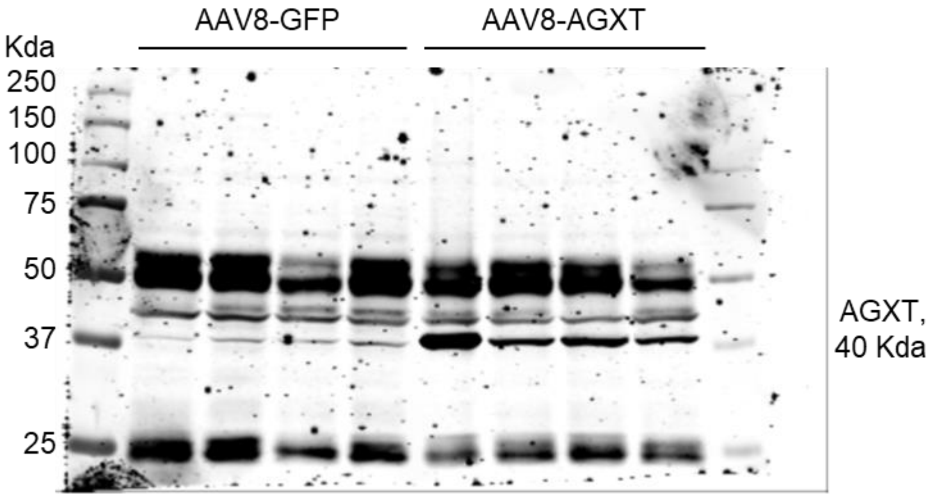

Supplement: Supplementary file 6 — Unprocessed western blots/gels. [file 42255_2024_1134_MOESM6_ESM.pdf]
