# Supplementary material for: Inhibition of hepatic oxalate overproduction ameliorates metabolic dysfunction-associated steatohepatitis
Source: Nat Metab. 2024 Sep 27;6(10):1939–62. doi: 10.1038/s42255-024-01134-4 (PMC11495999; doi:10.1038/s42255-024-01134-4)

Fig 4e: Protein abundance and quantification of CPT1α relative to GAPDH in HepG2 cells treated with and without NaOX overnight and expressed as fold change from control (without NaOX) (n=6).

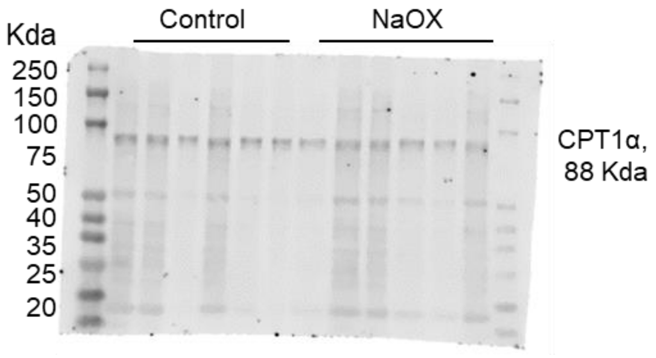

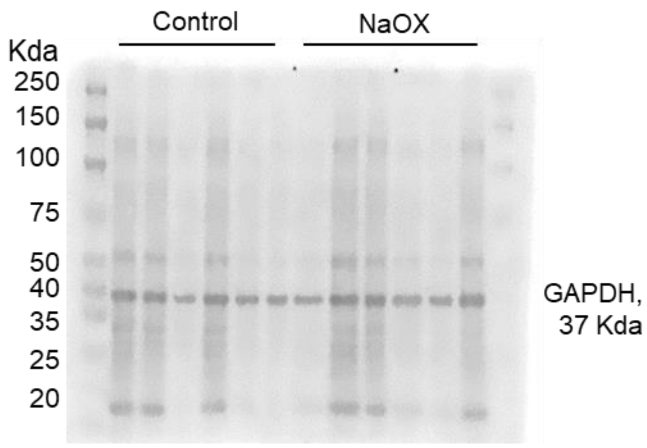

Fig 4 l: HepG2 cells were transfected with either GFP control (GFP) or GFP-tagged AGXT (AGXT) plasmids. Western blot analysis for AGXT protein abundance 48 h post-transfection (n=3)

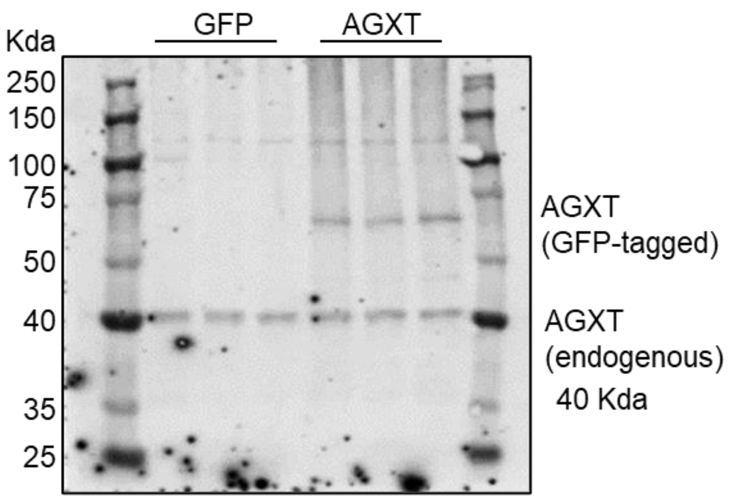

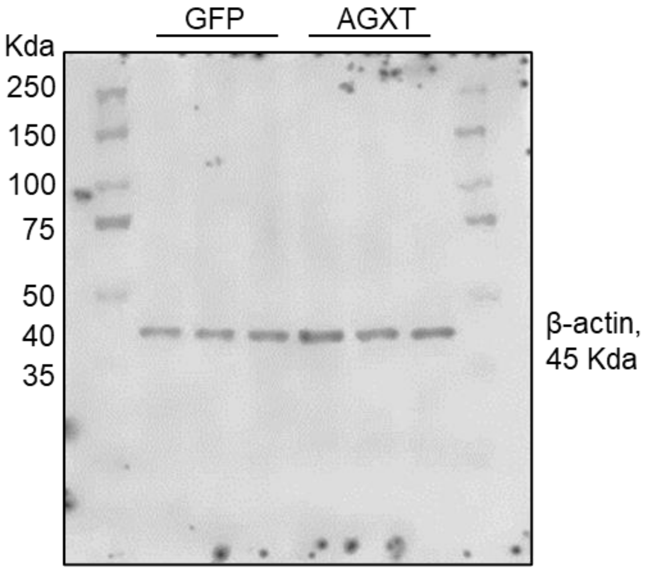

Fig 4p: HepG2 cells were transfected with either GFP control (GFP) or GFP-tagged AGXT (AGXT) plasmids. After 24 h, the cells were treated with BSA-conjugated palmitic acid (PA, 200 μM) overnight followed by analysis of protein abundance of CPT1α relative to GAPDH (n=5).

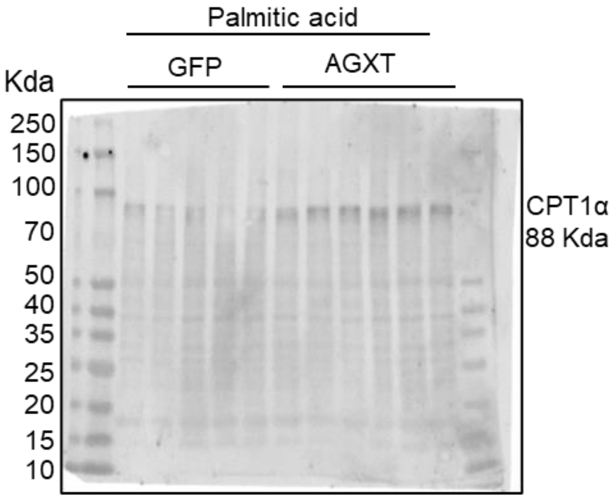

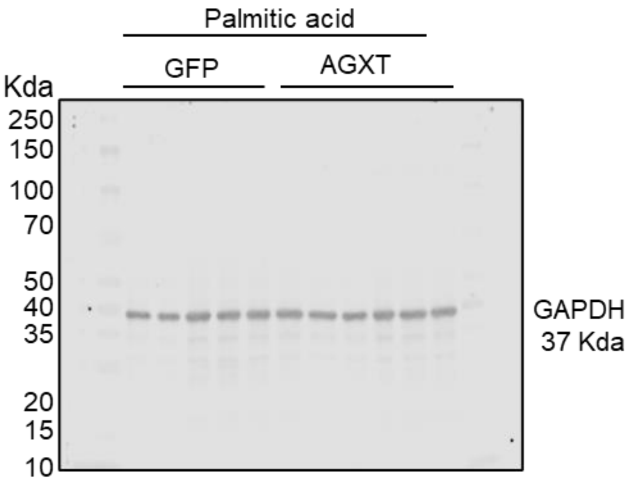

Supplement: Supplementary file 9 — Unprocessed western blots/gels. [file 42255_2024_1134_MOESM9_ESM.pdf]
