# Supplementary material for: Inhibition of hepatic oxalate overproduction ameliorates metabolic dysfunction-associated steatohepatitis
Source: Nat Metab. 2024 Sep 27;6(10):1939–62. doi: 10.1038/s42255-024-01134-4 (PMC11495999; doi:10.1038/s42255-024-01134-4)

Fig 1b: Protein abundance and quantification of LDHA relative to GAPDH in liver specimens from patients with MASH (n=23) or controls (n=10).

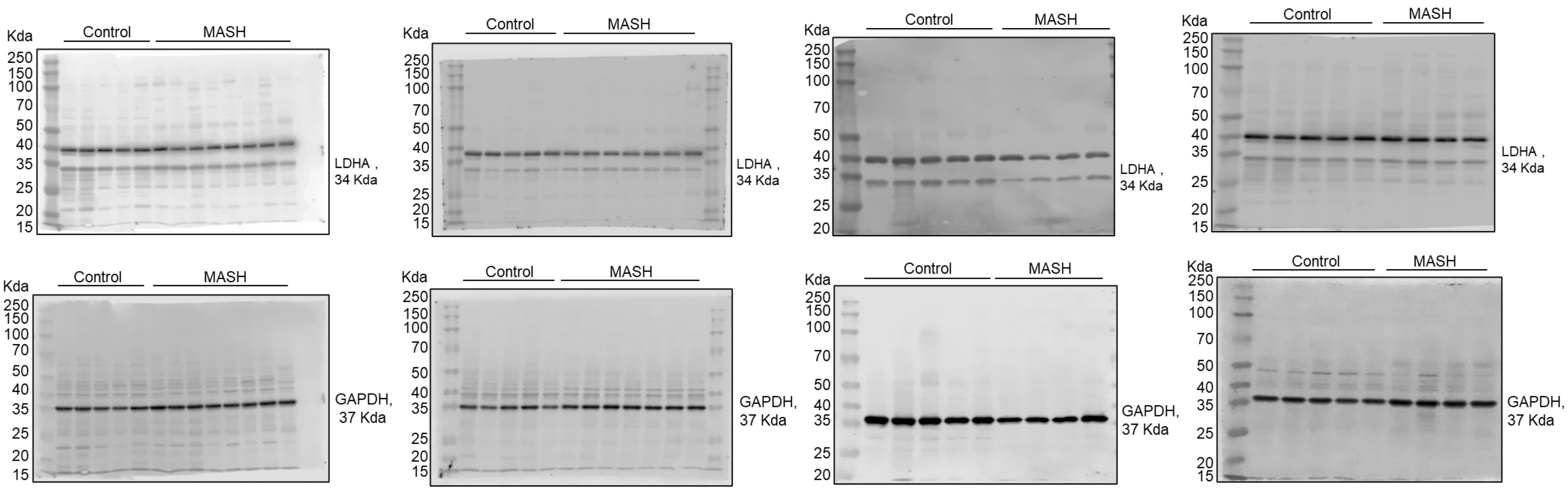

Supplement: Supplementary file 15 — Unprocessed western blots/gels. [file 42255_2024_1134_MOESM15_ESM.pdf]
