# Supplementary material for: Inhibition of hepatic oxalate overproduction ameliorates metabolic dysfunction-associated steatohepatitis
Source: Nat Metab. 2024 Sep 27;6(10):1939–62. doi: 10.1038/s42255-024-01134-4 (PMC11495999; doi:10.1038/s42255-024-01134-4)

Fig 2 d and f: Liver samples were collected from C57BL/6J mice fed a standard chow diet (Control) or a high-fat, high-fructose, high-cholesterol diet (MASH diet) for 12 weeks. Protein abundance and quantification of AGXT, and LDHA relative to β-Actin (n=6).

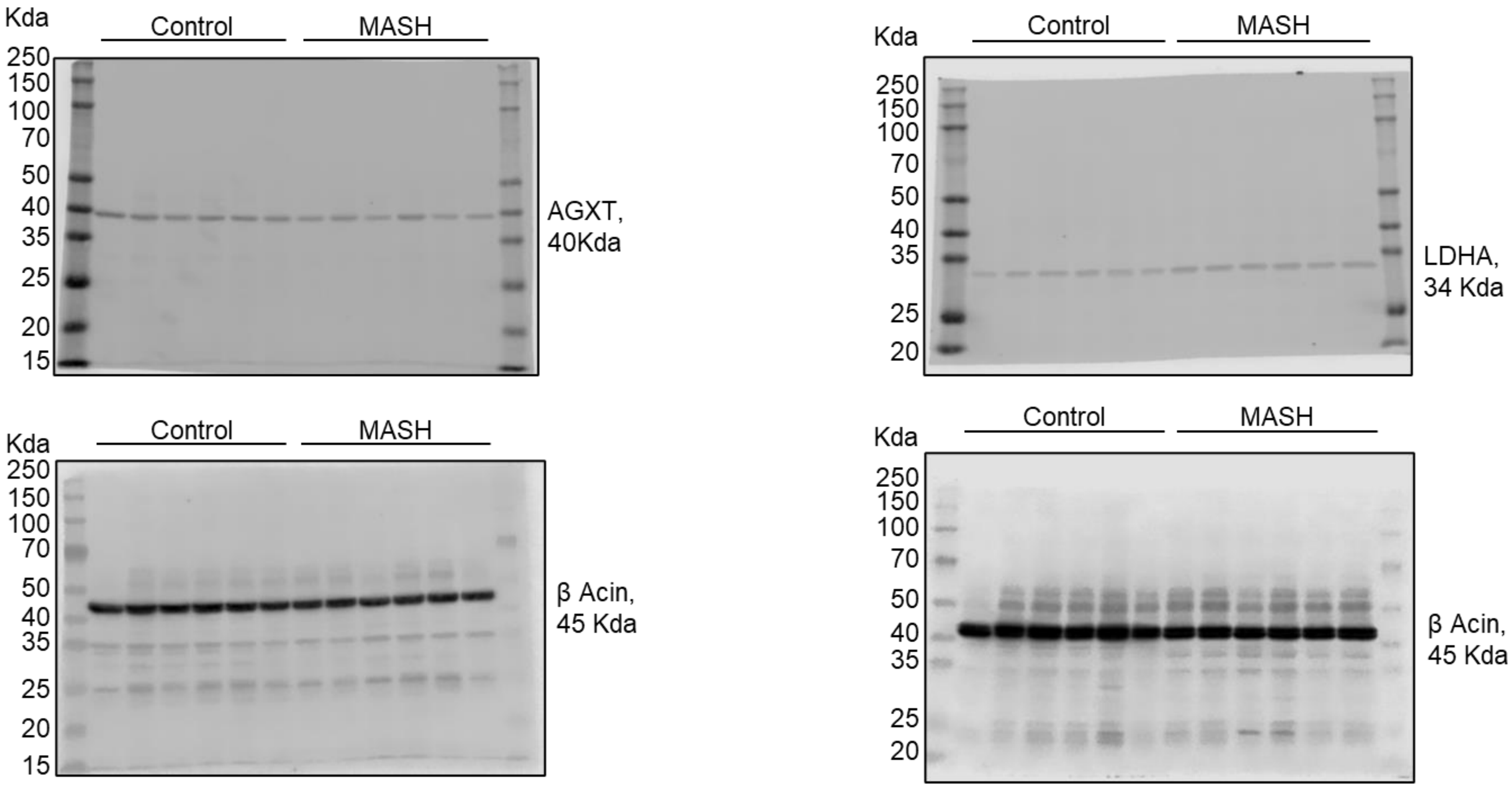

Fig 2 j: Protein abundance and quantification of LDHA relative to β Actin in liver samples from mice with or without advanced MASH (24 weeks, n=6).

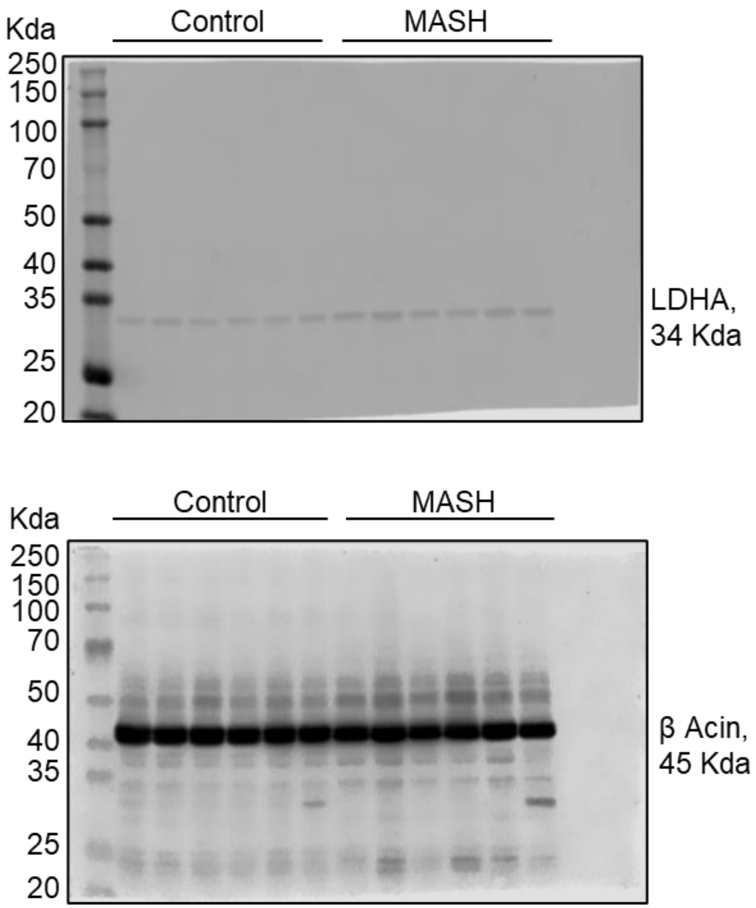

Supplement: Supplementary file 17 — Unprocessed western blots/gels. [file 42255_2024_1134_MOESM17_ESM.pdf]
