# Supplementary material for: Inhibition of hepatic oxalate overproduction ameliorates metabolic dysfunction-associated steatohepatitis
Source: Nat Metab. 2024 Sep 27;6(10):1939–62. doi: 10.1038/s42255-024-01134-4 (PMC11495999; doi:10.1038/s42255-024-01134-4)

Fig 3 c: Liver samples were collected from male mice fed a standard chow diet (Control) or the fructose-palmitate-cholesterol (FPC) diet for 4 months. Protein abundance and quantification of AGXT relative to GAPDH in livers from males with and without MASH (n=6).

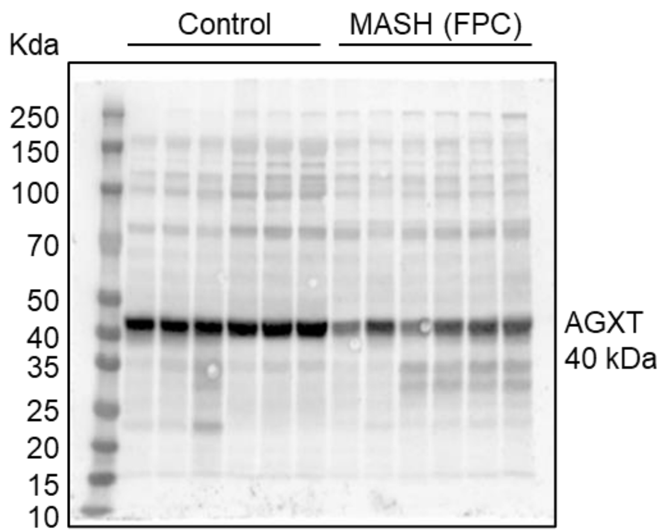

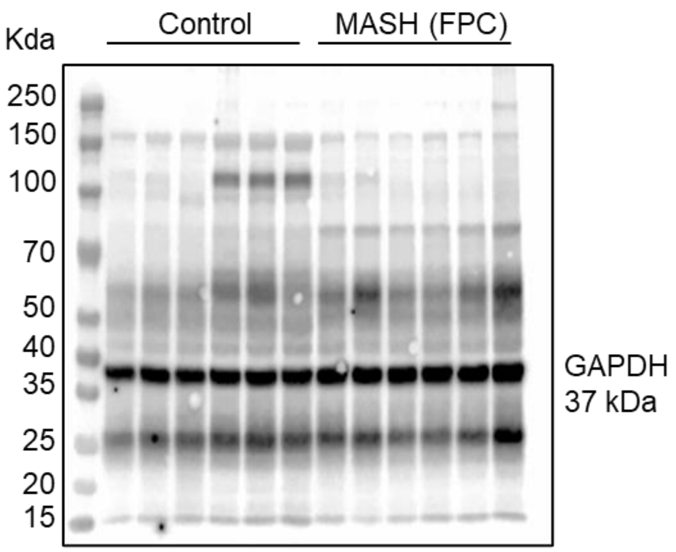

Fig 3 e:Liver samples were collected from female mice fed a standard chow diet (Control) or the fructose-palmitate-cholesterol (FPC) diet for 4 months. Protein abundance and quantification of AGXT relative to GAPDH in livers from females with and without MASH (n=6).

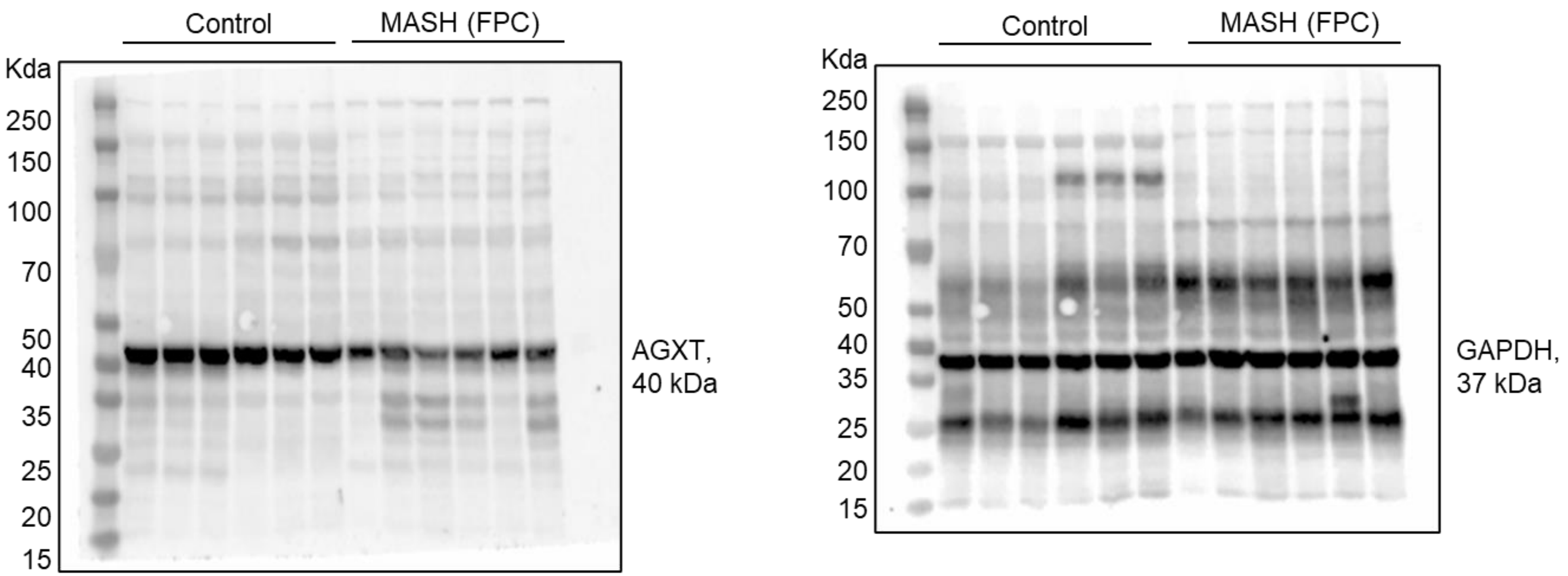

Supplement: Supplementary file 19 — Unprocessed western blots/gels. [file 42255_2024_1134_MOESM19_ESM.pdf]
