# Supplementary material for: Inhibition of hepatic oxalate overproduction ameliorates metabolic dysfunction-associated steatohepatitis
Source: Nat Metab. 2024 Sep 27;6(10):1939–62. doi: 10.1038/s42255-024-01134-4 (PMC11495999; doi:10.1038/s42255-024-01134-4)

Fig 4 b: Male C57BL/6J mice were injected with AAV8-TBG-GFP or AAV8-TBG-AGXT ($2x10^{11}$ viral genomes per mouse) and placed on the MASH diet for 24 weeks prior to endpoint analyses. Kidney lysates from mice injected with AAV8-GFP or AAV8-AGXT were assessed for AGXT by Western blot (n=4). Equal protein loading was verified using GAPDH expression and Ponceau S staining.

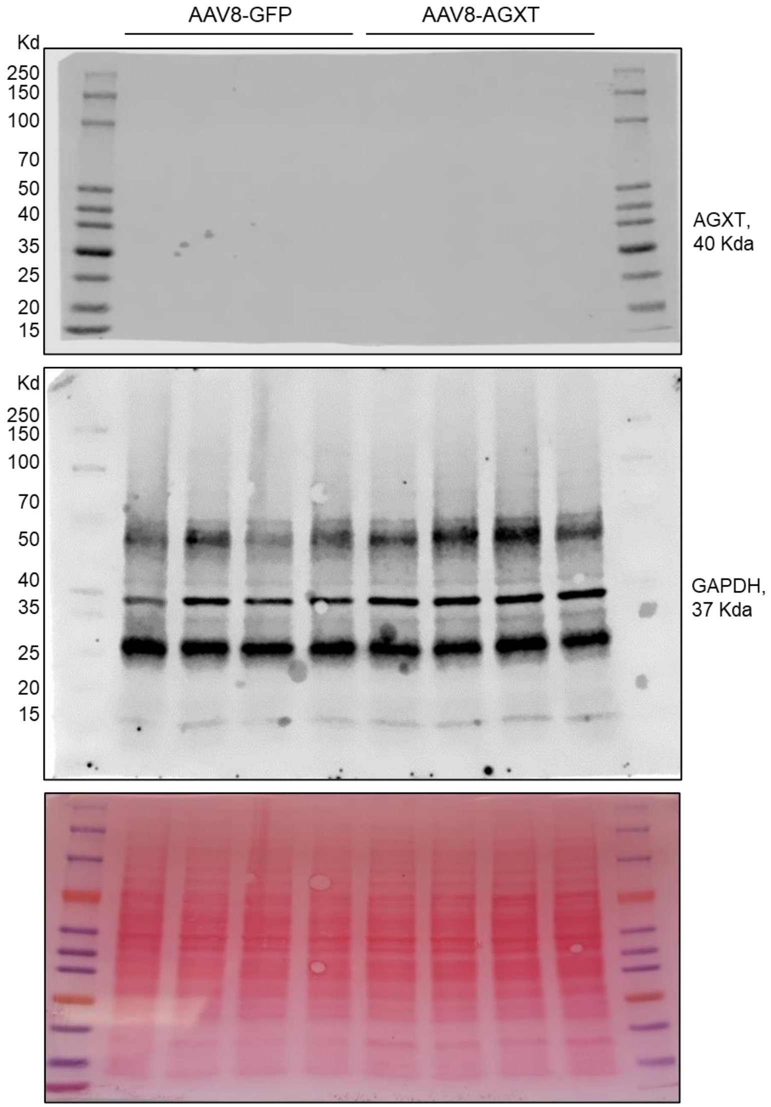

Fig 4 e: Male C57BL/6J mice were injected with AAV8-TBG-GFP or AAV8-TBG-AGXT ($2x10^{11}$ viral genomes per mouse) and placed on the standard chow diet for 12 weeks prior to endpoint analyses. Protein abundance and quantification of AGXT relative to β-Actin in liver samples from mice treated with AAV8-GFP (n=8) or AAV8-AGXT (n=10).

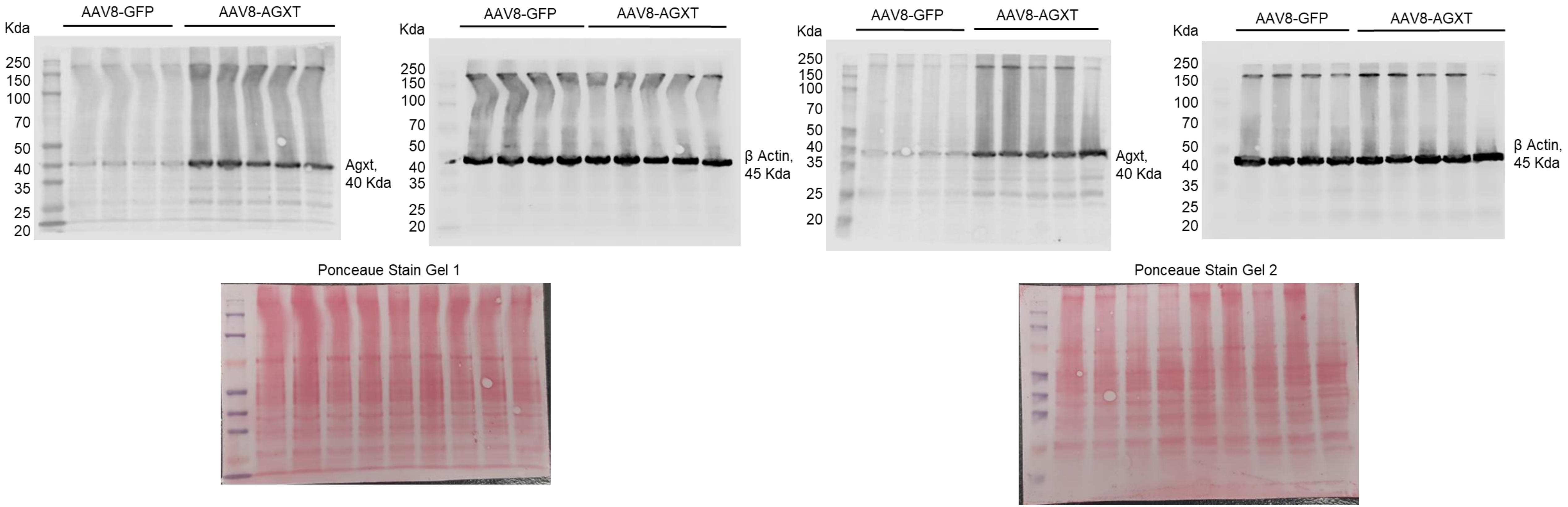

Supplement: Supplementary file 21 — Unprocessed western blots/gels. [file 42255_2024_1134_MOESM21_ESM.pdf]
