# Supplementary material for: Inhibition of hepatic oxalate overproduction ameliorates metabolic dysfunction-associated steatohepatitis
Source: Nat Metab. 2024 Sep 27;6(10):1939–62. doi: 10.1038/s42255-024-01134-4 (PMC11495999; doi:10.1038/s42255-024-01134-4)

Fig 7 b: HepG2 cells were transfected with either GFP control (GFP) or PPARα plasmids. After 48 h, the cells were lysed for Western blot analysis of PPARα relative to GAPDH (n=4).

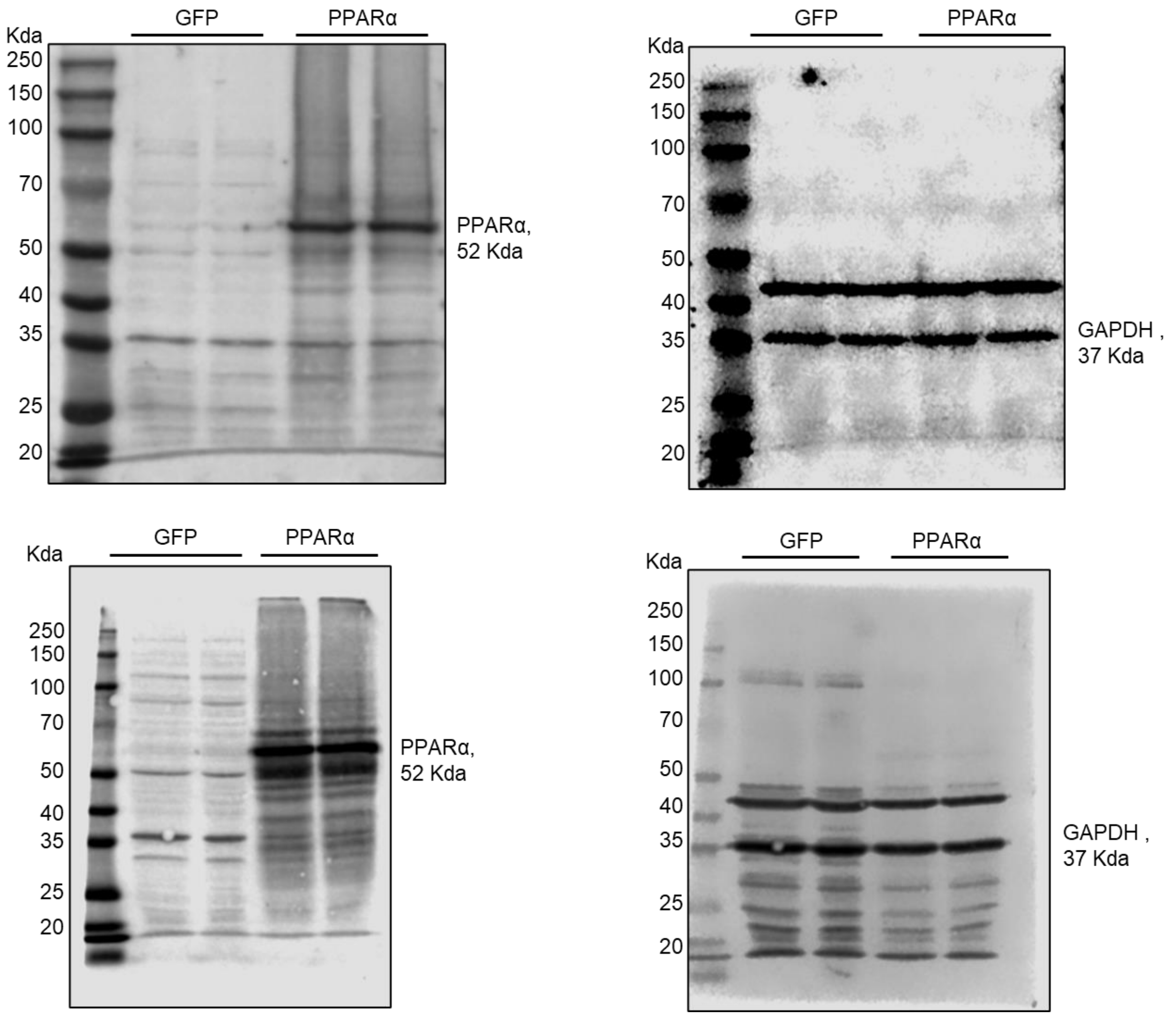

Supplement: Supplementary file 25 — Unprocessed western blots/gels. [file 42255_2024_1134_MOESM25_ESM.pdf]
