# Supplementary material for: Inhibition of hepatic oxalate overproduction ameliorates metabolic dysfunction-associated steatohepatitis
Source: Nat Metab. 2024 Sep 27;6(10):1939–62. doi: 10.1038/s42255-024-01134-4 (PMC11495999; doi:10.1038/s42255-024-01134-4)

Fig 10 c and d: Male C57BL/6J mice were fed the MASH diet for 12 weeks, then orally administered vehicle (n=7), 5 mg/kg/day (n=8) or 10 mg/kg/day (n=10) of MDMG-935P for an additional 12 weeks on the MASH diet prior to endpoint analyses. Protein abundance of GO and LDHA relative to βActin in liver samples from mice treated with vehicle, 5 mg/kg/day or 10 mg/kg/day of MDMG-935P.

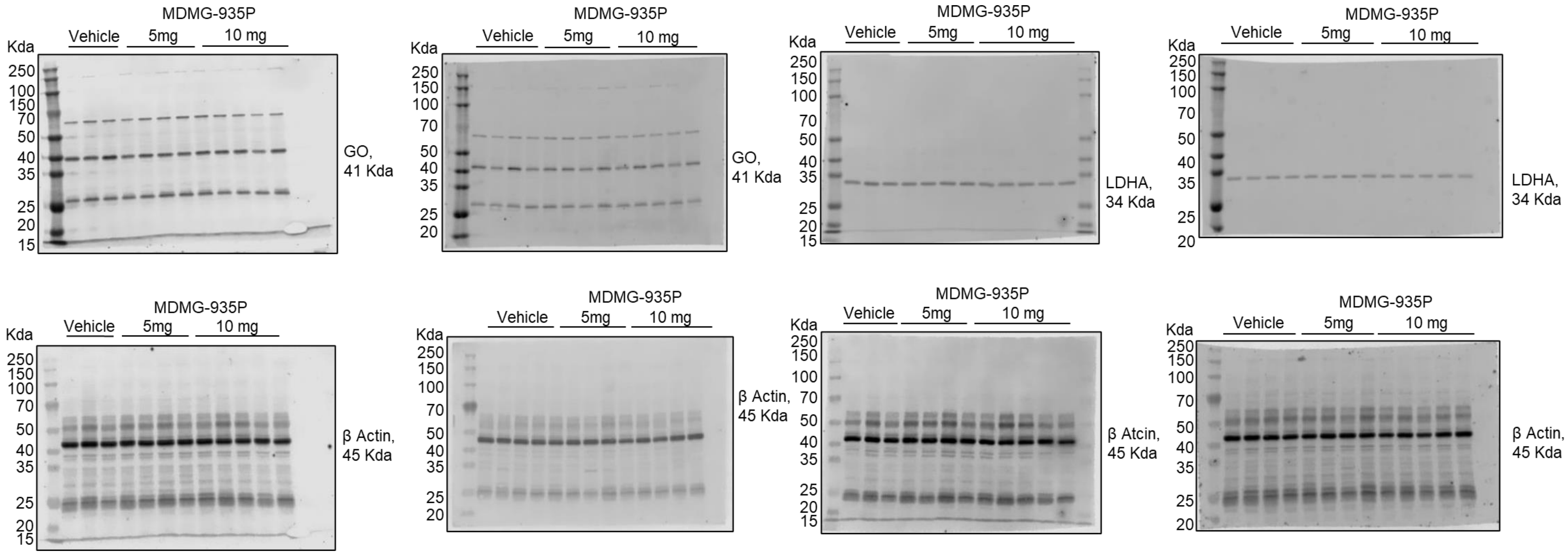

Supplement: Supplementary file 29 — Unprocessed western blots/gels. [file 42255_2024_1134_MOESM29_ESM.pdf]
